# Enantiospecific Trifluoromethyl‐Radical‐Induced Three‐Component Coupling of Boronic Esters with Furans

**DOI:** 10.1002/anie.201611058

**Published:** 2017-01-18

**Authors:** Yahui Wang, Adam Noble, Christopher Sandford, Varinder K. Aggarwal

**Affiliations:** ^1^ School of ChemistryUniversity of BristolCantock's CloseBristolBS8 1TSUK

**Keywords:** boronic esters, cross-coupling, furans, stereospecificity, trifluoromethylation

## Abstract

In the presence of trifluoromethylsulfonium reagents, boronate complexes derived from 2‐lithio furan and non‐racemic secondary and tertiary alkyl or aryl boronic esters undergo deborylative three‐component coupling to give the corresponding 2,5‐disubstituted furans with excellent levels of enantiospecificity. The process proceeds via the reaction of boronate complexes with a trifluoromethyl radical, which triggers 1,2‐metallate rearrangement upon single‐electron oxidation. Alternative electrophiles can also be used in place of trifluoromethylsulfonium reagents to effect similar three‐component coupling reactions.

Transition‐metal‐catalyzed stereoselective sp^2^‐sp^3^ cross‐coupling reactions of secondary alkyl organoboron reagents with aryl halides attract considerable interest owing to the importance of populating drug‐discovery libraries with molecules containing 3D structural motifs.^1^ However, the slow rates of both the transmetalation and the reductive elimination steps associated with these types of cross‐coupling reactions have hindered progress. Although several advances have been charted in this challenging area,^2^ we recently developed a conceptually different, transition‐metal‐free cross‐coupling reaction,^3^ which enables the stereospecific coupling of non‐racemic secondary and tertiary alkylboronic esters^4^, ^5^ with a wide range of aryl lithium reagents (Scheme 1 A). The reaction involves initial formation of aryl boronate **I** through Li–B exchange of an aryl lithium and an alkylboronic ester.^6^ Subsequent addition of an electrophilic halogenating agent (NBS=*N*‐bromosuccinimide) to **I** forms oxocarbenium **II** (structure drawn for clarity although a concerted mechanism has been proposed),3b which promotes a 1,2‐metallate rearrangement giving the neutral boronic ester **III**. Finally, rearomatization‐driven elimination of the halide and the boronic ester group generates the substituted aryl product. We reasoned that this useful methodology could be substantially expanded by using carbon‐based electrophiles in place of halogenating agents, as then the intermediate akin to boronic ester **III** (boronic ester **V**, Scheme 1 B) would not undergo elimination, thus enabling not one, but two C−C bonds to be formed in the process. Subsequent oxidative rearomatization would lead to high‐value aromatic products derived from three components.

**Scheme 1:**
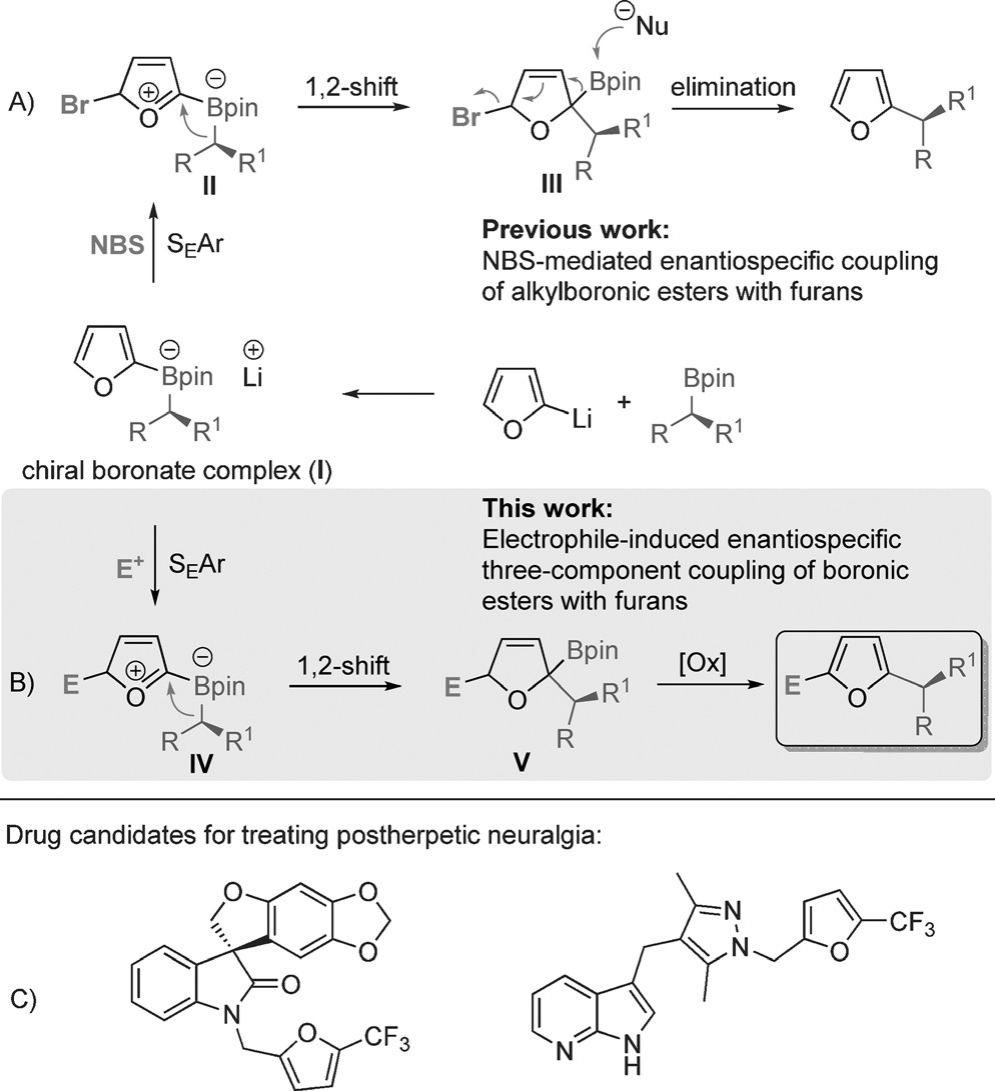
Electrophile‐induced enantiospecific three‐component coupling of boronic esters with furans.

We initially considered using trifluoromethyl‐based electrophiles because this group has found widespread utility in pharmaceuticals, endowing molecules with more attractive levels of bioavailability and membrane permeability relative to the hydrocarbon‐based parent compounds.^7^ Indeed, new methods to introduce the trifluoromethyl group have received considerable attention in recent years.^8^ We recognized the potential application of electrophilic trifluoromethylation^9^ in our proposed three‐component coupling reaction, which would provide access to enantioenriched trifluoromethylated furans, a motif that has been incorporated into potential drug candidates targeting the treatment of postherpetic neuralgia (Scheme 1 C).^10^

We began by investigating a range of commercially available trifluoromethylating agents and found that trifluoromethyldibenzothiophenium salt **A** (Umemoto's reagent) was optimum.^11^, ^12^ Thus, reaction of **A** with the boronate complex generated from furan‐2‐yllithium and cyclohexylboronic ester **1 a** gave the desired product **2 a** (ca. 1:1 d.r.) in 60 % yield (Table 1, entry 1). A solvent exchange from THF to MeCN proved essential as only a trace amount of **2 a** was observed when the reaction was run in neat THF (entry 2). Exploration of other solvents and solvent mixtures (entries 3–9) revealed that MeCN/MeOH (1:1; entry 8) was optimal, providing the desired product in 77 % yield.

**Table 1:** Optimization of reaction conditions for generating **2 a**. 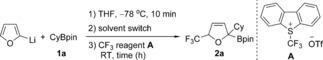

Entry^[a]^	Solvent for step 2	*t* [h]	Yield [%]^[b]^	**2 a**/**1 a** ^[c]^
1	MeCN	1	60	56:44
2	THF	1	trace	9:91
3	DMSO	1	56	56:44
4	DMF	1	57	63:37
5	MeOH	1	17	85:15
6	MeOH	12	45	86:14
7	MeCN/MeOH (3:1)	3	49	64:36
*8*	*MeCN/MeOH (1:1)*	*3*	*77*	*93:7*
9	MeCN/MeOH (1:3)	3	61	92:8

[a] Reactions were conducted using furan‐2‐yllithium (0.4 mmol), **1 a** (0.3 mmol) in 1.0 mL THF. After solvent switch (2 mL), CF_3_ reagent (0.4 mmol) was added. [b] Yield determined by ^19^F NMR analysis of the crude reaction mixture using Ph‐CF_3_ as an internal standard. [c] Ratio determined by GC‐MS analysis of the crude reaction mixture.

With an efficient process for generating intermediate **2 a** established, we investigated the oxidation of the boronic ester group (C‐Bpin to C‐OH) to allow rearomatization through dehydration. Unfortunately, standard oxidation conditions, such as H_2_O_2_/NaOH, Oxone, or NaBO_3_, led to a complex mixture of products devoid of the desired furan product. We reasoned that the cyclic hemiacetal formed under these basic conditions would be in equilibrium with the corresponding acyclic hemiketal, which, owing to the presence of the electron‐withdrawing CF_3_ group, may undergo side reactions. Fortunately, the use of iodine and K_2_CO_3_ was found to cleanly oxidize the intermediate to afford the desired furan product.

Using the optimized conditions, a variety of enantioenriched secondary boronic esters **1** were transformed into the corresponding trifluoromethyl‐substituted furan derivatives **3** in moderate to good yields (Scheme 2 A). Secondary boronic esters bearing sterically hindered alkyl groups and cyclopropyl, azide, silyl ether, *t*Bu ester, and benzyl functional groups underwent coupling with essentially complete enantiospecificity (**3 b**–**3 g**). Primary boronic esters **1 h** and **1 i** also coupled smoothly. In addition, hindered natural‐product‐derived boronic esters **1 j** (from menthol) and **1 k** (from cholesterol) coupled to give the corresponding furans in moderate yield and with complete diastereospecificity (ds). Furthermore, this three component coupling is not limited to the use of furan‐2‐yllithium, as demonstrated by the use of lithiated thiophene and *N*‐Boc‐pyrrole, which coupled to give 2,5‐disubstituted 5‐membered heterocycles **3 l**–**3 o**. Unfortunately, application of electron‐rich six‐membered aryllithium species, such as 3‐lithioanisole,^3^ failed to generate the corresponding trifluoromethylated intermediate.^12^

**Scheme 2:**
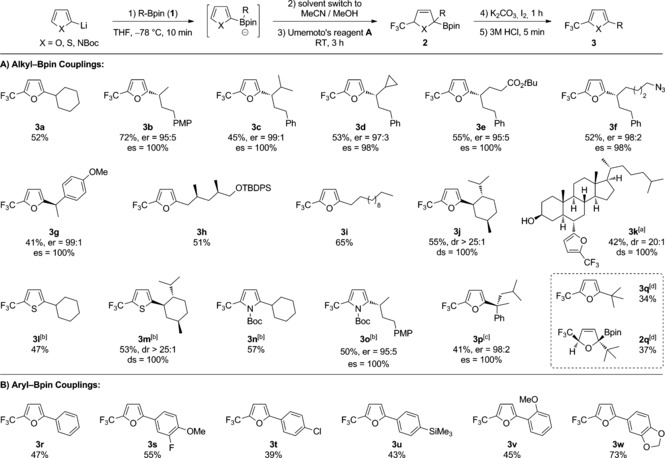
Scope of the three‐component trifluoromethylative coupling of boronic esters with furans, thiophenes, and pyrroles. Reactions were carried out with **1** (0.3 mmol), aryllithium (0.4 mmol), Umemoto's reagent **A** (0.4 mmol), K_2_CO_3_ (0.9 mmol) and I_2_ (0.4 mmol) in 2 mL MeCN/ MeOH (1:1). [a] TBS group on alcohol was removed under the reaction conditions. [b] DMF was used as solvent in place of MeCN/MeOH. [c] Conditions for oxidation: Cu(OAc)_2_ (0.6 mmol), TBAF (0.6 mmol), 4‐*tert*‐butylcatechol (1.2 mmol), 80 °C, 4 h. [d] Yield determined by ^19^F NMR analysis of the crude reaction mixture using Ph‐CF_3_ as an internal standard. PMP=*para*‐methoxyphenyl; TBDPS=*tert*‐butyldiphenylsilyl; TBS=*tert*‐butyldimethylsilyl; TBAF=tetra‐*n*‐butylammonium fluoride.

The coupling of tertiary boronic ester **1 p** proved to be more challenging, the increased steric hindrance imposing a negative effect on the final oxidation step. Specifically, only one of the two diastereomers of **2 p** was converted into furan product **3 p** (32 % yield from RBpin) under the standard reaction conditions. We therefore investigated alternative oxidants and found that Cu(OAc)_2_ was able to oxidize both diastereomers of **2 p** and gave the product **3 p** in an improved yield (41 %).^13^ The contrasting behavior of the two diastereomers warranted further investigation, which was conducted with the achiral *tert*‐butyl‐Bpin. As before, one diastereomer was rapidly oxidized to the desired product **3 q** leaving behind the unreactive diastereomer **2 q**, which was fully characterized. We believe that **2 q** is particularly slow to react with oxidants/electrophiles because both faces of the alkene are especially hindered: the top face by both the CF_3_ and Bpin groups and the bottom face by the tertiary alkyl group.

Because trifluoromethyl‐containing biaryl compounds remain to be attractive target molecules, we investigated the three‐component coupling reaction involving arylboronic esters. Pleasingly, a variety of arylboronic esters, including a sterically hindered *ortho*‐substituted substrate, coupled to give the corresponding furans **3 r**–**3 w** in moderate to good yields (Scheme 2 B).

In addition to electrophilic trifluoromethylation reagents, we found that other electrophiles can also be applied in the three‐component coupling reaction (Scheme 3). For example, the addition of the tropylium cation to a boronate complex led to an enantiospecific transformation into the desired 7‐furanyl cycloheptatriene derivative **4 a**, a member of a class of compounds that have recently found new applications in the generation of gold carbenes.^14^ Additionally, treatment of boronate complexes with 1,3‐benzodithiolylium tetrafluoroborate gave the desired adducts in good yields (**4 b** and **4 c**).

**Scheme 3:**
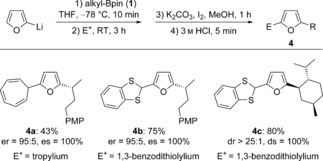
Alternative electrophiles. Reactions were carried out with **1** (0.3 mmol), furan‐2‐yllithium (0.4 mmol), electrophile (0.4 mmol), K_2_CO_3_ (0.9 mmol) and I_2_ (0.4 mmol) in 2 mL THF. PMP=*para*‐methoxyphenyl.

We were interested in shedding light on the mechanism of the three‐component trifluoromethylation reaction. Since both polar and radical mechanisms for electrophilic trifluoromethylation have been proposed previously, we used electron paramagnetic resonance (EPR) spectroscopy to identify whether the CF_3_ radical was being generated in our reaction. This was achieved by performing our standard trifluoromethylation reaction in the presence of two equivalents of the spin trap *N*‐*tert*‐butyl‐α‐phenylnitrone (PBN, **5**). The EPR spectrum of the resulting mixture shows the formation of CF_3_‐PBN spin trap **6** (Figure 1), demonstrating the generation of the trifluoromethyl radical under the reaction conditions.^15^ Control experiments confirmed that the CF_3_ radical was only formed in the presence of both the trifluoromethylating reagent **A** and the boronate complex.^12^ Furthermore, the yield of intermediate **2 a** formed in the presence of PBN was reduced to 9 %, suggesting that the CF_3_ radical reacts more rapidly with PBN than with boronate complex **I**.

**Figure 1:**
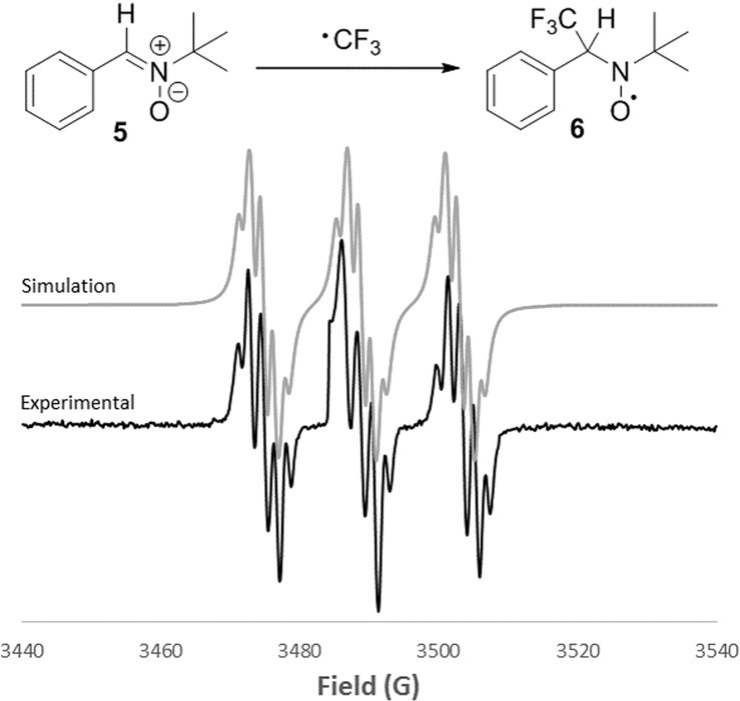
X‐band EPR spectrum obtained in the reaction of boronate complex derived from **1 a** with Umemoto's reagent **A** in the presence of PBN **5** (2 equiv) in DMF at 298 K. *g=*2.0044, *a*_N_=1.411 mT, *a*_H_=0.1664 mT, *a*_F_=0.1781 mT.

Based on these results, we propose the mechanism shown in Scheme 4. First, furan‐2‐yllithium reacts with the enantioenriched alkylboronic ester to form boronate complex **I**, which then undergoes single‐electron transfer (SET) with the electrophilic trifluoromethylating reagent to give the trifluoromethyl radical (initiation).^16^ This highly reactive electrophilic radical reacts with electron‐rich boronate complex **I** to give intermediate **VI**. Oxidation of intermediate **VI** by the trifluoromethylating reagent affords transient species **VII**, whilst also regenerating the trifluoromethyl radical (propagation). Species **VII** undergoes a 1,2‐metallate rearrangement to give boronic ester **VIII**. It is likely that oxidation of intermediate **VI** leads directly to boronic ester **VIII** but species **VII** is drawn for clarification.3b Under oxidative conditions (K_2_CO_3_, I_2_), rearomatization occurs to form the final three‐component‐coupled products with complete enantiospecificity. While the EPR studies suggest this SET mechanism is operative in the case of the trifluoromethylation‐mediated coupling reactions, further studies are required to determine whether the electrophiles shown in Scheme 3 proceed via a similar SET mechanism or an alternative two‐electron process.

**Scheme 4:**
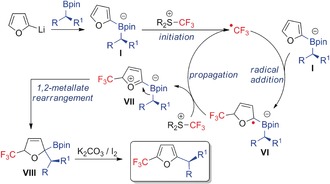
Proposed mechanism for CF_3_ radical‐induced three‐component coupling of boronic esters with furans.

In summary, we report a trifluoromethyl radical‐induced three‐component coupling of furans with enantioenriched secondary and tertiary alkyl and aryl boronic esters with essentially complete enantiospecificity. Mechanistic studies demonstrated that a radical pathway (SET initiation) is operative under the reaction conditions. In addition to the incorporation of the important trifluoromethyl group, other cationic electrophiles can also be applied, significantly expanding the methodology.

## Conflict of interest

The authors declare no conflict of interest.

## Supporting information

As a service to our authors and readers, this journal provides supporting information supplied by the authors. Such materials are peer reviewed and may be re‐organized for online delivery, but are not copy‐edited or typeset. Technical support issues arising from supporting information (other than missing files) should be addressed to the authors.

Supplementary
