# Supplementary material for: Enantiospecific Trifluoromethyl‐Radical‐Induced Three‐Component Coupling of Boronic Esters with Furans
Source: Angew Chem Int Ed Engl. 2017 Jan 18;56(7):1810–4. doi: 10.1002/anie.201611058 (PMC5499725; doi:10.1002/anie.201611058)

Angewandte Chemie
Eine Zeitschrift der Gesellschaft Deutscher Chemiker

Supporting Information

Enantiospecific Trifluoromethyl-Radical-Induced Three-Component Coupling of Boronic Esters with Furans

*Yahui Wang, Adam Noble, Christopher Sandford, and Varinder K. Aggarwal**

anie_201611058_sm_miscellaneous_information.pdf

Contents

1. General Experimental 2
2. Reaction Optimization 4
3. General Procedures........ 5
4. Product Characterization 6
5. Examples of Unreactive/Challenging Substrates 30
6. EPR Experiments 31
7. ^{1}H, ^{13}C and ^{19}F NMR spectra 35

1. General Experimental

1.1 Solvents and Reagents

All air and water-sensitive reactions were carried out in flame-dried glassware under a nitrogen atmosphere using standard Schlenk manifold techniques. Bulk solutions were evaporated under reduced pressure using a Büchi rotary evaporator. All solvents were commercially supplied or provided by the communal stills of the School of Chemistry, University of Bristol. Petroleum ether refers to the fraction collected between 40 – 60 °C. *n*BuLi was purchased from Acros. The molarity of organolithium solutions was determined by titration using *N*-benzyl benzamide as an indicator.[1] 5-(Trifluoromethyl)dibenzothiophenium trifluoromethanesulfonate (Umemoto's reagent, CAS:129946-88-9) and all other reagents were purchased from commercial sources and used as received. Cryogenic temperature was achieved using an acetone/CO_2 cold bath.

1.2 Chromatography and Spectroscopy

Flash column chromatography (FCC) was carried out using silica gel LC60A-40 (63 µm). All reactions were followed by thin-layer chromatography (TLC) when practical, using Merck Kieselgel 60 F_{254} fluorescent treated silica which was visualised under UV light or by staining with aqueous basic potassium permanganate or an ethanolic solution of phosphomolybdic acid.

^{1}H, ^{13}C and ^{19}F NMR spectra were recorded using Jeol ECS 300 MHz, Jeol ECS 400 MHz, Varian VNMR 400 MHz and Varian VNMR 500 MHz spectrometers. Chemical shifts (δ) are given in parts per million (ppm), and coupling constants (*J*) are given in Hertz (Hz). The ^{1}H NMR spectra are reported as follows: ppm (multiplicity, coupling constants, number of protons).

High resolution mass spectra (**HRMS**) were recorded on a VG Analytical Autospec by Electron Ionisation (EI) or on a Brüker Daltonics Apex IV by Electrospray Ionisation (ESI). **IR spectra** were recorded on a Perkin Elmer Spectrum One FT-IR as a thin film. Only selected absorption maxima (ν_{max}) are reported in wavenumbers (cm^{-1}). **Melting points** were recorded in degrees Celsius (°C), using a Kofler hot-stage microscope apparatus and are reported uncorrected. **Optical rotation** ($[\alpha]_D^T$) was measured on a

[1] A. F. Burchat, J. M. Chong, N. Nielsen, *J. Organomet. Chem.* **1997**, *542*, 281-283.

Bellingham and Stanley Ltd. ADP220 polarimeter and is quoted in (° ml)(g dm)$^{-1}$. **Chiral HPLC** was performed on a HP Agilent 1100 with a Chiralpak column and monitored by DAD (Diode Array Detector). **GC-MS** was performed on an Agilent 7820A using a HP-5MS UI column (30 m × 0.25 mm × 0.25 μm). Enantiospecificity: **Es** = 100 × (ee product) / (ee reactant).

1.3 Naming of compounds

Compound names are those generated by ChemBioDraw 13.0 software (PerkinElmer), following the IUPAC nomenclature.

1.4 Synthesis of Starting Materials

All of the boronic esters used in this paper are commercially available or have been reported previously and were prepared according to the literature:

1a[2], **1b**[3], **1c**[4], **1d**[4], **1e**[4], **1f**[4], **1g**[5], **1h**[6], **1i**[6], **1j**[7], **1k**[8], **1p**[8], **1q**[2], **1r**[2], **1s**[2], **1t**[2], **1u**[2].

[2] Purchased from Alfa Aesar and Aldrich.
[3] R. Larouche-Gauthier, T. G. Elford, V. K. Aggarwal, *J. Am. Chem. Soc.* **2011**, *133*, 16794-16797.
[4] C. Sandford, R. Rasappan, V. K. Aggarwal, *J. Am. Chem. Soc.* **2015**, *137*, 10100-10103.
[5] D. Noh, S. K. Yoon, J. Won, J. Y. Lee, J. Yun, *Chem. Asian J.* **2011**, *6*, 1967-1969.
[6] R. Rasappan, V. K. Aggarwal, *Nat. Chem.* **2014**, *6*, 810-814.
[7] H. Ito, K. Kubota, *Org. Lett.* **2012**, *14*, 890-893.
[8] A. Bonet, M. Odachowski, D. Leonori, S. Essafi, V. K. Aggarwal, *Nat. Chem.* **2014**, *6*, 584-589.

2. Reaction Optimization

To a stirred solution of furan (0.40 M in THF, 1.0 ml, 0.40 mmol) under N_2 at –78 °C was added *n*BuLi (1.6 M in hexane, 244 µL, 0.39 mmol) dropwise. The resulting solution was stirred for 1 h at r.t. (23 °C) before the addition of a solution of Cy-Bpin **1a** (0.30 mmol) in THF (0.5 mL) at –78 °C. The reaction was stirred for 10 min at –78 °C before removing the solvent under vacuum. Solvent (2 mL) was added at 23 °C followed by trifluoromethylating reagent[9] (0.40 mmol). The solution was stirred at T °C for 1 h. $PhCF_3$ (0.30 mmol) was added into this solution as an internal standard for ^{19}F NMR. A sample was removed and analysed by GC-MS.

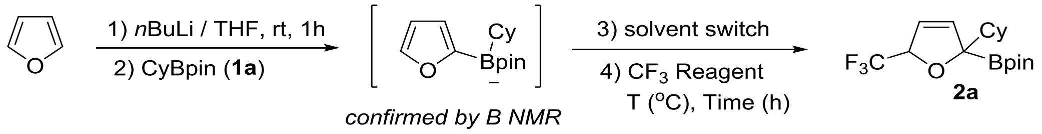

Entry	Solvent	CF_3 Reagent	T (°C)	Time (h)	Yield (%)^a	Product Ratio (1a : 2a)^b
1	THF	**A**	23	1	trace	91 : 9
2	CH_3CN	**A**	23	1	60	44 : 56
3	CH_3CN	**B**	23	1	48	48 : 52
4	CH_3CN	**C**	23	1	34	70 : 30
5	CH_3CN	**D**	23	1	trace	--
6	CH_3CN	**E**	23	1	7	77 : 23
7	DMSO	**F**	23	3	12	--
8	CH_3CN	**A**	–10	1	60	27 : 73
9	CH_3CN	**A**	60	1	64	34 : 66
10	MeOH	**A**	23	1	17	15 : 85
11	MeOH	**A**	23	12	45	14 : 86
12	DMSO	**A**	23	1	56	44 : 56
13	DMF	**A**	23	1	57	37 : 63
14	dioxane	**A**	23	1	8	87 : 13
15	CH_2Cl_2	**A**	23	1	43	44 : 56
16	$CHCl_3$	**A**	23	1	47	43 : 57
17	DMPU	**A**	23	1	40	46 : 54
18	acetone	**A**	23	1	trace	84 : 16
19	pyridine	**A**	23	1	trace	95 : 5
20	CH_3CN/MeOH (3 : 1)	**A**	23	3	49	36 : 64
**21**	**CH_3CN/MeOH (1 : 1)**	**A**	**23**	**3**	**77**	**7 : 93**
22	CH_3CN/MeOH (1 : 3)	**A**	23	3	61	8 : 92

^{a} Determined by ^{19}F NMR with $PhCF_3$ (0.30 mmol) as an internal standard. ^{b} Determined by GC-MS.

[9] For synthesis of trifluoromethyl sulfonium salts: a) T. Umemoto, S. Ishihara, *J. Am. Chem. Soc.* **1993**, *115*, 2156–2164; b) E. Magnier, J.-C. Blazejewski, M. Tordeux, C. Wakselman, *Angew. Chem. Int. Ed.* **2006**, *45*, 1279–1282; *Angew. Chem.* **2006**, *118*, 1301–1304; for CF_3I•2DMSO, see: c) F. Sladojevich, E. McNeill, J. Börgel, S.-L. Zheng, T. Ritter, *Angew. Chem. Int. Ed.* **2015**, *54*, 3712–3716; *Angew. Chem.* **2015**, *127*, 3783–3787.

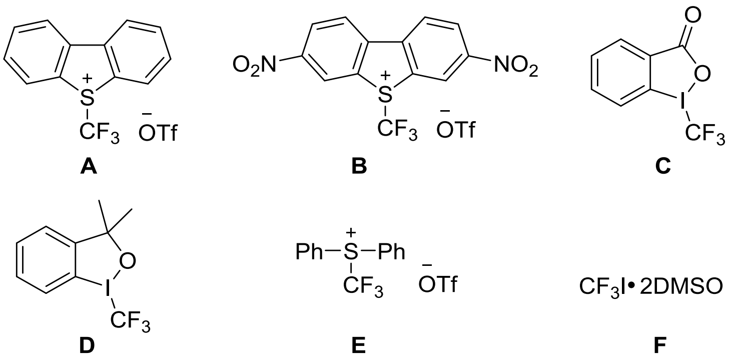

3. General Procedures

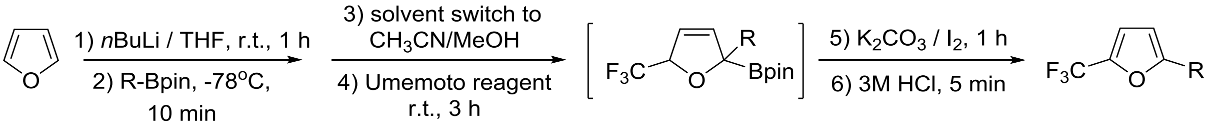

To a stirred solution of furan (0.40 M in THF, 1.0 ml, 0.40 mmol) under N_2 at –78 °C was added *n*BuLi (1.6 M in hexane, 244 µL, 0.390 mmol) dropwise. The resulting solution was stirred for 1 h at r.t. (23 °C) before the addition of a solution of boronic ester (0.30 mmol) in THF (0.5 mL) at –78 °C. The reaction was stirred for 10 min at –78 °C before removing the solvent under vacuum. MeOH (1 mL) and CH_3CN (1 mL) were added at 23 °C followed by Umemoto reagent (161 mg, 0.400 mmol). The solution was stirred at r.t. (23 °C) for 3 h before the sequential addition of K_2CO_3 (124 mg, 0.900 mmol) and I_2 (102 mg, 0.400 mmol). The resulting solution was stirred for 1 h at 23 °C before the addition of a solution of HCl (3 M in water, 1 mL) and stirring continued for a further 5 min. Water (10 mL) was added and the product extracted with Et_2O (2 × 15 mL). The organic phases were washed with sat. aq. $NaHCO_3$ then 20% aq. $Na_2S_2O_3$, dried over $MgSO_4$, filtered and concentrated *in vacuo*. The crude product purified by flash column chromatography on silica gel.

Note: The role of HCl prior to work up is to convert the methanol adduct, often observed after oxidation of the dihydrofuran intermediate, into the final product:

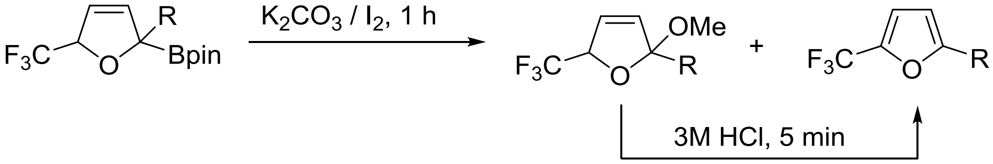

4. Product Characterization

2-Cyclohexyl-5-(trifluoromethyl)furan (3a)

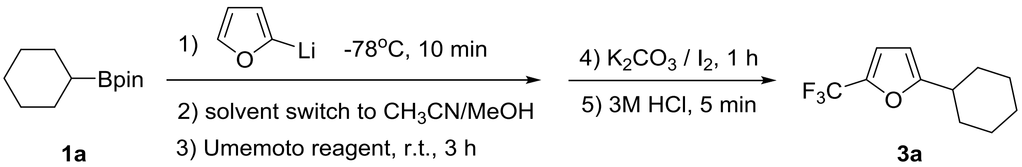

The starting boronic ester **1a** (63 mg, 0.30 mmol) was subjected to the General Procedure. The crude product was purified by column chromatography (petroleum ether) to afford the title compound as a colourless oil in 52% yield (34 mg).

R_f (petroleum ether): 0.6; **IR** (film) ν_{max}/cm^{-1}: 2931, 2857, 1553, 1320, 1170, 1123, 1090, 1017, 792; **^{1}H NMR** ($CDCl_3$, 300 MHz) δ (ppm): 6.66 (d, *J* = 3.4 Hz, 1H), 6.01 (d, *J* = 3.4 Hz, 1H), 2.70 – 2.60 (m, 1H), 2.10 – 1.99 (m, 2H), 1.88 – 1.68 (m, 3H), 1.45 – 1.22 (m, 5H); **^{13}C NMR** ($CDCl_3$, 75 MHz) δ (ppm): 163.9 (q, *J* = 1.4 Hz), 140.0 (q, *J* = 42.4 Hz), 119.4 (q, *J* = 266.3 Hz), 112.1 (q, *J* = 2.9 Hz), 103.9, 37.2, 31.2, 25.9, 25.8; **^{19}F NMR** ($CDCl_3$, 283 MHz) δ (ppm): –63.76; **HRMS** (EI) mass calculated for $[M]^+$ ($C_{11}H_{13}OF_3$) requires *m/z* 218.0918, found *m/z* 218.0920.

(*R*)-2-(4-(4-Methoxyphenyl)butan-2-yl)-5-(trifluoromethyl)furan (3b)

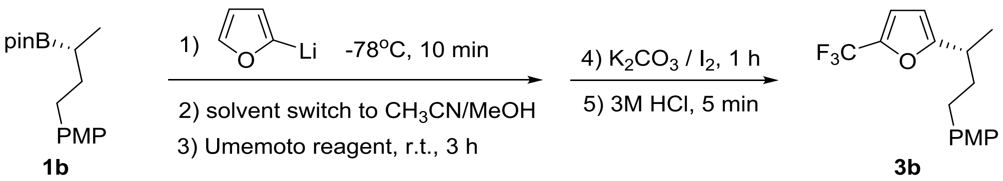

The starting boronic ester **1b** (95:5 er, 87 mg, 0.30 mmol) was subjected to the General Procedure. The crude product was purified by column chromatography (petroleum ether : ethyl acetate = 100:1) to afford the title compound as a colourless oil in 72% yield (64 mg) and 95:5 er (100% es).

$[\alpha]_D^{23}$ = –19 (*c* 1.0, $CHCl_3$); **R_f** (petroleum ether): 0.1; **IR** (film) ν_{max}/cm^{-1}: 2935, 1512, 1172, 1122, 1036, 795; **^{1}H NMR** ($CDCl_3$, 500 MHz) δ (ppm): 7.13 (d, *J* = 8.6 Hz, 2H), 6.88 (d, *J* = 8.6 Hz, 2H), 6.75 – 6.71 (m, 1H), 6.11 (dt, *J* = 3.4, 0.8 Hz, 1H), 3.83 (s, 3H), 2.91 (h, *J* = 7.0 Hz, 1H), 2.59 (t, *J* = 7.9 Hz, 2H), 2.13 –

2.00 (m, 1H), 1.95 – 1.81 (m, 1H), 1.34 (d, *J* = 7.0 Hz, 3H); **^{13}C NMR** ($CDCl_3$, 283 MHz) δ (ppm): 163.4 (q, *J* = 1.4 Hz), 157.8, 140.0 (q, *J* = 42.1 Hz), 133.8, 129.3, 119.0 (q, *J* = 266.2 Hz), 113.8, 112.1 (q, *J* = 2.8 Hz), 104.9, 55.2, 37.4, 32.7, 32.4, 18.9; **^{19}F NMR** ($CDCl_3$, 377 MHz) δ (ppm): –63.74; **HRMS** (ESI) mass calculated for $[M+Na]^+$ ($C_{16}H_{17}F_3NaO_2$) requires *m/z* 321.1073, found *m/z* 321.1063.

The er was determined by HPLC [chiralpak IB with guard, hexane/isopropanol 100/0, 0.5 mL/min, rt, λ = 210 nm, t (major) = 26.9 min, t (minor) = 29.0 min] to be 95:5 (100% es):

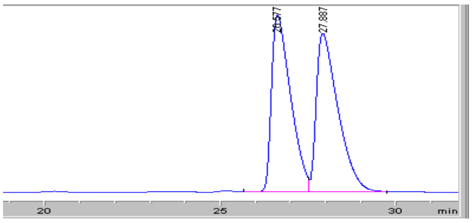

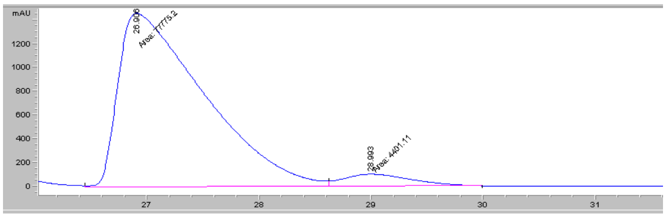

Peak #	RetTime [min]	Type	Width [min]	Area [mAU*s]	Height [mAU]	Area %
1	26.577	VV	0.5639	2.30053e4	610.17834	49.6224
2	27.887	VB	0.6312	2.33555e4	548.07001	50.3776

Peak #	RetTime [min]	Type	Width [min]	Area [mAU*s]	Height [mAU]	Area %
1	26.906	MF	0.8887	7.77752e4	1458.64539	94.6443
2	28.993	FM	0.7139	4401.10840	102.74625	5.3557

(*S*)-2-(4-Methyl-1-phenylpentan-3-yl)-5-(trifluoromethyl)furan (3c)

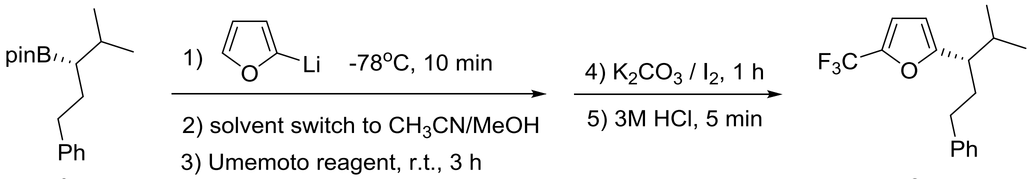

1c **3c**

The starting boronic ester **1c** (99:1 er, 86 mg, 0.30 mmol) was subjected to the General Procedure. The crude product was purified by preparative TLC (petroleum ether) to afford the title compound as a colourless oil in 45% yield (40 mg) and 99:1 er (100% es).

$[\alpha]_D^{23}$ = –21 (*c* 1.0, $CHCl_3$); **R_f** (petroleum ether): 0.5; **IR** (film) ν_{max}/cm^{-1}: 2961, 1548, 1322, 1172, 1125, 1017, 795; **^{1}H NMR** ($CDCl_3$, 300 MHz) δ (ppm): 7.29 (t, *J* = 7.1 Hz, 2H), 7.19 (t, *J* = 7.0 Hz, 1H), 7.13 (d, *J* = 7.1 Hz, 2H), 6.75 – 6.71 (m, 1H), 6.11 (d, *J* = 3.4 Hz, 1H), 2.60 – 2.43 (m, 3H), 2.05 – 1.85 (m, 3H), 0.91 (d, *J* = 6.9 Hz, 3H), 0.82 (d, *J* = 6.8 Hz, 3H); **^{13}C NMR** ($CDCl_3$, 75 MHz) δ (ppm): 161.0 (q, *J* = 4.4 Hz), 142.0, 140.5 (q, *J* = 41.6 Hz), 128.4, 128.4, 125.9, 119.4 (q, *J* = 265.8 Hz), 112.1 (q, *J* = 2.9 Hz), 107.4,

45.5, 34.0, 32.7, 32.0, 20.7, 19.6; **^{19}F NMR** ($CDCl_3$, 283 MHz) δ (ppm): –63.80; **HRMS** (EI) mass calculated for $[M]^+$ ($C_{17}H_{19}F_3O$) requires *m/z* 296.1388, found *m/z* 296.1390.

The er was determined by HPLC [chiralpak OD-H, hexane/isopropanol 100/0, 0.3 mL/min, rt, λ = 210 nm, t (minor) = 20.6 min, t (major) = 21.5 min] to be 99:1 (100% es):

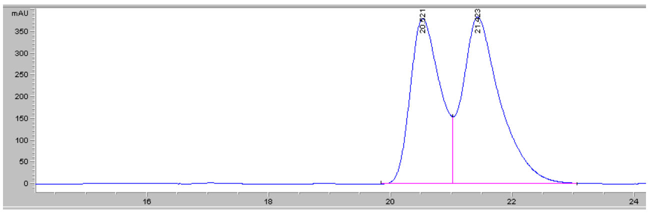

Peak #	RetTime [min]	Type	Width [min]	Area [mAU*s]	Height [mAU]	Area %
1	20.521	PV	0.5118	1.30435e4	380.58484	44.1186
2	21.423	VB	0.6162	1.65211e4	383.78320	55.8814

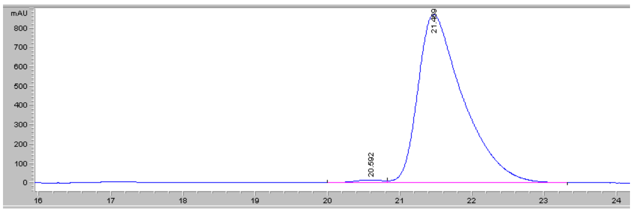

Peak #	RetTime [min]	Type	Width [min]	Area [mAU*s]	Height [mAU]	Area %
1	20.592	BV	0.3495	392.84015	14.07833	1.0256
2	21.469	VB	0.6313	3.79099e4	868.42969	98.9744

(*S*)-2-(1-Cyclopropyl-3-phenylpropyl)-5-(trifluoromethyl)furan (3d)

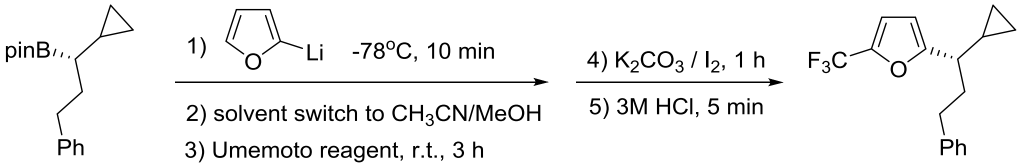

1d **3d**

The starting boronic ester **1d** (98:2 er, 57 mg, 0.20 mmol) was subjected to the General Procedure with **B** as the trifluoromethylating reagent. The crude product was purified by preparative TLC (petroleum ether) to afford the title compound as a colourless oil in 53% yield (31 mg) and 97:3 er (98% es).

$[\alpha]_D^{23}$ = +5 (*c* 1.0, $CHCl_3$); **R_f** (petroleum ether): 0.5; **IR** (film) ν_{max}/cm^{-1}: 3004, 1551, 1322, 1172, 1124, 1099, 1017, 796; **^{1}H NMR** ($CDCl_3$, 300 MHz) δ (ppm): 7.35 – 7.15 (m, 5H), 6.74 – 6.70 (m, 1H), 6.15 (d, *J* = 3.4 Hz, 1H), 2.70 – 2.61 (m, 2H), 2.21 – 2.00 (m, 3H), 1.05 – 0.96 (m, 1H), 0.65 – 0.46 (m, 2H), 0.23 – 0.14 (m, 2H); **^{13}C NMR** ($CDCl_3$, 75 MHz) δ (ppm): 161.9 (br), 142.0, 140.2 (q, *J* = 42.4 Hz), 128.5, 128.4, 125.9, 119.5 (q, *J* = 267.2 Hz), 112.2 (q, *J* = 2.8 Hz), 106.0, 43.5, 35.7, 33.4, 15.1, 5.0, 3.8; **^{19}F NMR** ($CDCl_3$, 283 MHz) δ (ppm): –63.76; **HRMS** (ESI) mass calculated for $[M+Na]^+$ ($C_{17}H_{17}F_3NaO$) requires *m/z* 317.1124, found *m/z* 317.1115.

The er was determined by HPLC [chiralpak IB with guard, hexane/isopropanol 99.9/0.1, 0.5 mL/min, rt, λ = 210 nm, t (minor) = 13.3 min, t (major) = 13.9 min] to be 97:3 (98% es):

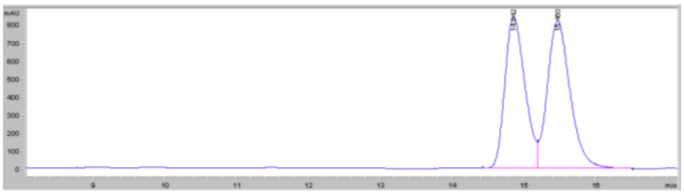

Peak #	RetTime [min]	Type	Width [min]	Area [mAU*s]	Height [mAU]	Area %
1	14.842	VV	0.3017	1.62810e4	841.48639	47.4451
2	15.460	VB	0.3312	1.80345e4	817.93909	52.5549

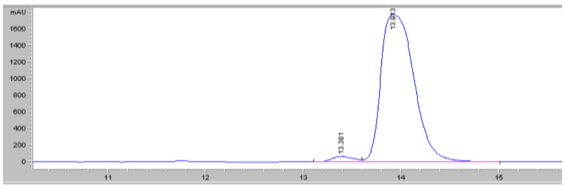

Peak #	RetTime [min]	Type	Width [min]	Area [mAU*s]	Height [mAU]	Area %
1	13.381	VV	0.2525	1081.56433	66.94559	2.5134
2	13.913	VV	0.3214	4.19497e4	1777.34692	97.4866

tert-Butyl (*S*)-6-phenyl-4-(5-(trifluoromethyl)furan-2-yl)hexanoate (3e)

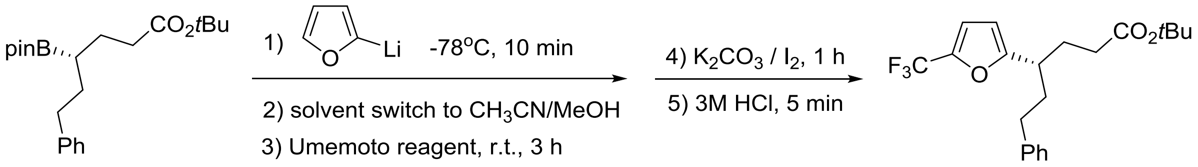

1e **3e**

The starting boronic ester **1e** (95:5 er, 75 mg, 0.20 mmol) was subjected to the General Procedure. The crude product was purified by preparative TLC (petroleum ether : ethyl acetate = 20:1) to afford the title compound as a colourless oil in 55% yield (42 mg) and 95:5 er (100% es).

[α]$_D$^{23} = +1 (*c* 1.0, $CHCl_3$); **R$_f$** (petroleum ether : ethyl acetate = 20:1): 0.3; **IR** (film) ν_{max}/cm^{-1}: 2979, 1726, 1320, 1171, 1126, 1102, 797; **^{1}H NMR** ($CDCl_3$, 300 MHz) δ (ppm): 7.34 – 7.08 (m, 5H), 6.73 – 6.70 (m, 1H), 6.12 (d, *J* = 3.4 Hz, 1H), 2.85 – 2.76 (m, 1H), 2.61 – 2.47 (m, 2H), 2.21 – 1.85 (m, 6H), 1.42 (s, 9H); **^{13}C NMR** ($CDCl_3$, 75 MHz) δ (ppm): 172.5, 160.9 (q, *J* = 1.4 Hz), 141.6, 140.6 (q, *J* = 42.3 Hz), 128.5, 128.4, 126.0, 121.1 (q, *J* = 266.5 Hz), 112.2 (q, *J* = 2.8 Hz), 107.0, 80.4, 38.0, 35.6, 33.5, 33.1, 29.3, 28.1; **^{19}F NMR** ($CDCl_3$, 283 MHz) δ (ppm): –63.76; **HRMS** (ESI) mass calculated for $[M+Na]^+$ ($C_{21}H_{25}F_3NaO_3$) requires *m/z* 405.1648, found *m/z* 405.1633.

The er was determined by HPLC [chiralpak IB with guard, hexane/isopropanol 99/1, 1 mL/min, rt, λ = 273 nm, t (minor) = 4.6 min, t (major) = 5.0 min] to be 95:5 (100% es):

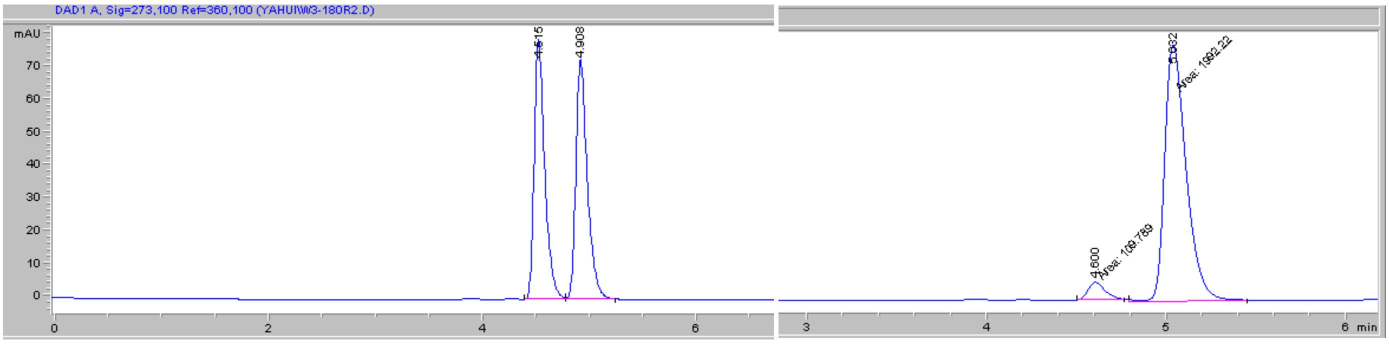

Peak #	RetTime [min]	Type	Width [min]	Area [mAU*s]	Height [mAU]	Area %
1	4.515	BV	0.1022	544.84509	79.24416	50.0114
2	4.908	VB	0.1110	544.59625	73.04176	49.9886

Peak #	RetTime [min]	Type	Width [min]	Area [mAU*s]	Height [mAU]	Area %
1	4.600	MM	0.1125	109.78866	16.26583	5.2230
2	5.032	MM	0.1417	1992.21997	234.40289	94.7770

(*R*)-2-(7-Azido-1-phenylheptan-3-yl)-5-(trifluoromethyl)furan (3f)

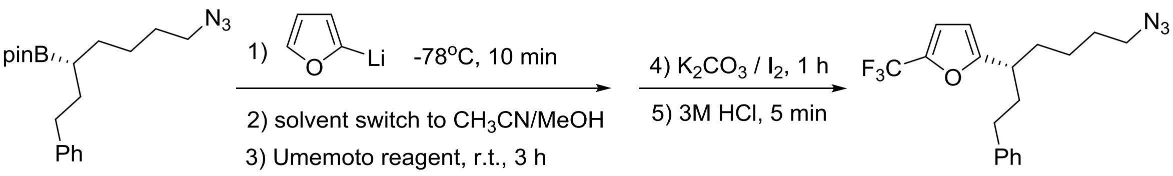

1f **3f**

The starting boronic ester **1f** (99:1 er, 103 mg, 0.300 mmol) was subjected to the General Procedure. The crude product was purified by preparative TLC (petroleum ether : ethyl acetate = 50:1) to afford the title compound as a colourless oil in 52% yield (55 mg) and 98:2 er (98% es).

$[\alpha]_D^{23}$ = –6 (*c* 1.0, $CHCl_3$); **R_f** (petroleum ether): 0.1; **IR** (film) ν_{max}/cm^{-1}: 2938, 2093, 1321, 1172, 1124, 1017, 797; **^{1}H NMR** ($CDCl_3$, 300 MHz) δ (ppm): 7.31 – 7.10 (m, 5H), 6.72 – 6.69 (m, 1H), 6.11 (d, *J* = 3.4 Hz, 1H), 3.23 (t, *J* = 7.0 Hz, 2H), 2.80 – 2.71 (m, 1H), 2.61 – 2.45 (m, 2H), 2.04 – 1.88 (m, 2H), 1.71 – 1.52 (m, 4H), 1.30 – 1.22 (m, 2H); **^{13}C NMR** ($CDCl_3$, 75 MHz) δ (ppm): 161.5 (q, *J* = 1.4 Hz), 141.7, 140.3 (q, *J* = 42.1 Hz), 128.4, 128.4, 126.0, 119.3 (q, *J* = 265.9 Hz), 112.2 (q, *J* = 2.8 Hz), 106.6, 51.3, 38.6, 35.6, 33.6, 33.5, 28.8, 24.4; **^{19}F NMR** ($CDCl_3$, 283 MHz) δ (ppm): –63.76; **HRMS** (ESI) mass calculated for $[M+Na]^+$ ($C_{18}H_{20}F_3N_3NaO$) requires *m/z* 374.1451, found *m/z* 374.1435.

The er was determined by HPLC [chiralpak IB with guard, hexane/isopropanol 99.9/0.1, 0.8 mL/min, rt, λ = 210 nm, t (minor) = 16.0 min, t (major) = 18.3 min] to be 98:2 (98% es):

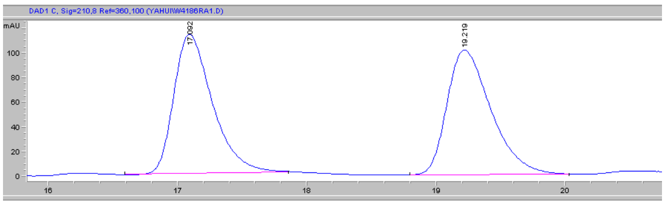

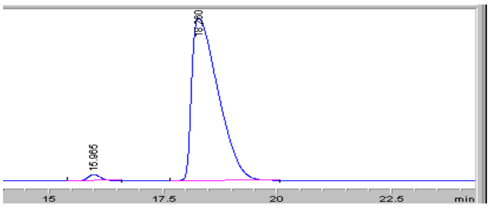

Peak #	RetTime [min]	Type	Width [min]	Area [mAU*s]	Height [mAU]	Area %
1	17.092	VB	0.3142	2365.29834	113.01809	49.6753
2	19.219	BB	0.3670	2396.22363	100.91299	50.3247

Peak #	RetTime [min]	Type	Width [min]	Area [mAU*s]	Height [mAU]	Area %
1	15.965	BB	0.2822	726.54822	38.48702	1.7444
2	18.260	PB	0.5973	4.09235e4	1017.43933	98.2556

(*R*)-2-(1-(4-Methoxyphenyl)ethyl)-5-(trifluoromethyl)furan (3g)

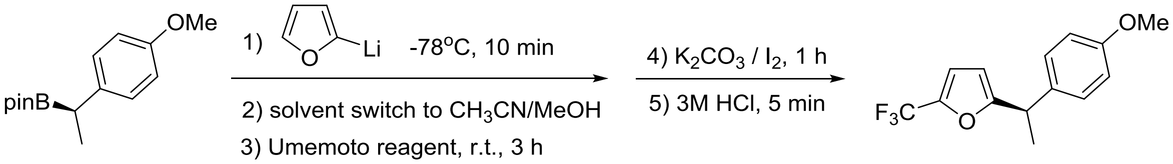

1g **3g**

The starting boronic ester **1g** (99:1 er, 79 mg, 0.30 mmol) was subjected to the General Procedure. The crude product was purified by preparative TLC (petroleum ether : ethyl acetate = 100:1) to afford the title compound as a colourless oil in 41% yield (33 mg) and 99:1 er (100% es).

$[\alpha]_D^{23}$ = +2 (*c* 1.0, $CHCl_3$); **R_f** (petroleum ether): 0.1; **IR** (film) ν_{max}/cm^{-1}: 2936, 1512, 1320, 1172, 1102, 1034, 831, 797; **^{1}H NMR** ($CDCl_3$, 300 MHz) δ (ppm): 7.15 (d, *J* = 8.4 Hz, 2H), 6.86 (d, *J* = 8.4 Hz, 2H), 6.68 – 6.65 (m, 1H), 6.02 (d, *J* = 3.4 Hz, 1H), 4.11 (q, *J* = 7.2 Hz, 1H), 3.79 (s, 3H), 1.60 (d, *J* = 7.2 Hz, 3H); **^{13}C NMR** ($CDCl_3$, 75 MHz) δ (ppm): 162.4 (q, *J* = 1.4 Hz), 158.4, 140.4 (q, *J* = 42.3 Hz), 134.8, 128.2, 122.6 (q, *J* = 270.9 Hz), 113.9, 112.1 (q, *J* = 2.8 Hz), 105.8, 55.2, 38.4, 20.3; **^{19}F NMR** ($CDCl_3$, 283 MHz) δ (ppm): –63.75; **HRMS** (ESI) mass calculated for $[M+Na]^+$ ($C_{14}H_{13}F_3NaO_2$) requires *m/z* 293.0760, found *m/z* 293.0750.

The er was determined by HPLC [chiralpak IB with guard, hexane/isopropanol 100/0, 0.5 mL/min, rt, λ = 210 nm, t (major) = 19.3 min, t (minor) = 21.3 min] to be 99:1 (100% es):

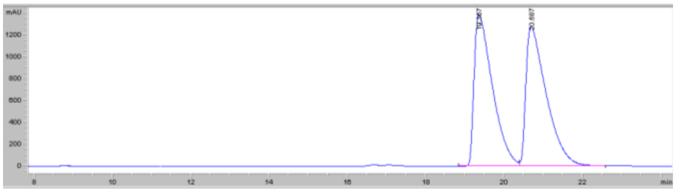

Peak #	RetTime [min]	Type	Width [min]	Area [mAU*s]	Height [mAU]	Area %
1	19.357	BV	0.4854	4.50260e4	1384.64807	49.4452
2	20.687	VB	0.5180	4.60365e4	1272.70239	50.5548

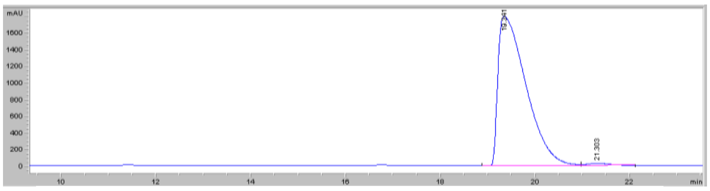

Peak #	RetTime [min]	Type	Width [min]	Area [mAU*s]	Height [mAU]	Area %
1	19.341	BV	0.5260	7.35414e4	1787.55017	98.8669
2	21.303	VB	0.4416	842.88379	26.75174	1.1331

tert-Butyl(((2*R*,4*S*)-2,4-dimethyl-5-(5-(trifluoromethyl)furan-2-yl)pentyl)oxy)diphenylsilane (3h)

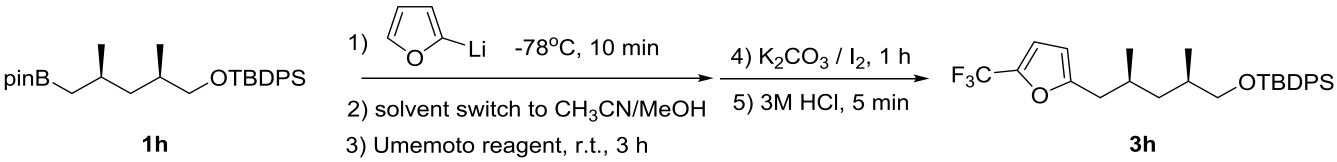

The starting boronic ester **1h** (>95:5 dr, 144 mg, 0.300 mmol) was subjected to the General Procedure. The crude product was purified by preparative TLC (petroleum ether) to afford the title compound as a colourless oil in 51% yield (75 mg) and >95:5 dr (100% ds).

$[\alpha]_D^{23}$ = +11 (*c* 3.0, $CHCl_3$); **R_f** (petroleum ether): 0.3; **IR** (film) ν_{max}/cm^{-1}: 2958, 1556, 1323, 1174, 1127, 1110, 1015, 792; **^{1}H NMR** ($CDCl_3$, 400 MHz) δ (ppm): 7.75 – 7.65 (m, 4H), 7.48 – 7.36 (m, 6H), 6.67 (dq, *J* = 2.5, 1.2 Hz, 1H), 6.04 (dq, *J* = 3.4, 0.7 Hz, 1H), 3.53 (dd, *J* = 9.8, 5.4 Hz, 1H), 3.46 (dd, *J* = 9.9, 6.2 Hz, 1H), 2.66 (dd, *J* = 15.0, 5.2 Hz, 1H), 2.40 (dd, *J* = 14.9, 8.3 Hz, 1H), 1.93 (dqd, *J* = 8.4, 6.9, 5.2 Hz, 1H), 1.78 (dq, *J* = 12.9, 6.6 Hz, 1H), 1.46 (dt, *J* = 13.6, 6.8 Hz, 1H), 1.08 (s, 9H), 1.06 – 0.99 (m, 1H), 0.98 (d, *J* = 6.7 Hz, 3H), 0.88 (d, *J* = 6.7 Hz, 3H); **^{13}C NMR** ($CDCl_3$, 75 MHz) δ (ppm): 158.6 (q, *J* = 1.4 Hz), 140.2 (q, *J* = 42.1 Hz), 135.7, 135.7, 134.1, 134.0, 129.6, 129.6, 127.7, 127.7, 119.3 (q, *J* = 264.4 Hz), 112.3 (q, *J* = 2.9 Hz), 107.1, 68.8, 40.8, 35.2, 33.3, 30.0, 26.9, 20.3, 19.4, 17.7; **^{19}F NMR** ($CDCl_3$, 283 MHz) δ (ppm): –63.71; **HRMS** (EI) mass calculated for $[M–C_4H_9]^+$ ($C_{24}H_{26}F_3SiO_2$) requires *m/z* 431.1654, found *m/z* 431.1658.

2-Dodecyl-5-(trifluoromethyl)furan (3i)

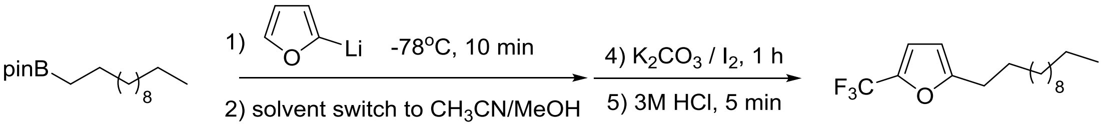

1i **3i**

The starting boronic ester **1i** (89 mg, 0.30 mmol) was subjected to the General Procedure. The crude product was purified by column chromatography (petroleum ether) to afford the title compound as a colourless oil in 65% yield (59 mg).

R$_f$ (petroleum ether): 0.7; **IR** (film) ν_{max}/cm^{-1}: 2924, 2854, 1558, 1322, 1173, 1128, 791; **^{1}H NMR** ($CDCl_3$, 300 MHz) δ (ppm): 6.66 (dt, *J* = 3.3, 1.3 Hz, 1H), 6.04 (dd, *J* = 3.3, 1.0 Hz, 1H), 2.64 (t, *J* = 7.6 Hz, 2H), 1.75 – 1.56 (m, 2H), 1.40 – 1.21 (m, 18H), 0.98 – 0.80 (m, 3H); **^{13}C NMR** ($CDCl_3$, 75 MHz) δ (ppm): 159.7 (q, *J* = 1.4 Hz), 140.2 (q, *J* = 42.6 Hz), 119.3 (q, *J* = 267.6 Hz), 112.2 (q, *J* = 2.8 Hz), 105.7, 32.0, 29.7, 29.7, 29.6, 29.5, 29.4, 29.3, 29.1, 27.9, 27.7, 22.7, 14.1; **^{19}F NMR** ($CDCl_3$, 283 MHz) δ (ppm): –63.85; **HRMS** (EI) mass calculated for $[M]^+$ ($C_{17}H_{27}F_3O$) requires *m/z* 304.2014, found *m/z* 304.2019.

2-((1*R*,2*S*,5*R*)-2-Isopropyl-5-methylcyclohexyl)-5-(trifluoromethyl)furan (3j)

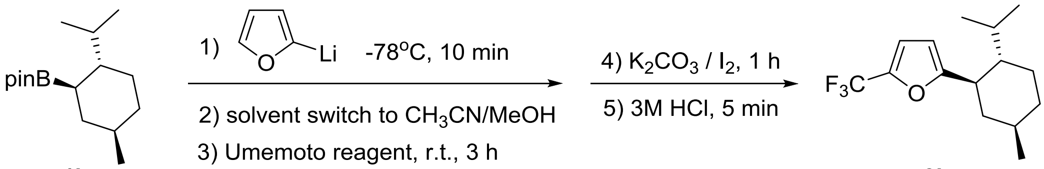

1j **3j**

The starting boronic ester **1j** (>95:5 dr, 80 mg, 0.30 mmol) was subjected to the General Procedure. The crude product was purified by column chromatography (petroleum ether) to afford the title compound as a colourless oil in 51% yield (42 mg) and >95:5 dr (100% ds).

[α]$_D$^{23} = –37 (*c* 1.0, $CHCl_3$); **R$_f$** (petroleum ether): 0.6; **IR** (film) ν_{max}/cm^{-1}: 2956, 1316, 1172, 1126, 1100, 1016, 792; **^{1}H NMR** ($CDCl_3$, 300 MHz) δ (ppm): 6.65 (dt, *J* = 3.4, 1.2 Hz, 1H), 6.02 (dt, *J* = 3.4, 0.9 Hz, 1H), 2.61 (td, *J* = 11.6, 3.4 Hz, 1H), 1.90 – 1.65 (m, 3H), 1.53 – 1.34 (m, 3H), 1.33 – 1.22 (m, 1H), 1.19 –

0.94 (m, 2H), 0.91 (dd, *J* = 6.4, 0.8 Hz, 3H), 0.84 (dd, *J* = 6.9, 0.8 Hz, 3H), 0.71 (dd, *J* = 6.9, 0.8 Hz, 3H); **^{13}C NMR** ($CDCl_3$, 75 MHz) δ (ppm): 162.9 (q, *J* = 1.4 Hz), 139.7 (q, *J* = 41.1 Hz), 119.4 (q, *J* = 267.8 Hz), 112.1 (q, *J* = 2.9 Hz), 105.3, 46.6, 41.6, 41.0, 34.8, 32.8, 28.5, 24.8, 22.3, 21.1, 15.8; **^{19}F NMR** ($CDCl_3$, 283 MHz) δ (ppm): –63.73; **HRMS** (EI) mass calculated for $[M]^+$ ($C_{15}H_{21}F_3O$) requires *m/z* 274.1545, found *m/z* 274.1542.

(3*S*,5*R*,6*S*,8*S*,9*S*,10*R*,13*R*,14*S*,17*R*)-10,13-Dimethyl-17-((*R*)-6-methylheptan-2-yl)-6-(5-(trifluoromethyl)furan-2-yl)hexadecahydro-1*H*-cyclopenta[*a*]phenanthren-3-ol (3k)

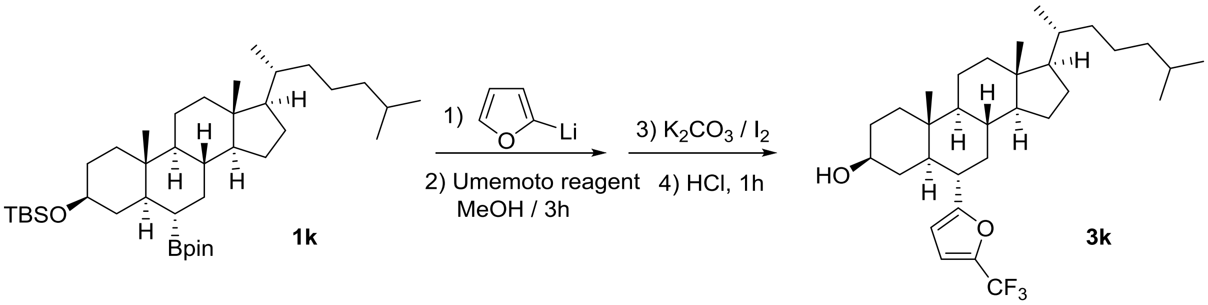

The title compound was synthesized with a modified General Procedure as shown below:

To a stirred solution of furan (0.40 M in THF, 1.0 ml, 0.40 mmol) under N_2 at –78 °C was added *n*BuLi (1.6 M in hexane, 244 µL, 0.390 mmol) dropwise. The resulting solution was stirred for 1 h at 23 °C before the addition of a solution of starting boronic ester **1k** (95:5 dr, 189 mg, 0.300 mmol) in THF (0.5 mL) at –78 °C. The reaction was stirred for 10 min at –78 °C before removing the solvent under vacuum. THF (0.5 mL) and MeOH (1 mL) were added at 23 °C followed by Umemoto reagent (161 mg, 0.400 mmol). The solution was stirred at 23 °C for 3 h before the sequential addition of K_2CO_3 (124 mg, 0.900 mmol) and I_2 (102 mg, 0.400 mmol). The resulting solution was stirred for 1 h at 23 °C before the addition of a solution of HCl (3 M in water, 1 mL) and stirring continued for a further 1 h. Water (10 mL) was added and the product extracted with Et_2O (2 × 15 mL). The organic phases were washed with sat. aq. $NaHCO_3$ then 20% aq. $Na_2S_2O_3$, dried over $MgSO_4$, filtered and concentrated *in vacuo*. The crude product purified by column chromatography (petroleum ether : ethyl acetate = 10:1) to afford the title compound as a white solid in 42% yield (66 mg) and 95:5 dr (100% ds).

[α]D²³ = +37 (*c* 1.0, $CHCl_3$); **M.p.**: 131–134 °C; **R_f** (petroleum ether : ethyl acetate = 3:1): 0.5; **IR** (film) ν_{max}/cm^{-1}: 3351(br), 2932, 1556, 1324, 1172, 1128, 1099, 733; **^1H NMR** ($CDCl_3$, 300 MHz) δ (ppm): 6.64 (dt, *J* = 3.1, 1.5 Hz, 1H), 6.02 (d, *J* = 3.3Hz, 1H), 3.48 (tt, *J* = 10.4, 4.5 Hz, 1H), 2.71 – 2.55 (m, 1H), 1.98 (dd, *J* = 9.7, 6.3 Hz, 1H), 1.85 – 1.76 (m, 4H), 1.52 – 0.80 (m, 37H), 0.66 (s, 3H); **^{13}C NMR** ($CDCl_3$, 75 MHz) δ (ppm): 162.3 (q, *J* = 1.4 Hz), 140.0 (q, *J* = 41.8 Hz), 119.2 (q, *J* = 266.0 Hz), 112.2 (q, *J* = 2.8 Hz), 105.9, 71.1, 56.3, 56.1, 53.8, 48.3, 42.6, 39.9, 39.5, 38.3, 37.9, 36.9, 36.2, 35.9, 35.8, 35.1, 34.8, 31.1, 28.3, 28.0, 24.2, 23.9, 22.8, 22.6, 21.3, 18.7, 12.9, 12.1; **^{19}F NMR** ($CDCl_3$, 283 MHz) δ (ppm): –63.57; **HRMS** (ESI) mass calculated for $[M+Na]^+$ ($C_{32}H_{49}F_3NaO_2$) requires *m/z* 545.3577, found *m/z* 545.3571.

2-Cyclohexyl-5-(trifluoromethyl)thiophene (3l)

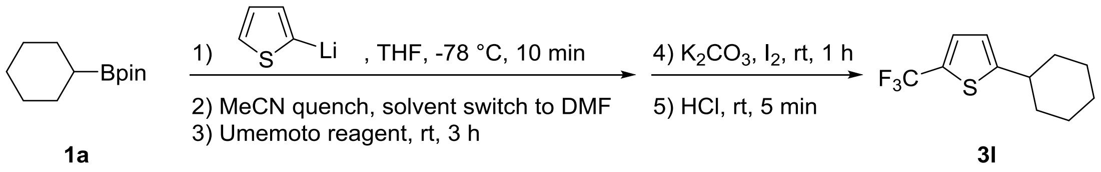

The title compound was synthesized with a modified General Procedure as shown below:

To a stirred solution of thiophene (0.40 M in THF, 1.0 mL, 0.40 mmol) under N_2 at –78 °C was added *n*BuLi (1.6 M in hexanes, 0.244 mL, 0.390 mmol) dropwise. The resulting solution was stirred for 1 h at 23 ºC before the addition of a solution of cyclohexylboronic acid pinacol ester **1a** (63 mg, 0.30 mmol) in THF (0.5 mL) at –78 °C. The reaction was stirred for 10 min at –78 °C, at which point MeCN (0.5 mL) was added and the solvent was then removed *in vacuo*. DMF (2.0 mL) was added at rt, followed by Umemoto reagent (161 mg, 0.40 mmol). The solution was stirred at 23 ºC for 3 h before the sequential addition of MeOH (2.0 mL), K_2CO_3 (124 mg, 0.900 mmol) and I_2 (102 mg, 0.400 mmol). The resulting solution was stirred for 1 h at 23 ºC before the addition of a solution of HCl (3.0 M in H_2O, 1.0 mL) and stirring continued for a further 5 min, at which point Et_2O/H_2O (1:1 v/v, 8 mL) and $PhCF_3$ (36.8 µL, 0.300 mmol) were added. A crude ^{19}F NMR yield (44%) was obtained by sampling the organic phase. To the mixture was added Et_2O/H_2O (1:1 v/v, 20 mL). The aqueous phase was extracted with Et_2O (2 × 10 mL), the combined organic phases washed with sat. aq. $NaHCO_3$ (20 mL) then 20% aq. $Na_2S_2O_3$ (20 mL), dried over $MgSO_4$,

filtered and concentrated *in vacuo*. The crude product was purified by column chromatography (pentane : Et_2O = 100:0 to 99:1) to afford the title compound as a colourless oil in 47% yield (33 mg).

R_f (90/10 pentane/Et_2O): 0.7; **IR** (film) ν_{max}/cm^{-1}: 2928, 1477, 1298, 1115, 1057, 1003, 807; **^{1}H NMR** ($CDCl_3$, 500 MHz) δ (ppm): 7.28 (m, 1H), 6.77 (m, 1H), 2.84 (m, 1H), 2.08 (m, 2H), 1.86 (m, 2H), 1.77 (m, 1H), 1.53 – 1.36 (m, 4H), 1.28 (m, 1H); **^{13}C NMR** ($CDCl_3$, 126 MHz) δ (ppm): 156.7, 128.2 (q, *J* = 4.0 Hz), 127.7 (q, *J* = 38.0 Hz), 122.7 (q, *J* = 268.0 Hz), 121.8, 39.5, 35.3, 26.3, 25.8; **^{19}F NMR** ($CDCl_3$, 470 MHz) δ (ppm): –55.0; **HRMS** (EI) mass calculated for $[M]^+$ ($C_{11}H_{13}F_3S^+$) requires *m/z* 234.0685, found *m/z* 234.0696.

2-((1*R*,2*S*,5*R*)-2-Isopropyl-5-methylcyclohexyl)-5-(trifluoromethyl)thiophene (3m)

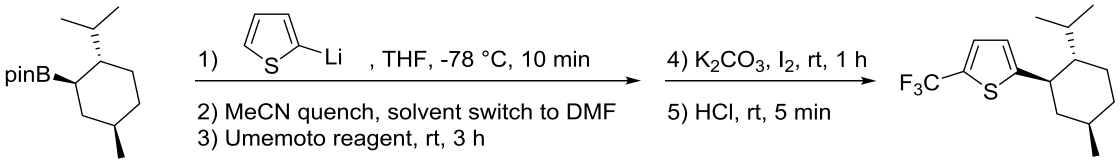

The title compound was synthesized with a modified General Procedure as shown below:

To a stirred solution of thiophene (0.40 M in THF, 1.0 mL, 0.40 mmol) under N_2 at –78 °C was added *n*BuLi (1.6 M in hexanes, 0.244 mL, 0.390 mmol) dropwise. The resulting solution was stirred for 1 h at 23 °C before the addition of a solution of boronic ester **1j** (>95:5 dr, 80 mg, 0.30 mmol) in THF (0.5 mL) at –78 °C. The reaction was stirred for 10 min at –78 °C, at which point MeCN (0.5 mL) was added and the solvent was then removed *in vacuo*. DMF (2.0 mL) was added at 23 °C followed by Umemoto reagent (161 mg, 0.40 mmol). The solution was stirred at 23 °C for 3 h before the sequential addition of MeOH (2.0 mL), K_2CO_3 (124 mg, 0.900 mmol) and I_2 (102 mg, 0.400 mmol). The resulting solution was stirred for 1 h at 23 °C before the addition of a solution of HCl (3.0 M in H_2O, 1.0 mL) and stirring continued for a further 5 min, at which point Et_2O/H_2O (1:1 v/v, 8 mL) and $PhCF_3$ (36.8 µL, 0.300 mmol) were added. A crude ^{19}F NMR yield (64%, >95:5 dr, 100% ds) was obtained by sampling the organic phase. To the mixture was added Et_2O/H_2O (1:1 v/v, 20 mL). The aqueous phase was extracted with Et_2O (2 × 10 mL), the

combined organic phases washed with sat. aq. $NaHCO_3$ (20 mL) then 20% aq. $Na_2S_2O_3$ (20 mL), dried over $MgSO_4$, filtered and concentrated *in vacuo*. The crude product was purified by column chromatography (pentane : Et_2O = 100:0 to 99:1) to afford the title compound as a colourless oil in 53% yield (46 mg) and >95:5 dr (100% ds).

$[\alpha]_D^{23}$ = –37 (*c* 0.77, $CHCl_3$); **R_f** (90/10 pentane/Et_2O): 0.7; **IR** (film) ν_{max}/cm^{-1}: 2957, 2920, 1479, 1297, 1151, 1118, 1054, 999, 809; **^{1}H NMR** ($CDCl_3$, 500 MHz) δ (ppm): 7.27 (dq, *J* = 3.5, 1.0 Hz, 1H), 6.75 (m, 1H), 2.81 (td, *J* = 11.5, 3.5 Hz, 1H), 1.98 (dtd, *J* = 13.0, 3.5, 2.0 Hz, 1H), 1.84 (ddq, *J* = 13.0, 3.5, 3.0 Hz, 1H), 1.78 (dq, *J* = 13.0, 3.0 Hz, 1H), 1.60 (heptd, *J* = 7.0, 3.0 Hz, 1H), 1.50 (m, 1H), 1.36 (tt, *J* = 11.5, 3.0 Hz, 1H), 1.20 (dt, *J* = 13.0, 11.5 Hz, 1H), 1.16 (dtd, *J* = 13.0, 11.5, 3.0 Hz, 1H), 1.04 (tdd, *J* = 13.0, 11.5, 3.0 Hz, 1H), 0.94 (d, *J* = 6.5 Hz, 3H), 0.87 (d, *J* = 7.0 Hz, 3H), 0.75 (d, *J* = 7.0 Hz, 3H); **^{13}C NMR** ($CDCl_3$, 126 MHz) δ (ppm): 155.4, 128.1 (q, *J* = 4.0 Hz), 127.9 (q, *J* = 38.0 Hz), 123.0, 122.7 (q, *J* = 268.0 Hz), 49.7, 46.4, 43.3, 34.9, 33.1, 27.5, 24.4, 22.3, 21.4, 15.2; **^{19}F NMR** (283 MHz, $CDCl_3$) δ (ppm): –54.9; **HRMS** (EI) mass calculated for $[M]^+$ ($C_{15}H_{21}F_3S^+$) requires *m/z* 290.1311, found *m/z* 290.1315.

***tert*-Butyl 2-cyclohexyl-5-(trifluoromethyl)-1*H*-pyrrole-1-carboxylate (3n)**

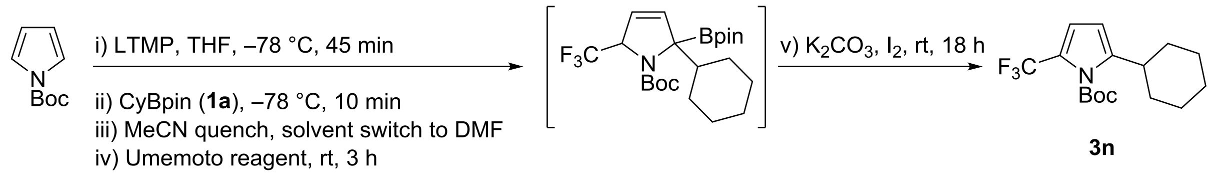

The title compound was synthesized with a modified General Procedure as shown below:

LTMP was freshly prepared by the dropwise addition of *n*BuLi (1.6 M in hexanes, 0.272 mL, 0.435 mmol) to 2,2,6,6-tetramethylpiperidine (0.435 M in THF, 1.00 mL, 0.435 mmol) at –78 °C. The solution was stirred at –78 °C for 5 min, then warmed to 23 ºC for 5 min, before cooling to –78 °C. *N*-Boc-pyrrole (75 µL, 0.45 mmol) was added, and the solution stirred for 45 min at –78 °C[10] before the addition of a solution of cyclohexylboronic acid pinacol ester **1a** (63 mg, 0.30 mmol) in THF (0.5 mL). The reaction was stirred for 10 min at –78 °C, at which point MeCN (0.5 mL) was added and the solvent was then removed *in vacuo*.

[10] For deprotonation of *N*-Boc-pyrrole, see: Hasan, I.; Marinelli, E. R.; Chang Lin, L.-C.; Fowler, F. W.; Levy, A. B. *J. Org. Chem.* **1981**, *46*, 157–164.

DMF (2.0 mL) was added at 23 ºC, followed by Umemoto reagent (161 mg, 0.400 mmol). The solution was stirred at 23 ºC for 3 h before the sequential addition of MeOH (2.0 mL), K_2CO_3 (124 mg, 0.900 mmol) and I_2 (102 mg, 0.400 mmol). The resulting solution was stirred for 4 h at 23 ºC, at which point a further batch of K_2CO_3 (124 mg, 0.900 mmol) and I_2 (102 mg, 0.400 mmol) was added. The resulting solution was stirred at 23 ºC for 14 h, at which point Et_2O/H_2O (1:1 v/v, 8 mL) and $PhCF_3$ (36.8 µL, 0.300 mmol) were added. A crude ^{19}F NMR yield (59%) was obtained by sampling the organic phase. To the mixture was added Et_2O/H_2O (1:1 v/v, 20 mL). The aqueous phase was extracted with Et_2O (2 × 10 mL), the combined organic phases washed with sat. aq. $NaHCO_3$ (20 mL) then 20% aq. $Na_2S_2O_3$ (20 mL), dried over $MgSO_4$, filtered and concentrated *in vacuo*. The crude product was purified by column chromatography (pentane: Et_2O = 100:0 to 99:1) to afford the title compound as a yellow oil in 57% yield (54 mg).

R_f (95/5 pentane/Et_2O): 0.67; **IR** (film) ν_{max}/cm^{-1}: 2930, 1754 (C=O), 1510, 1371, 1305, 1266, 1233, 1121, 952, 845, 794; **^{1}H NMR** (C_6D_6, 500 MHz) δ (ppm): 6.57 (d, *J* = 3.5 Hz, 1H), 5.76 (d, *J* = 3.5 Hz, 1H), 3.26 (tt, *J* = 11.5, 3.0 Hz, 1H), 1.98 (br. d, *J* = 13.0 Hz, 2H), 1.66 (dt, *J* = 13.0, 3.5 Hz, 2H), 1.58 (br. d, *J* = 15.0 Hz, 1H), 1.36 (s, 9H), 1.30 (qdd, *J* = 13.0, 3.5, 3.0 Hz, 2H), 1.17 (m, 2H), 1.08 (dtt, *J* = 13.0, 13.0, 3.5 Hz, 1H); **^{13}C NMR** (C_6D_6, 126 MHz) δ (ppm): 148.7, 147.3 (q, *J* = 2.0 Hz), 121.9 (q, *J* = 266.0 Hz), 121.8 (q, *J* = 39.0 Hz), 116.3 (q, *J* = 4.5 Hz), 107.1, 85.2, 37.0, 33.8, 27.3, 26.8, 26.5; **^{19}F NMR** (C_6D_6, 377 MHz) δ (ppm): –56.2; **HRMS** (ESI) mass calculated for $[M+Na]^+$ ($C_{16}H_{22}F_3NNaO_2^+$) requires *m/z* 340.1495, found *m/z* 340.1491.

tert-Butyl (*R*)-2-(4-(4-methoxyphenyl)butan-2-yl)-5-(trifluoromethyl)-1*H*-pyrrole-1-carboxylate (3o)

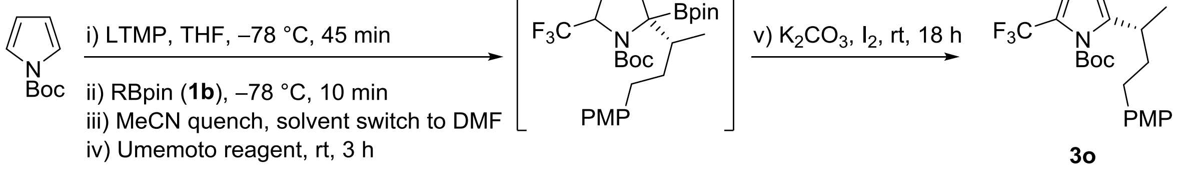

The title compound was synthesized with a modified General Procedure as shown below:

LTMP was freshly prepared by the dropwise addition of *n*BuLi (1.6 M in hexanes, 0.272 mL, 0.435 mmol) to 2,2,6,6-tetramethylpiperidine (0.435 M in THF, 1.00 mL, 0.435 mmol) at –78 °C. The solution was stirred

at –78 °C for 5 min, then warmed to 23 ºC for 5 min, before cooling to –78 °C. *N*-Boc-pyrrole (75 µL, 0.45 mmol) was added, and the solution stirred for 45 min at –78 °C before the addition of a solution of boronic ester **1b** (95:5 er, 87 mg, 0.30 mmol) in THF (0.5 mL). The reaction was stirred for 10 min at –78 °C, at which point MeCN (0.5 mL) was added and the solvent was then removed *in vacuo*. DMF (2.0 mL) was added at 23 ºC, followed by Umemoto reagent (161 mg, 0.400 mmol). The solution was stirred at 23 ºC for 3 h before the sequential addition of MeOH (2.0 mL), K_2CO_3 (124 mg, 0.900 mmol) and I_2 (102 mg, 0.400 mmol). The resulting solution was stirred for 4 h at 23 ºC, at which point a further batch of K_2CO_3 (124 mg, 0.900 mmol) and I_2 (102 mg, 0.400 mmol) was added. The resulting solution was stirred at 23 ºC for 14 h, at which point Et_2O/H_2O (1:1 v/v, 8 mL) and $PhCF_3$ (36.8 µL, 0.300 mmol) were added. A crude ^{19}F NMR yield (59%) was obtained by sampling the organic phase. To the mixture was added Et_2O/H_2O (1:1 v/v, 20 mL). The aqueous phase was extracted with Et_2O (2 × 10 mL), the combined organic phases washed with sat. aq. $NaHCO_3$ (20 mL) then 20% aq. $Na_2S_2O_3$ (20 mL), dried over $MgSO_4$, filtered and concentrated *in vacuo*. The crude product was purified by column chromatography (pentane: Et_2O = 100:0 to 98:2), followed by preparative TLC (hexane/Et_2O = 93/7), to afford the title compound as a yellow oil in 50% yield (59 mg).

$[\alpha]_D^{23}$ = +30 (*c* 0.67, $CHCl_3$); **R_f** (93/7 hexane/Et_2O): 0.36; **IR** (film) ν_{max}/cm^{-1}: 2934, 1755 (C=O), 1512, 1371, 1310, 1247, 1142, 1127, 1038, 850, 798; **^1H NMR** ($CDCl_3$, 500 MHz) δ (ppm): 7.05 (d, *J* = 8.5 Hz, 2H), 6.81 (d, *J* = 8.5 Hz, 2H), 6.63 (d, *J* = 3.5 Hz, 1H), 6.02 (d, *J* = 3.5 Hz, 1H), 3.78 (s, 3H), 3.42 (h, *J* = 7.0 Hz, 1H), 2.68 – 2.50 (m, 2H), 1.95 (ddt, *J* = 13.5, 9.0, 6.5 Hz, 1H), 1.76 (m, 1H), 1.58 (s, 9H), 1.27 (d, *J* = 7.0 Hz, 3H); **^{13}C NMR** ($CDCl_3$, 125 MHz) δ (ppm): 157.7, 148.5, 146.4 (q, *J* = 2.0 Hz), 134.3, 129.2, 121.4 (q, *J* = 39.0 Hz), 120.8 (q, *J* = 266.0 Hz), 115.6 (q, *J* = 4.5 Hz), 113.8, 106.8, 85.5, 55.2, 39.1, 32.7, 31.4, 27.5, 21.0; **^{19}F NMR** ($CDCl_3$, 377 MHz) δ (ppm): –56.7; **HRMS** (ESI) mass calculated for $[M+Na]^+$ ($C_{21}H_{26}F_3NNaO_3^+$) requires *m/z* 420.1757, found *m/z* 420.1750.

The er was determined by HPLC [chiralcel AD-H, hexane/isopropanol 99.9/0.1, 0.4 mL/min, 0 °C, λ = 230 nm, t (major) = 17.0 min, t (minor) = 18.5 min] to be 95:5 (100% es):

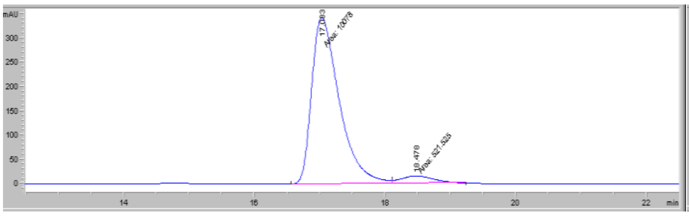

Peak #	RetTime [min]	Type	Width [min]	Area [mAU*s]	Height [mAU]	Area %
1	17.033	MF	0.4900	1.00780e4	342.78775	95.0797
2	18.478	FM	0.5746	521.52454	15.12636	4.9203

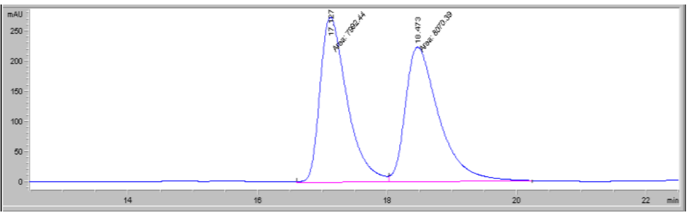

Peak #	RetTime [min]	Type	Width [min]	Area [mAU*s]	Height [mAU]	Area %
1	17.127	MF	0.4861	7992.43945	274.00565	49.7574
2	18.473	FM	0.6019	8070.38574	223.48727	50.2426

(*R*)-2-(4-Methyl-2-phenylpentan-2-yl)-5-(trifluoromethyl)furan (3p)

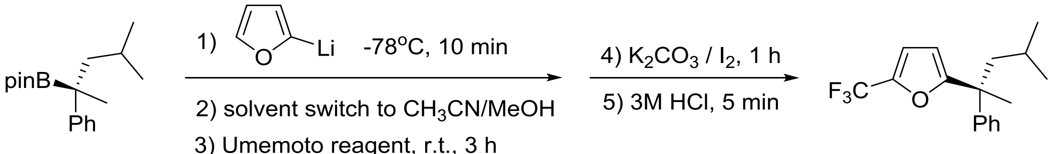

1p **3p**

The starting boronic ester **1p** (98:2 er, 86 mg, 0.30 mmol) was subjected to the General Procedure. The crude product was purified by preparative TLC (petroleum ether) to afford the title compound as a colourless oil in 32% yield (28 mg) and 98:2 er (100% es). Modified oxidation: To the reaction mixture (after reacting with Umemoto reagent for 3 h) was added $Cu(OAc)_2$ (109 mg, 0.60 mmol), TBAF (0.60 mmol, 0.60 mL in THF) and 4-tert-butylcatechol (199 mg, 1.20 mmol), and heated to 80 ℃ for 4 h to afford the title compound in 41% yield (36 mg) and 98:2 er (100% es).

$[\alpha]_D^{23}$ = –23 (*c* 1.0, $CHCl_3$); **R_f** (petroleum ether): 0.5; **IR** (film) ν_{max}/cm^{-1}: 2957, 1543, 1319, 1173, 1125, 1102, 1021, 798; **^1H NMR** ($CDCl_3$, 300 MHz) δ (ppm): 7.34 – 7.18 (m, 5H), 6.71 (dq, *J* = 3.6, 1.3 Hz, 1H), 6.16 (dq, *J* = 3.4, 0.8 Hz, 1H), 2.12 (dd, *J* = 14.0, 5.9 Hz, 1H), 1.96 (dd, *J* = 14.0, 5.1 Hz, 1H), 1.69 (s, 3H), 1.67 – 1.55 (m, 1H), 0.80 (d, *J* = 6.6 Hz, 3H), 0.74 (d, *J* = 6.7 Hz, 3H); **^{13}C NMR** ($CDCl_3$, 75 MHz) δ (ppm): 164.8 (q, *J* = 1.4 Hz), 146.6, 140.3 (q, *J* = 42.7 Hz), 128.3, 126.4, 126.3, 119.8 (q, *J* = 266.1 Hz), 112.1 (q, *J* = 2.8 Hz), 106.6, 49.1, 44.3, 25.3, 25.0, 24.8, 23.9; **^{19}F NMR** ($CDCl_3$, 283 MHz) δ (ppm): –63.76; **HRMS** (EI) mass calculated for $[M–C_4H_9]^+$ ($C_{13}H_{10}F_3O$) requires *m/z* 239.0684, found *m/z* 239.0685.

The er was determined by HPLC [chiralpak IB with guard, hexane/isopropanol 99.9/0.1, 0.5 mL/min, rt, λ = 210 nm, t (minor) = 9.3 min, t (major) = 9.7 min] to be 98:2 (100% es):

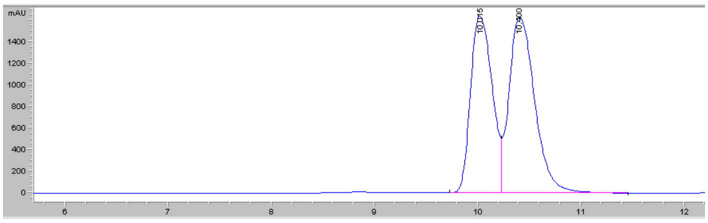

Peak #	RetTime [min]	Type	Width [min]	Area [mAU*s]	Height [mAU]	Area %
1	10.015	VV	0.2294	2.41476e4	1643.24561	46.0206
2	10.400	VB	0.2709	2.83237e4	1628.57825	53.9794

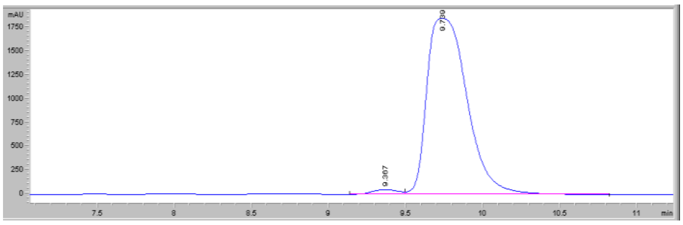

Peak #	RetTime [min]	Type	Width [min]	Area [mAU*s]	Height [mAU]	Area %
1	9.367	VV	0.1806	582.45990	50.46813	1.7065
2	9.739	VB	0.2841	3.35492e4	1845.70093	98.2935

2-((2*R,5*R**)-2-(*tert*-Butyl)-5-(trifluoromethyl)-2,5-dihydrofuran-2-yl)-4,4,5,5-tetramethyl-1,3,2-dioxaborolane (2q)**

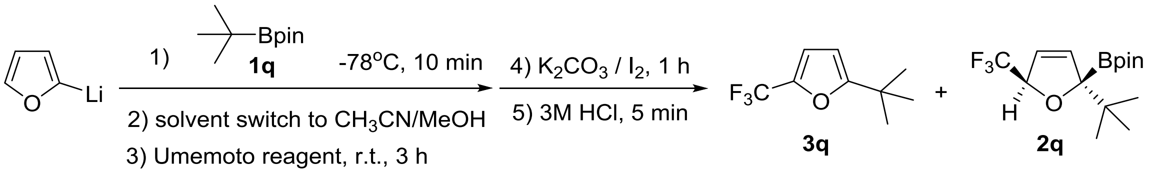

The starting boronic ester **1q** (110 mg, 0.600 mmol) was subjected to the General Procedure. $PhCF_3$ (0.60 mmol, 1.0 equiv) was added and a crude ^{19}F NMR yield [**3q** (34%), **2q** (37%)] obtained by sampling the organic phase. The crude product was purified by column chromatography (petroleum ether : ethyl acetate = 100:0 to 50:1) to afford the title compound **2q** as a colourless oil in 20% yield (38 mg, single diastereomer). The lower isolated yield of **2q** was attributed to its instability during column chromatography with silica gel. The relative configuration of **2q** was confirmed by NOESY NMR. Attempted isolation of **3q** was not successful due to its high volatility but was detected by ^{19}F NMR and GCMS.

2q: R_f (petroleum ether : ethyl acetate = 20:1): 0.3; **IR** (film) ν_{max}/cm^{-1}: 2978, 1354, 1135, 1079, 979, 854, 721; **^1H NMR** ($CDCl_3$, 400 MHz) δ (ppm): 6.15 (ddd, *J* = 6.3, 2.3, 1.0 Hz, 1H), 5.58 (dd, *J* = 6.2, 1.6 Hz, 1H), 5.07 – 4.95 (m, 1H), 1.19 (s, 6H), 1.18 (s, 6H), 0.88 (s, 9H); **^{13}C NMR** ($CDCl_3$, 100 MHz) δ (ppm): 136.5, 123.7 (q, *J* = 238.7 Hz), 119.1, 84.3 (q, *J* = 33.1 Hz), 84.1, 37.7, 25.9, 24.8, 24.4; **^{19}F NMR** ($CDCl_3$, 283 MHz) δ (ppm): –78.36; **^{11}B NMR** ($CDCl_3$, 96 MHz) δ 30.09; **HRMS** (ESI) mass calculated for $[M+Na]^+$ ($C_{15}H_{24}BF_3NaO_3$) requires *m/z* 343.1666, found *m/z* 343.1666.

2-Phenyl-5-(trifluoromethyl)furan (3r)

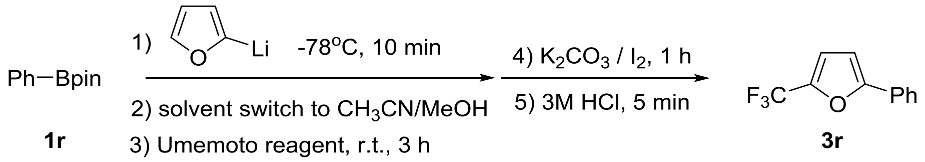

The starting boronic ester **1r** (61 mg, 0.30 mmol) was subjected to the General Procedure. The crude product was purified by preparative TLC (petroleum ether) to afford the title compound as a colourless oil in 47% yield (30 mg).

R$_f$ (petroleum ether): 0.5; **IR** (film) ν_{max}/cm^{-1}: 2927, 1370, 1319, 1105, 1022, 939, 758; **^{1}H NMR** ($CDCl_3$, 300 MHz) δ (ppm): 7.77 – 7.67 (m, 2H), 7.48 – 7.30 (m, 3H), 6.90 – 6.82 (m, 1H), 6.67 (dt, *J* = 3.6, 0.7 Hz, 1H); **^{13}C NMR** ($CDCl_3$, 75 MHz) δ (ppm): 156.5 (q, *J* = 1.4 Hz), 141.0 (q, *J* = 43.1 Hz), 128.9, 128.8, 124.6, 124.5, 119.2 (q, *J* = 269.2 Hz), 113.5 (q, *J* = 2.8 Hz), 105.3; **^{19}F NMR** ($CDCl_3$, 283 MHz) δ (ppm): –63.77; **HRMS** (EI) mass calculated for $[M]^+$ ($C_{17}H_7F_3O$) requires *m/z* 212.0449, found *m/z* 212.0452.

2-(3-Fluoro-4-methoxyphenyl)-5-(trifluoromethyl)furan (3s)

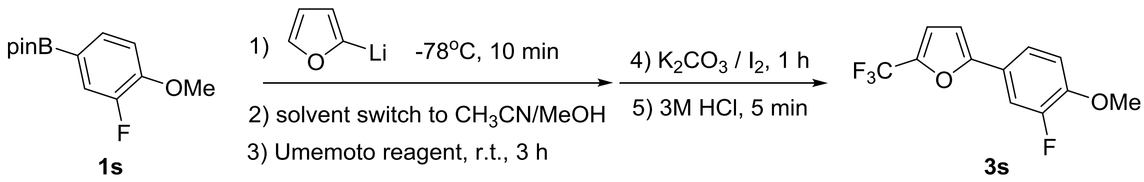

The starting boronic ester **1s** (76 mg, 0.30 mmol) was subjected to the General Procedure. The crude product was purified by column chromatography (petroleum ether : ethyl acetate = 100:1) to afford the title compound as a colourless oil in 55% yield (43 mg).

R$_f$ (petroleum ether): 0.1; **IR** (film) ν_{max}/cm^{-1}: 2949, 1609, 1498, 1273, 1131, 1100, 1020, 788; **^{1}H NMR** ($CDCl_3$, 300 MHz) δ (ppm): 7.45 – 7.38 (m, 2H), 6.99 (t, *J* = 8.4 Hz, 1H), 6.83 – 6.80 (m, 1H), 6.54 (d, *J* = 3.5 Hz, 1H), 3.91 (s, 3H); **^{13}C NMR** ($CDCl_3$, 75 MHz) δ (ppm): 155.3 (q, *J* = 1.4 Hz), 152.5 (d, *J* = 244.1 Hz), 148.2 (d, *J* = 10.9 Hz), 140.7 (q, *J* = 42.7 Hz), 122.7 (d, *J* = 7.3 Hz), 120.7 (d, *J* = 3.6 Hz), 119.3 (q, *J* = 266.3 Hz), 113.6 (d, *J* = 2.4 Hz), 113.5 (q, *J* = 2.8 Hz), 112.6 (d, *J* = 20.7 Hz), 104.7, 56.3; **^{19}F NMR**

($CDCl_3$, 283 MHz) δ (ppm): –63.77 (3F), –134.26 (1F); **HRMS** (EI) mass calculated for $[M]^+$ ($C_{12}H_8F_4O_2$) requires *m/z* 260.0460, found *m/z* 260.0464.

2-(4-Chlorophenyl)-5-(trifluoromethyl)furan (3t)

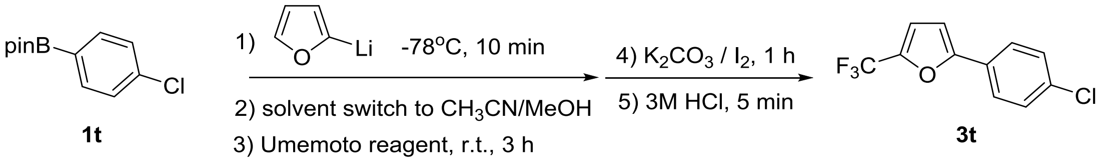

The starting boronic ester **1t** (72 mg, 0.30 mmol) was subjected to the General Procedure. The crude product was purified by preparative TLC (petroleum ether) to afford the title compound as a colourless oil in 39% yield (29 mg).

R_f (petroleum ether): 0.5; **IR** (film) ν_{max}/cm^{-1}: 1599, 1482, 1320, 1175, 1131, 1092, 940, 792; **^{1}H NMR** ($CDCl_3$, 500 MHz) δ (ppm): 7.64 (d, *J* = 8.7 Hz, 2H), 7.39 (d, *J* = 8.7 Hz, 2H), 6.86 (dq, *J* = 3.6, 1.2 Hz, 1H), 6.67 (dq, *J* = 3.5, 0.8 Hz, 1H); **^{13}C NMR** ($CDCl_3$, 125 MHz) δ (ppm): 155.2 (q, *J* = 1.5 Hz), 141.2 (q, *J* = 42.4 Hz), 134.6, 129.1, 127.7, 125.6, 119.0 (q, *J* = 268.1 Hz), 113.4 (q, *J* = 2.9 Hz), 105.6; **^{19}F NMR** ($CDCl_3$, 283 MHz) δ (ppm): –63.83; **HRMS** (EI) mass calculated for $[M]^+$ ($C_{11}H_6F_3OCl$) requires *m/z* 246.0059, found *m/z* 246.0054.

Trimethyl(4-(5-(trifluoromethyl)furan-2-yl)phenyl)silane (3u)

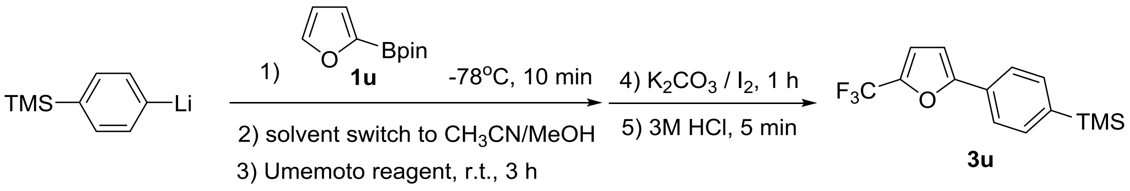

The title compound was synthesized with a modified General Procedure as shown below:

To a stirred solution of (4-bromophenyl)trimethylsilane (92 mg, 0.40 mmol) in 1.5 mL THF at –78 °C under N_2 was added *n*BuLi (1.6 M in hexane, 244 µL, 0.390 mmol) dropwise. The resulting solution was stirred for 0.5 h before the addition of a solution of furan-2-boronic acid pinacol ester **1u** (58 mg, 0.30 mmol) in THF (0.5 mL) at –78 °C. The reaction was stirred for 10 min at –78 °C before removing the solvent under vacuum. MeOH (1 mL) and CH_3CN (1 mL) were added at 23 °C followed by Umemoto reagent (161 mg,

0.400 mmol). The solution was stirred at 23 °C for 3 h before the sequential addition of K_2CO_3 (124 mg, 0.900 mmol) and I_2 (102 mg, 0.400 mmol). The resulting solution was stirred for 1 h at 23 °C before the addition of a solution of HCl (3 M in water, 1 mL) and stirring continued for a further 5 min. Water (10 mL) was added and the product extracted with Et_2O (2 × 15 mL). The organic phases were washed with sat. aq. $NaHCO_3$ then 20% aq. $Na_2S_2O_3$, dried over $MgSO_4$, filtered and concentrated *in vacuo*. The crude product purified by preparative TLC (petroleum ether) to afford the title compound as a colourless oil in 43% yield (37 mg).

R_f (petroleum ether): 0.6; **IR** (film) ν_{max}/cm^{-1}: 2957, 1562, 1320, 1174, 1129, 1112, 1021, 940, 837, 790, 757; **^{1}H NMR** ($CDCl_3$, 500 MHz) δ (ppm): 7.72 (d, *J* = 8.1 Hz, 2H), 7.61 (d, *J* = 8.1 Hz, 2H), 6.89 (dq, *J* = 3.6, 1.2 Hz, 1H), 6.72 (dq, *J* = 3.5, 0.8 Hz, 1H), 0.35 (s, 9H); **^{13}C NMR** ($CDCl_3$, 125 MHz) δ (ppm): 156.5 (q, *J* = 1.5 Hz), 141.6, 140.9 (q, *J* = 43.2 Hz), 133.8, 129.5, 123.6, 119.0 (q, *J* = 268.7 Hz), 113.4 (q, *J* = 2.9 Hz), 105.4, –1.22; **^{19}F NMR** ($CDCl_3$, 283 MHz) δ (ppm): –63.75; **HRMS** (EI) mass calculated for $[M]^+$ ($C_{14}H_{15}F_3OSi$) requires *m/z* 284.0844, found *m/z* 284.0833.

2-(2-Methoxyphenyl)-5-(trifluoromethyl)furan (3v)

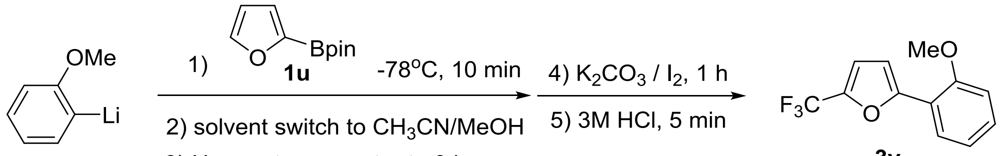

The title compound was synthesized with a modified General Procedure as shown below:

To a stirred solution of 1-bromo-2-methoxybenzene (75 mg, 0.40 mmol) in 1.5 mL THF at –78 °C under N_2 was added *n*BuLi (1.6 M in hexane, 244 µL, 0.390 mmol) dropwise. The resulting solution was stirred for 0.5 h before the addition of a solution of furan-2-boronic acid pinacol ester **1u** (58 mg, 0.30 mmol) in THF (0.5 mL) at –78 °C. The reaction was stirred for 10 min at –78 °C before removing the solvent under vacuum. MeOH (1 mL) and CH_3CN (1 mL) were added at 23 °C followed by Umemoto reagent (161 mg, 0.400 mmol). The solution was stirred at 23 °C for 3 h before the sequential addition of K_2CO_3 (124 mg, 0.900 mmol) and I_2 (102 mg, 0.400 mmol). The resulting solution was stirred for 1 h at 23 °C before the addition of a solution of HCl (3 M in water, 1 mL) and stirring continued for a further 5 min. Water (10 mL)

was added and the product extracted with Et_2O (2 × 15 mL). The organic phases were washed with sat. aq. $NaHCO_3$ then 20% aq. $Na_2S_2O_3$, dried over $MgSO_4$, filtered and concentrated *in vacuo*. The crude product purified by preparative TLC (petroleum ether) to afford the title compound as a colourless oil in 45% yield (33 mg).

R$_f$ (petroleum ether): 0.4; **IR** (film) ν_{max}/cm^{-1}: 2946, 2841, 1600, 1492, 1319, 1123, 1103, 1020, 940, 803; **^{1}H NMR** ($CDCl_3$, 300 MHz) δ (ppm): 7.88 (dd, *J* = 7.8, 1.7 Hz, 1H), 7.31 (t, *J* = 7.9 Hz, 1H), 7.10 – 6.93 (m, 3H), 6.86 (dq, *J* = 3.6, 1.2 Hz, 1H), 3.94 (s, 3H). **^{13}C NMR** ($CDCl_3$, 75 MHz) δ (ppm): 156.1, 152.8 (q, *J* = 1.7 Hz), 139.9 (q, *J* = 41.9 Hz), 129.6, 126.6, 120.9, 119.5 (q, *J* = 266.3 Hz), 118.4, 113.5 (q, *J* = 2.9 Hz), 111.1, 110.1, 55.5; **^{19}F NMR** ($CDCl_3$, 283 MHz) δ (ppm): –63.62; **HRMS** (EI) mass calculated for $[M]^+$ ($C_{12}H_9F_3O_2$) requires *m/z* 242.0555, found *m/z* 242.0558.

5-(5-(Trifluoromethyl)furan-2-yl)benzo[*d*][1,3]dioxole (3w)

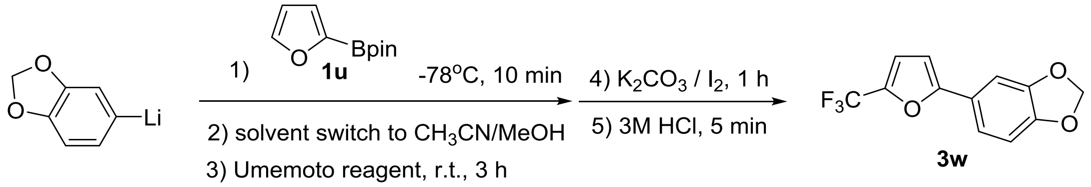

The title compound was synthesized with a modified General Procedure as shown below:

To a stirred solution of 1-bromo-3,4-(methylenedioxy)benzene (80 mg, 0.40 mmol) in 1.5 mL THF at –78 °C under N_2 was added *n*BuLi (1.6 M in hexane, 244 µL, 0.390 mmol) dropwise. The resulting solution was stirred for 0.5 h before the addition of a solution of furan-2-boronic acid pinacol ester **1u** (58 mg, 0.30 mmol) in THF (0.5 mL) at –78 °C. The reaction was stirred for 10 min at –78 °C before removing the solvent under vacuum. MeOH (1 mL) and CH_3CN (1 mL) were added at 23 °C followed by Umemoto reagent (161 mg, 0.400 mmol). The solution was stirred at 23 °C for 3 h before the sequential addition of K_2CO_3 (124 mg, 0.900 mmol) and I_2 (102 mg, 0.400 mmol). The resulting solution was stirred for 1 h at 23 °C before the addition of a solution of HCl (3 M in water, 1 mL) and stirring continued for a further 5 min. Water (10 mL) was added and the product extracted with Et_2O (2 × 15 mL). The organic phases were washed with sat. aq. $NaHCO_3$ then 20% aq. $Na_2S_2O_3$, dried over $MgSO_4$, filtered and concentrated *in vacuo*.

The crude product purified by preparative TLC (petroleum ether) to afford the title compound as a colourless oil in 73% yield (56 mg).

R_f (petroleum ether : ethyl acetate = 20:1): 0.3; **IR** (film) ν_{max}/cm^{-1}: 2900, 1480, 1316, 1225, 1172, 1099, 1038, 937, 866, 786; **^{1}H NMR** ($CDCl_3$, 300 MHz) δ (ppm): 7.20 (d, *J* = 8.0 Hz, 1H), 7.15 (s, 1H), 6.88 – 6.79 (m, 2H), 6.50 (dt, *J* = 3.6, 0.8 Hz, 1H), 5.99 (s, 2H).; **^{13}C NMR** ($CDCl_3$, 75 MHz) δ (ppm): 156.3 (q, *J* = 1.4 Hz), 148.3, 141.6, 140.4 (q, *J* = 42.5 Hz), 123.7, 119.3 (q, *J* = 267.8 Hz), 118.8, 113.5 (q, *J* = 3.0 Hz), 108.7, 105.1, 104.2, 101.5; **^{19}F NMR** ($CDCl_3$, 283 MHz) δ (ppm): –63.71; **HRMS** (EI) mass calculated for $[M]^+$ ($C_{12}H_7F_3O_3$) requires *m/z* 256.0347, found *m/z* 256.0342.

(*R*)-2-(Cyclohepta-2,4,6-trien-1-yl)-5-(4-(4-methoxyphenyl)butan-2-yl)furan (4a)

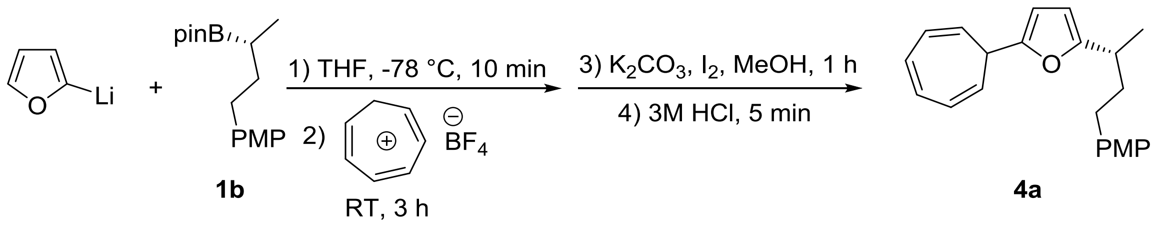

To a stirred solution of furan (0.40 M in THF, 1.0 ml, 0.40 mmol) under N_2 at –78 °C was added *n*BuLi (1.6 M in hexane, 244 µL, 0.390 mmol) dropwise. The resulting solution was stirred for 1 h at 23 °C before the addition of a solution of boronic ester **1b** (95:5 er, 87 mg, 0.30 mmol) in THF (0.5 mL) at –78 °C. The reaction was stirred for 10 min at –78 °C before the addition of tropylium tetrafluoroborate (71 mg, 0.40 mmol) in one portion. The reaction was removed from the cold bath and stirred at 23 ºC for 3 h. MeOH (1.0 mL) was added, followed sequentially by K_2CO_3 (124 mg, 0.900 mmol) and I_2 (102 mg, 0.400 mmol). The resulting solution was stirred for 1 h at 23 °C before the addition of a solution of HCl (3 M in water, 1.0 mL). The mixture was stirred for 5 min before water (10 mL) was added and the product extracted into Et_2O (2 × 15 mL). The organic phases were washed with sat. aq. $NaHCO_3$ then 20% aq. $Na_2S_2O_3$, dried over $MgSO_4$, filtered and concentrated *in vacuo*. The residue was purified by preparative TLC (petroleum ether : ethyl acetate = 50:1) to give the title compound as a colourless oil in 43% yield (41 mg) and 95:5 er (100% es).

$[\alpha]_D^{23}$ –17 (*c* 1.00, $CHCl_3$); **R_f** (petroleum ether : ethyl acetate = 50:1): 0.2; **IR** (film) ν_{max}/cm^{-1}: 2931, 1510, 1243, 1176, 1037, 782, 702; **^{1}H NMR** ($CDCl_3$, 300 MHz) δ (ppm): 7.15 – 7.05 (m, 2H), 6.88 – 6.79 (m, 2H), 6.73 (ddd, *J* = 3.7, 2.6, 0.8 Hz, 2H), 6.33 – 6.21 (m, 2H), 6.13 (dd, *J* = 3.1, 1.0 Hz, 1H), 5.97 (dd, *J* = 3.0, 0.8 Hz, 1H), 5.50 (ddt, *J* = 9.5, 5.6, 0.7 Hz, 2H), 3.79 (s, 3H), 2.92 – 2.76 (m, 2H), 2.57 (t, *J* = 8.0 Hz, 2H), 2.08 – 1.92 (m, 1H), 1.89 – 1.71 (m, 1H), 1.28 (d, *J* = 7.0 Hz, 3H); **^{13}C NMR** ($CDCl_3$, 75 MHz) δ (ppm): 159.5, 157.8, 154.3, 134.5, 131.1, 129.3, 124.8, 123.6, 123.5, 113.8, 105.1, 104.2, 55.3, 39.3, 37.9, 32.8, 32.6, 19.3; **HRMS** (EI) mass calculated for $[M]^+$ ($C_{22}H_{24}O_2$) requires *m/z* 320.1776, found *m/z* 320.1771.

The er was determined by HPLC [chiralpak IB with guard, hexane 100%, 0.5 mL/min, rt, λ = 210 nm, t (minor) = 45.2 min, t (major) = 52.1 min] to be 95:5 (100% es):

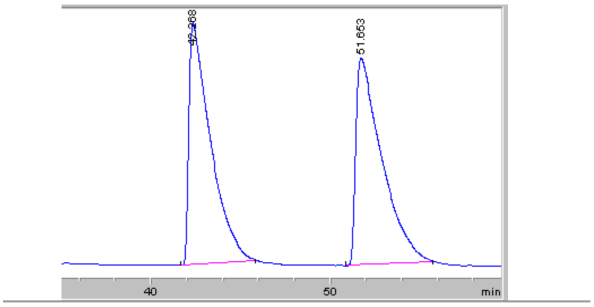

Signal 1: DAD1 C, Sig=210,8 Ref=360,100

Peak #	RetTime [min]	Type	Width [min]	Area [mAU*s]	Height [mAU]	Area %
1	42.268	PB	1.0954	2.63358e4	310.53400	49.6283
2	51.653	PB	1.3121	2.67303e4	264.26456	50.3717

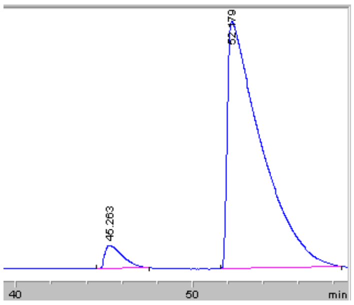

Signal 1: DAD1 C, Sig=210,8 Ref=360,100

Peak #	RetTime [min]	Type	Width [min]	Area [mAU*s]	Height [mAU]	Area %
1	45.263	BB	0.9359	4141.03955	59.50417	4.6999
2	52.179	PB	1.6402	8.39686e4	631.11951	95.3001

(*R*)-2-(Benzo[*d*][1,3]dithiol-2-yl)-5-(4-(4-methoxyphenyl)butan-2-yl)furan (4b)

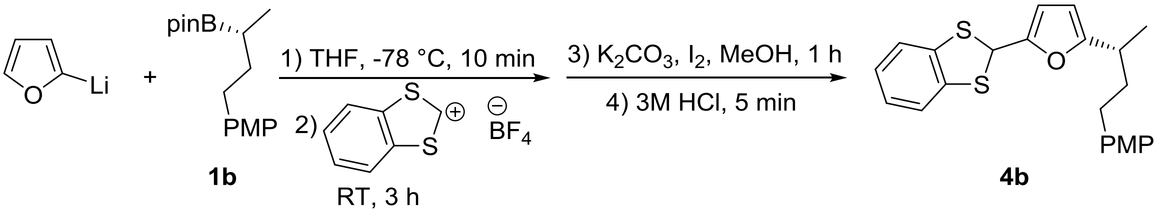

To a stirred solution of furan (0.40 M in THF, 1.0 ml, 0.40 mmol) under N_2 at –78 °C was added *n*BuLi (1.6 M in hexane, 244 µL, 0.390 mmol) dropwise. The resulting solution was stirred for 1 h at 23 °C before the addition of a solution of boronic ester **1b** (95:5 er, 87 mg, 0.30 mmol) in THF (0.5 mL) at –78 °C. The reaction was stirred for 10 min at –78 °C before the addition of 1,3-benzodithiolylium tetrafluoroborate (96 mg, 0.40 mmol) in one portion. The reaction was removed from the cold bath and stirred at 23 °C for 3 h. MeOH (1.0 mL) was added, followed sequentially by K_2CO_3 (124 mg, 0.900 mmol) and I_2 (102 mg,

0.400 mmol). The resulting solution was stirred for 1 h at 23 °C before the addition of a solution of HCl (3 M in water, 1.0 mL). The mixture was stirred for 5 min before water (10 mL) was added and the product extracted into Et_2O (2 × 15 mL). The organic phases were washed with sat. aq. $NaHCO_3$ then 20% aq. $Na_2S_2O_3$, dried over $MgSO_4$, filtered and concentrated *in vacuo*. The residue was purified by preparative TLC (petroleum ether : ethyl acetate = 50:1) to give the title compound as a colourless oil in 75% yield (86 mg) and 95:5 er (100% es).

$[\alpha]_D^{23}$ –12 (*c* 1.00, $CHCl_3$); **R_f** (petroleum ether : ethyl acetate = 50:1): 0.1; **IR** (film) ν_{max}/cm^{-1}: 2932, 1602, 1510, 1444, 1243, 1150, 1036, 820, 741; **^{1}H NMR** ($CDCl_3$, 300 MHz) δ (ppm): 7.25 – 7.17 (m, 2H), 7.10 – 7.01 (m, 4H), 6.83 – 6.79 (m, 2H), 6.28 (dd, *J* = 3.2, 0.8 Hz, 1H), 6.00 (s, 1H), 5.91 – 5.87 (m, 1H), 3.78 (s, 3H), 2.77 (h, *J* = 7.0 Hz, 1H), 2.51 (t, *J* = 8.0 Hz, 2H), 2.00 – 1.83 (m, 1H), 1.83 – 1.65 (m, 1H), 1.22 (d, *J* = 7.0 Hz, 3H); **^{13}C NMR** ($CDCl_3$, 75 MHz) δ (ppm): 161.2, 157.7, 150.5, 136.9, 134.4, 129.4, 125.7, 122.2, 113.8, 108.5, 104.8, 55.3, 48.6, 37.8, 32.6, 32.4, 19.0; **HRMS** (EI) mass calculated for $[M]^+$ ($C_{22}H_{22}O_2S_2$) requires *m/z* 382.1061, found *m/z* 382.1053.

The er was determined by HPLC [chiralpak IB with guard, hexane/isopropanol 99.9/0.1, 1.0 mL/min, rt, λ = 273 nm, t (minor) = 33.4 min, t (major) = 36.5 min] to be 95:5 (100% es):

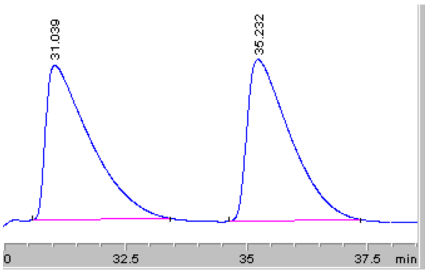

Signal 1: DAD1 A, Sig=273,100 Ref=360,100

Peak #	RetTime [min]	Type	Width [min]	Area [mAU*s]	Height [mAU]	Area %
1	31.039	PB	0.8741	4657.00391	72.63110	49.7584
2	35.232	BB	0.8845	4702.23486	75.93438	50.2416

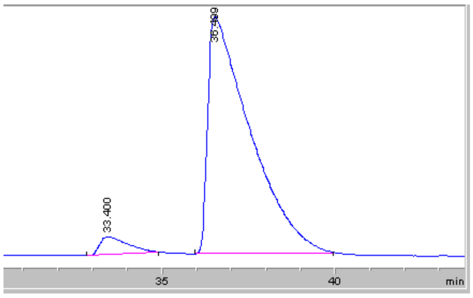

Signal 1: DAD1 A, Sig=273,100 Ref=360,100

Peak #	RetTime [min]	Type	Width [min]	Area [mAU*s]	Height [mAU]	Area %
1	33.400	PB	0.7549	724.24225	12.74574	4.7878
2	36.499	BB	1.1345	1.44027e4	168.62425	95.2122

2-(Benzo[*d*][1,3]dithiol-2-yl)-5-((1*R*,2*S*,5*R*)-2-isopropyl-5-methylcyclohexyl)furan (4c)

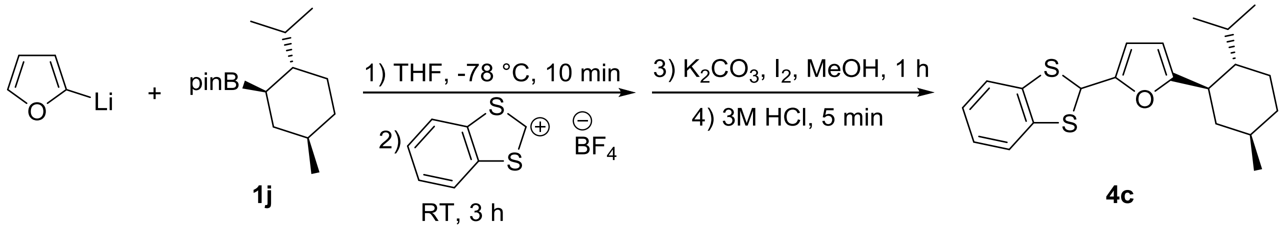

To a stirred solution of furan (0.40 M in THF, 1.0 ml, 0.40 mmol) under N_2 at –78 °C was added *n*BuLi (1.6 M in hexane, 244 µL, 0.390 mmol) dropwise. The resulting solution was stirred for 1 h at 23 °C before the addition of a solution of boronic ester **1j** (>95:5 dr, 80 mg, 0.30 mmol) in THF (0.5 mL) at –78 °C. The reaction was stirred for 10 min at –78 °C before the addition of 1,3-benzodithiolylium tetrafluoroborate (96 mg, 0.40 mmol) in one portion. The reaction was removed from the cold bath and stirred at 23 ºC for 3 h. MeOH (1.0 mL) was added, followed sequentially by K_2CO_3 (124 mg, 0.900 mmol) and I_2 (102 mg, 0.400 mmol). The resulting solution was stirred for 16 h at 23 °C before the addition of a solution of HCl (3 M in water, 1.0 mL). The mixture was stirred for 5 min before water (10 mL) was added and the product extracted into Et_2O (2 × 15 mL). The organic phases were washed with sat. aq. $NaHCO_3$ then 20% aq. $Na_2S_2O_3$, dried over $MgSO_4$, filtered and concentrated *in vacuo*. The residue was purified by preparative TLC (petroleum ether : Et_2O = 200:1) to give the title compound as a colourless oil in 80% yield (86 mg) and >95:5 dr (100% ds).

$[\alpha]_D^{23}$ –12 (*c* 1.00, $CHCl_3$); $\mathbf{R_f}$ 0.37 (100% pet. ether); **IR** (ν_{max}/cm^{-1}, neat): 3059, 2952–2850, 1544, 1444, 1434, 1367, 1260, 1172, 1118, 1013; **^{1}H NMR** (500 MHz, $CDCl_3$) δ 7.24 – 7.20 (m, 2H), 7.06 – 7.02 (m, 2H), 6.27 (dd, *J* = 3.1, 0.7 Hz, 1H), 6.01 (d, *J* = 1.0 Hz, 1H), 5.85 (d, *J* = 3.2 Hz, 1H), 2.50 (ddd, *J* = 12.1, 11.2, 3.4 Hz, 1H), 1.81 (dddd, *J* = 12.8, 3.5, 3.5, 2.2 Hz, 1H), 1.76 (dddd, *J* = 12.5, 5.6, 3.3, 3.3 Hz, 1H), 1.68 (dddd, *J* = 12.8, 3.2, 3.2, 3.2 Hz, 1H), 1.49 – 1.33 (m, 3H), 1.20 (app. q, *J* = 12.2 Hz, 1H), 1.07 (app. qd, *J* = 12.8, 3.2 Hz, 1H), 0.99 – 0.92 (m, 1H), 0.90 (d, *J* = 6.5 Hz, 3H), 0.83 (d, *J* = 7.0 Hz, 3H), 0.68 (d, *J* = 6.9 Hz, 3H); **^{13}C NMR** (126 MHz, $CDCl_3$) δ 160.6, 149.8, 137.0, 136.95, 125.5, 122.1, 108.3, 105.1, 48.7, 47.0, 41.7, 40.9, 34.9, 32.8, 24.8, 22.4, 21.1, 15.8; **HRMS** (ESI$^+$) calcd. For $[M+Na]^+$ ($C_{21}H_{26}NaOS_2$) 381.1317, found 381.1315.

5. Examples of Unreactive/Challenging Substrates

The following boronate complexes all gave <5% yield of the corresponding three-component coupling products. This was either due to them being unreactive in the trifluoromethylation reaction or the failure of the resulting intermediate **2** to be oxidized under various conditions (due to steric hindrance).

Trifluoromethylation failed:

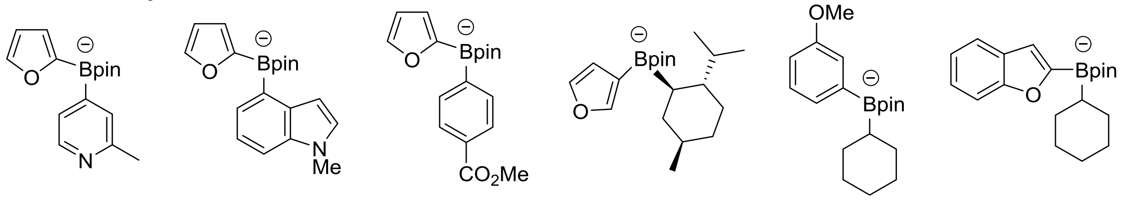

Oxidation of intermediate 2 failed:

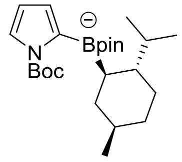

6. EPR Experiments

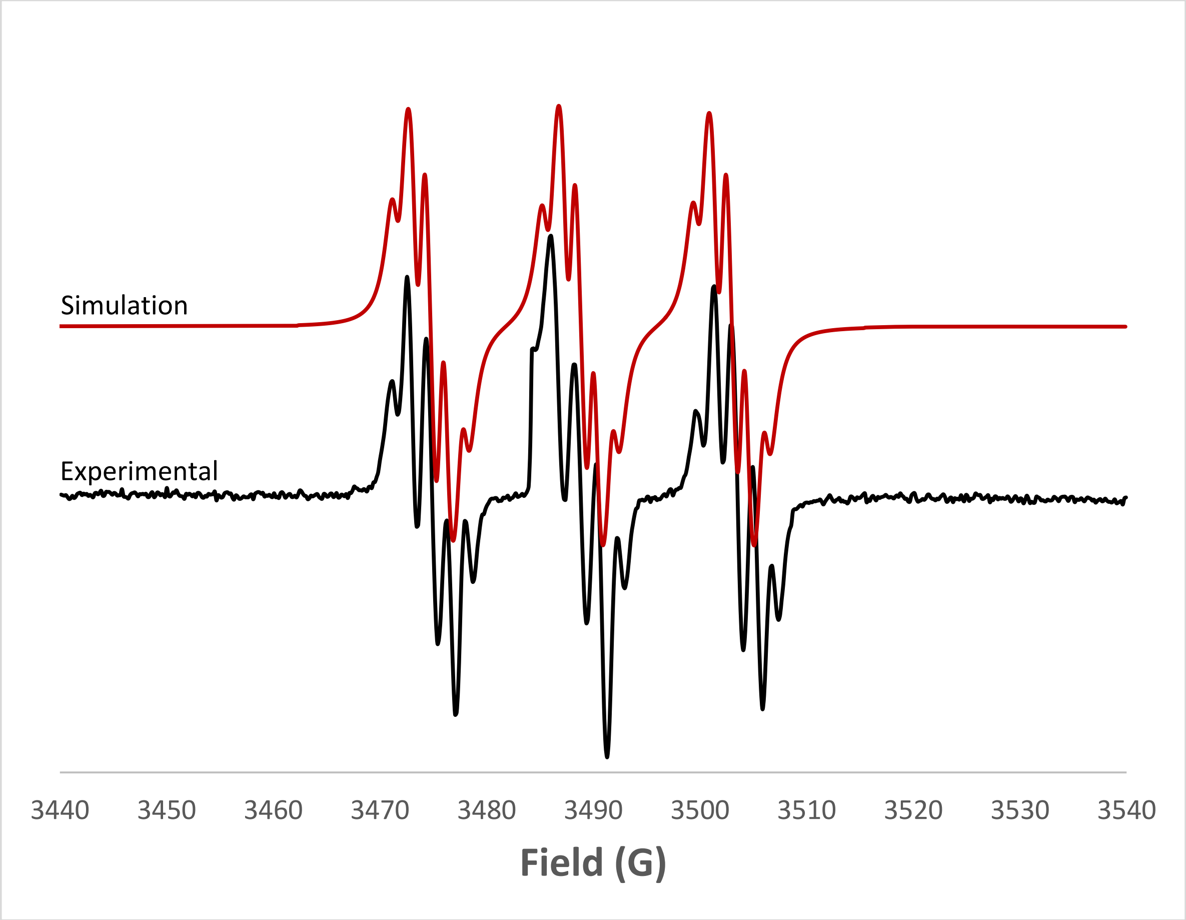

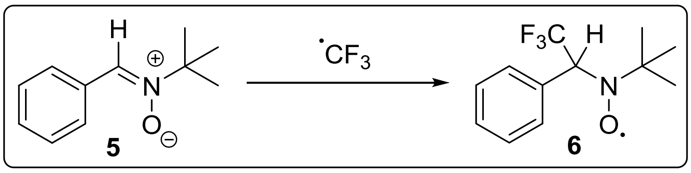

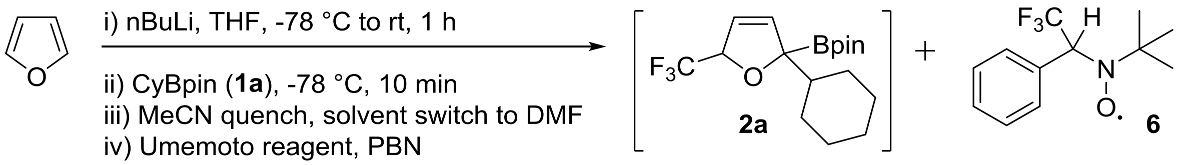

Procedure:

To a stirred solution of furan (0.40 M in THF, 1.0 mL, 0.40 mmol) under N_2 at –78 °C was added *n*BuLi (1.6 M in hexanes, 0.244 mL, 0.390 mmol) dropwise. The resulting solution was stirred for 1 h at 23 ºC before the addition of a solution of cyclohexylboronic acid pinacol ester **1a** (63 mg, 0.30 mmol) in THF (0.5 mL) at –78 °C. The reaction was stirred for 10 min at –78 °C, at which point MeCN (0.5 mL) was added and the solvent was then removed *in vacuo*. DMF (3.0 mL) was added at 23 ºC, followed by Umemoto reagent (161 mg, 0.400 mmol) and *N*-*tert*-butyl-α-phenylnitrone (PBN, **5**, 106 mg, 0.600 mmol) simultaneously. The

EPR spectrum of radical **6** was detected approximately 10 min later. After 3 h, the ^{19}F NMR yield of intermediate **2a** was found to be 9% (1.3:1.0 dr), suggesting that the trapping of the CF_3 radical by PBN inhibits the reaction.

Spectrometer Details:

Active Spectrum X-band Micro-ESR Spectrometer.

Run Details:

2 mm diameter quartz capillary, 40 scans, 75 ms delay, field sweep 3420–3600 G, power 15 mW, modulation amplitude 40%, microwave frequency 9.787784 GHz, temperature 25 °C.

Simulation Results (using EasySpin):

g = 2.0044, a_N = 1.411 mT, a_H = 0.1664 mT, a_F = 0.1781 mT.

Literature Examples of CF_3-PBN spin trap:

1. Spell, M. L.; Deveaux, K.; Bresnahan, C. G.; Bernard, B. L.; Sheffield, W.; Kumar, R.; Ragains, J. R. *Angew. Chem. Int. Ed.* **2016**, *55*, 6515–6519:
   a_N = 1.426 mT, a_H = 0.166 mT, a_F = *not quoted* (in MeCN).
2. Cheng, Y.; Yuan, X.; Ma, J.; Yu, S. *Chem. Eur. J.* **2015**, *21*, 8355–8359:
   Hyperfine coupling constants not quoted (in DMF).
3. Zhang, C.-P.; Wang, H.; Klein, A.; Biewer, C.; Stirnat, K.; Yamaguchi, Y.; Xu, L.; Gomez-Benitez, V.; Vicic, D. A. *J. Am. Chem. Soc.* **2013**, *135*, 8141–8144:
   a_N = 1.41 mT, a_H = 0.117 mT, a_F = 0.177 mT (in THF/*n*$Bu_4N[PF_6]$).
4. Cai, S.; Chen, C.; Sun, Z.; Xi, C. *Chem. Commun.* **2013**, *49*, 4552–4554:
   a_N = 1.41 mT, a_H = 0.24 mT, a_F = 0.14 mT (in *t*BuOH).
5. Haire, D. L.; Oehler, U. M.; Krygsman, P. H.; Janzen, E. G. *J. Org. Chem.* **1988**, *53*, 4535–4542:
   a_N = 1.397 mT, a_H = 0.185 mT, a_F = 0.154 mT (in benzene).
6. Okhlobystina, L. V.; Cherkasova, T. I.; Tyurikov, V. A. *Russ. Chem. Bull.* **1979**, 28, 2036–2043:
   a_N = 1.33 mT, a_H = 0.15 mT, a_F = 0.15 mT (in benzene).
   a_N = 1.41 mT, a_H = 0.18 mT, a_F = 0.159 mT (in DMF).
7. Janzen, E. G.; Blackburn, B. J. *J. Am. Chem. Soc.* **1968**, *90*, 5909–5910:
   a_N = 1.33 mT, a_H = 0.154 mT, a_F = 0.154 mT (in benzene).

EasySpin Script (for MATLAB):

Least Squares Fitting (example):

```
Sys0.g = 2.004;
Sys0.Nucs = '14N,1H,19F';
Sys0.n = [1 1 3];
Sys0.A = [39.548 3.2817 4.965];
Sys0.lwpp = [0.07 0.07];
Vary.g = 0.005;
```

```
Vary.A = [2 2 2];
Vary.lwpp = [0.1 0.1];
Exp.mwFreq = 9.787784;
Exp.Range = [342 360];
esfit('garlic',spc,Sys0,Vary,Exp);
```

Spectral Simulation:

```
Sys = struct('g',2.0044,'Nucs','14N,1H,19F','n',[1 1 3],'A',[39.5888 3.26672 4.99763],'lwpp',[0.01805142 0.106953]);
Exp = struct('mwFreq',9.787784,'range',[342 360]);
garlic(Sys,Exp);
```

EasySpin Reference: Stoll, S.; Schweiger, A. *J. Magn. Reson.* **2006**, *178*, 42–55. www.easyspin.org

EPR Control Experiments

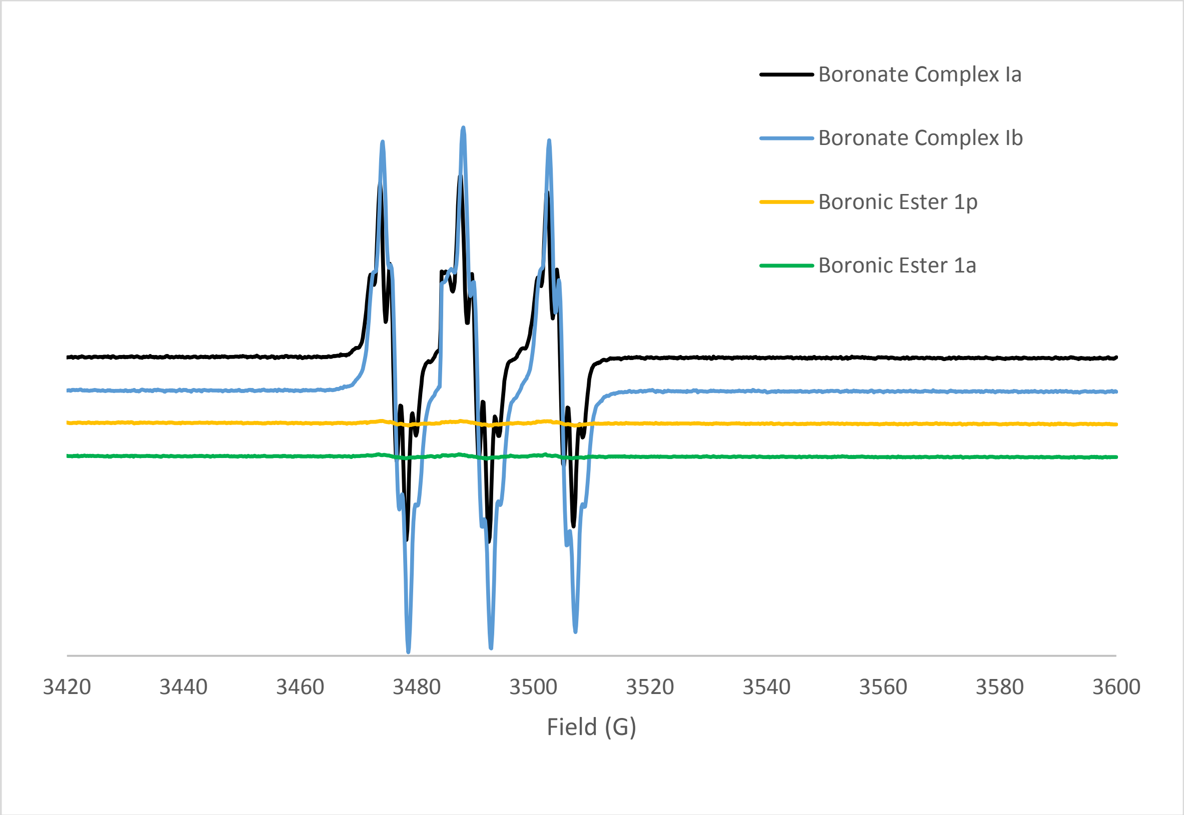

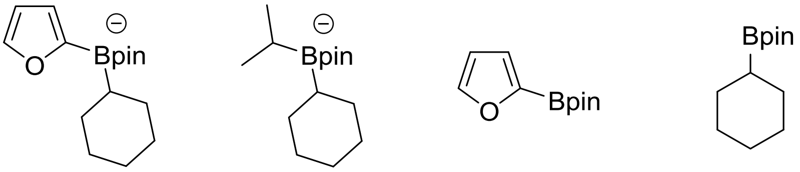

Ia **Ib** **1q** **1a**

Spectra recorded following the simultaneous addition of Umemoto reagent (161 mg, 0.400 mmol) and *N*-*tert*-butyl-α-phenylnitrone (PBN, **5**, 106 mg, 0.600 mmol) to a solution of boronate complex **Ia**, boronate

complex **Ib**, boronic ester **1q** or boronic ester **1a** (0.300 mmol, 0.10 M in DMF). Results demonstrate that the reaction of the Umemoto reagent with a boronate complex leads to the formation of a CF_3 radical.

Additional EPR References:

Electron Paramagnetic Resonance: A Practitioner's Toolkit; Brustolon, M., Giamello, E., Eds.; John Wiley & Sons, Inc.: New Jersey, 2009.

Gerson, F.; Huber, W. *Electron Spin Resonance Spectroscopy of Organic Radicals*; Wiley-VCH: Weinheim, 2003.

7. ^{1}H, ^{13}C and ^{19}F NMR spectra

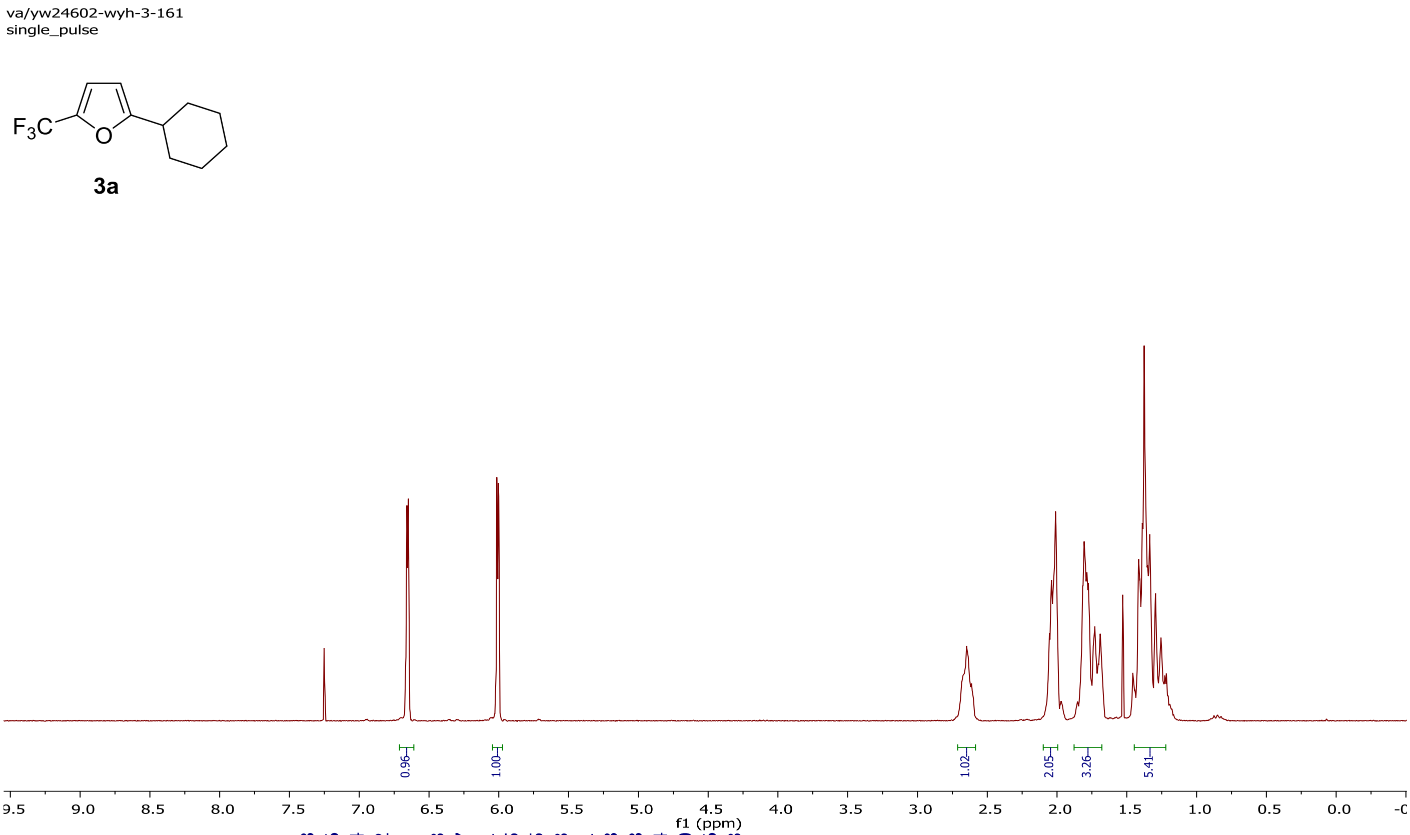

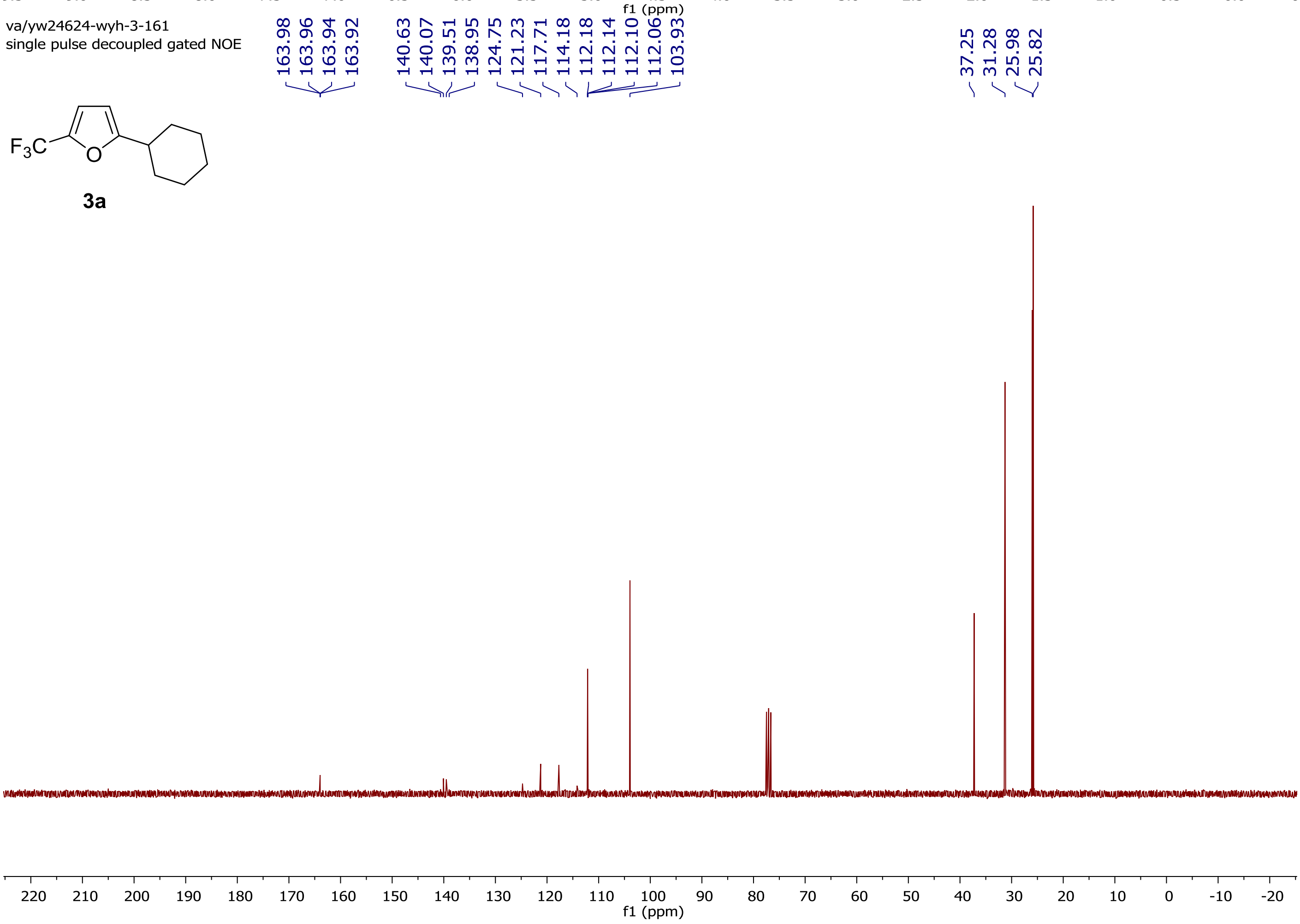

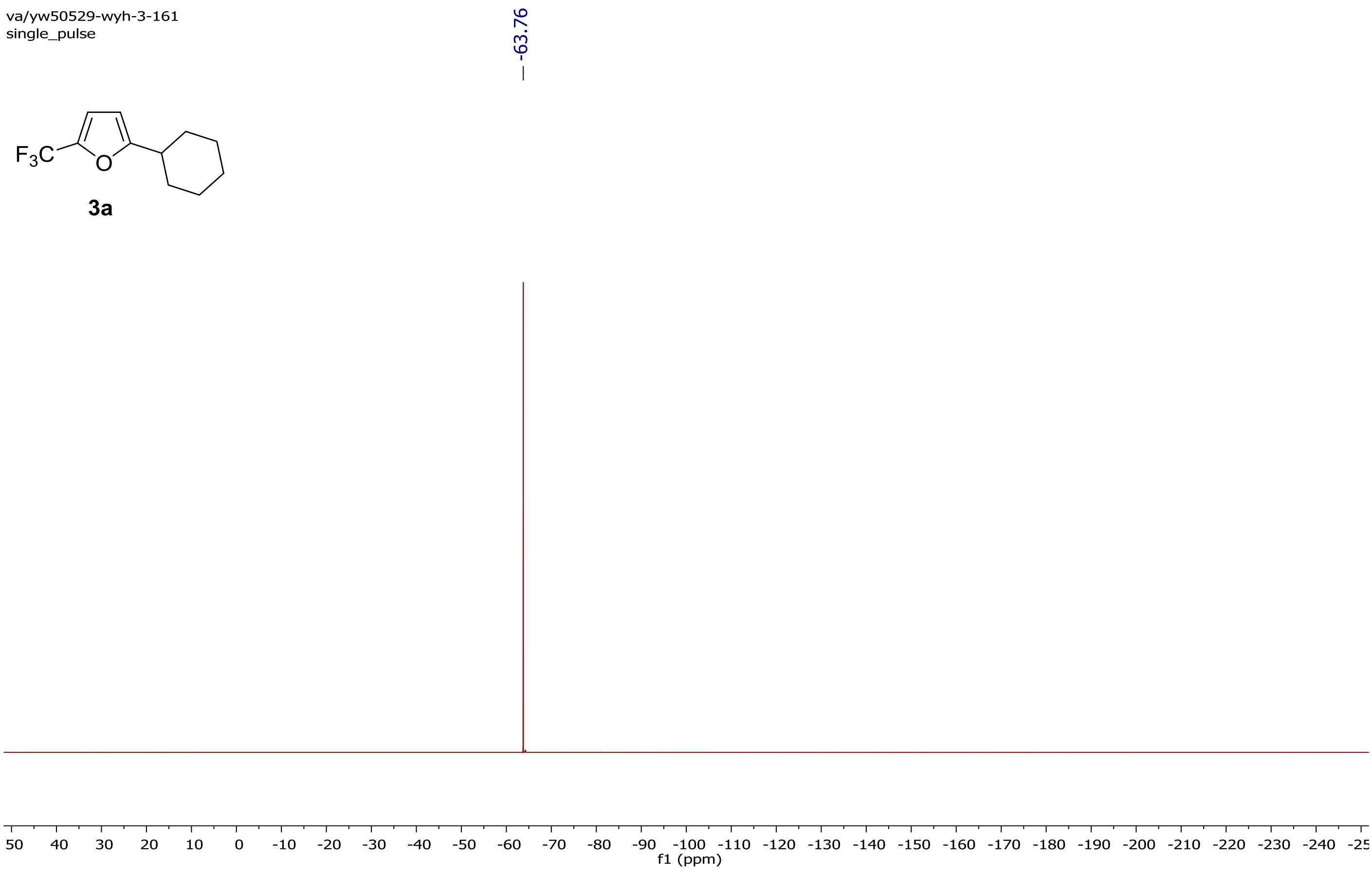
va/yw50529-wyh-3-161
single_pulse
-63.76
F3C
O
3a
50 40 30 20 10 0 -10 -20 -30 -40 -50 -60 -70 -80 -90 -100 -110 -120 -130 -140 -150 -160 -170 -180 -190 -200 -210 -220 -230 -240
f1 (ppm)

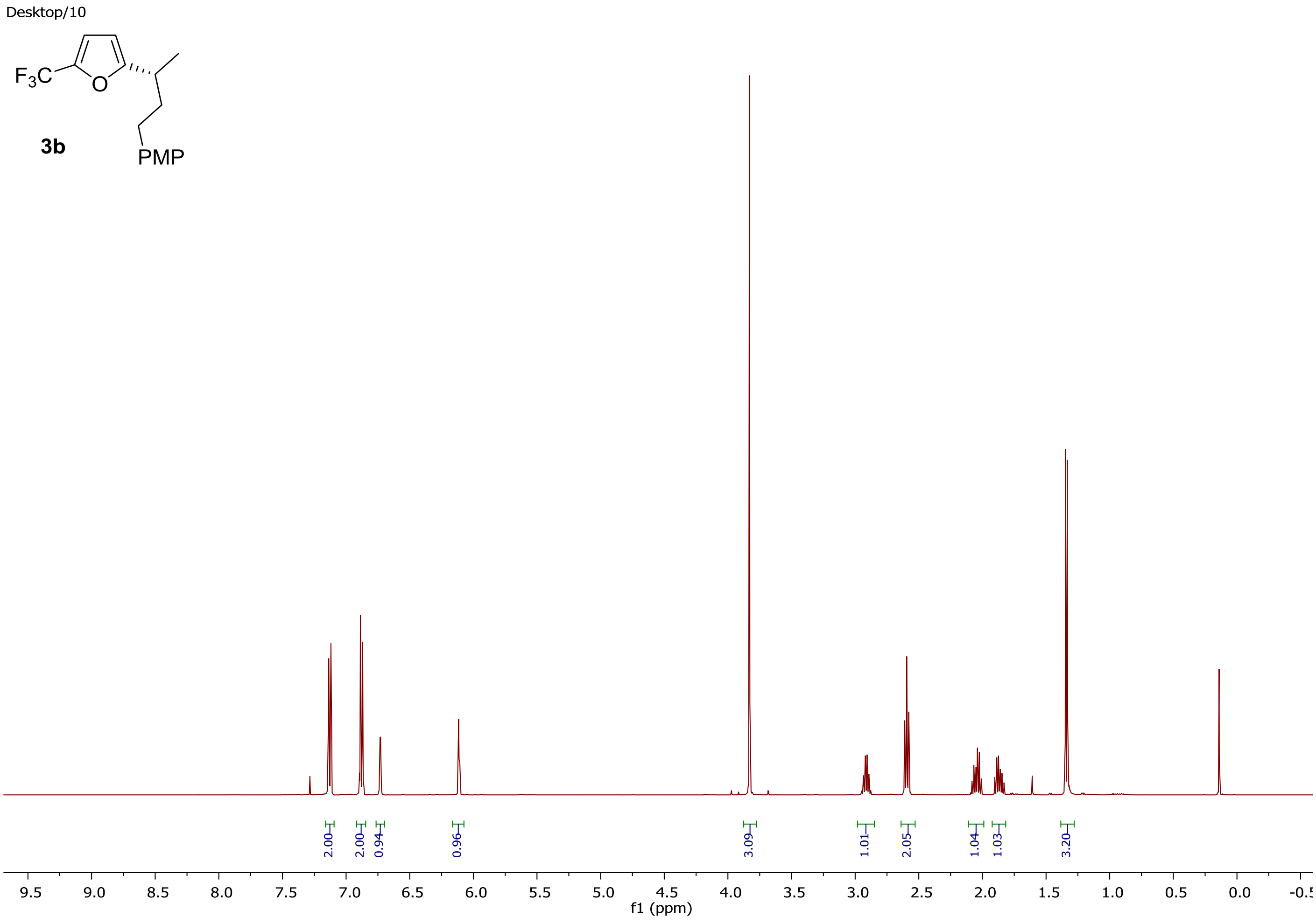
Desktop/10
F3C
O
3b
PMP
2.00 2.00 0.94 0.96 3.09 1.01 2.05 1.04 1.03 3.20
9.5 9.0 8.5 8.0 7.5 7.0 6.5 6.0 5.5 5.0 4.5 4.0 3.5 3.0 2.5 2.0 1.5 1.0 0.5 0.0
f1 (ppm)

Desktop/wyh-3-162-carbon

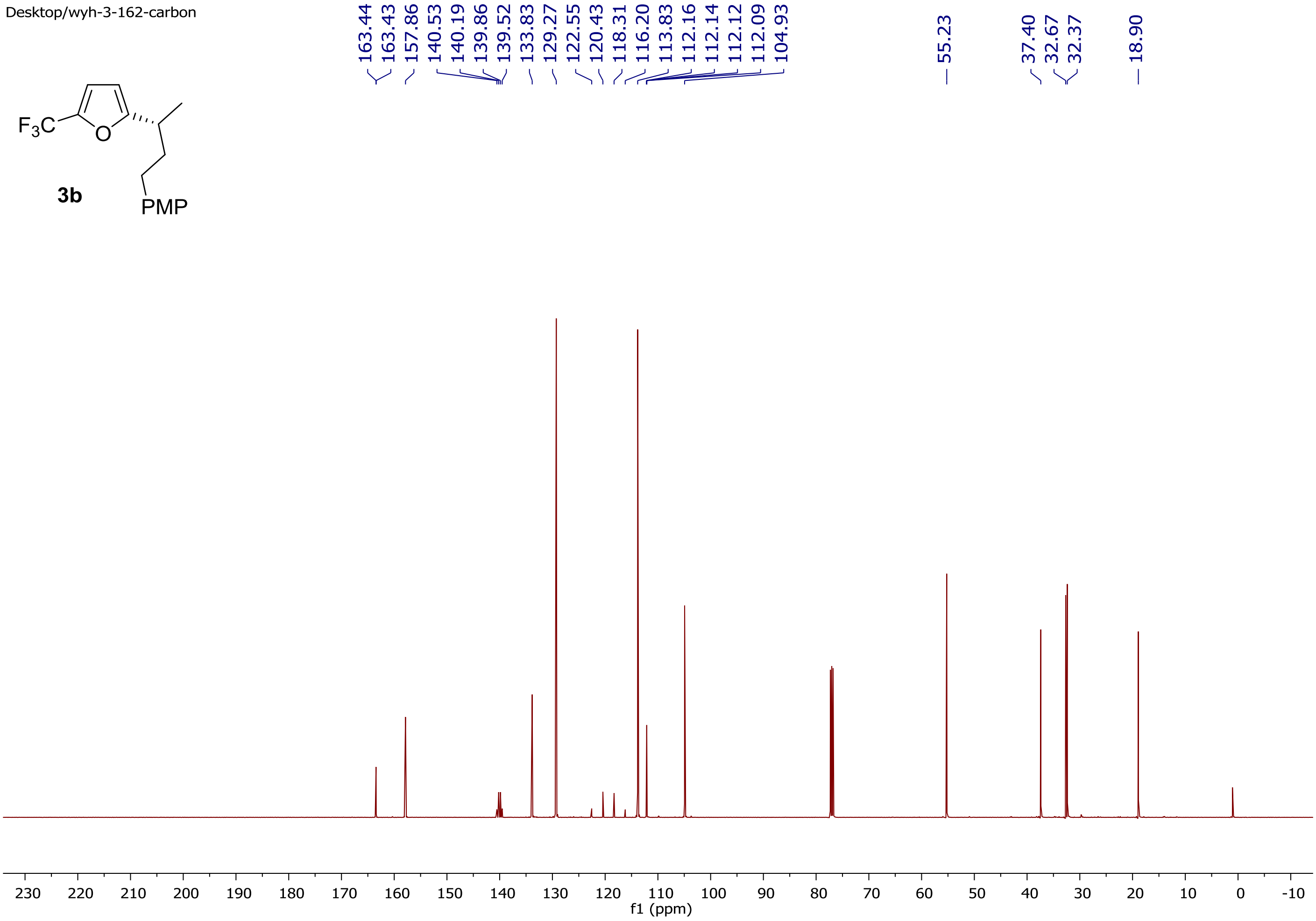

va/yw24623-wyh-3-162
single_pulse

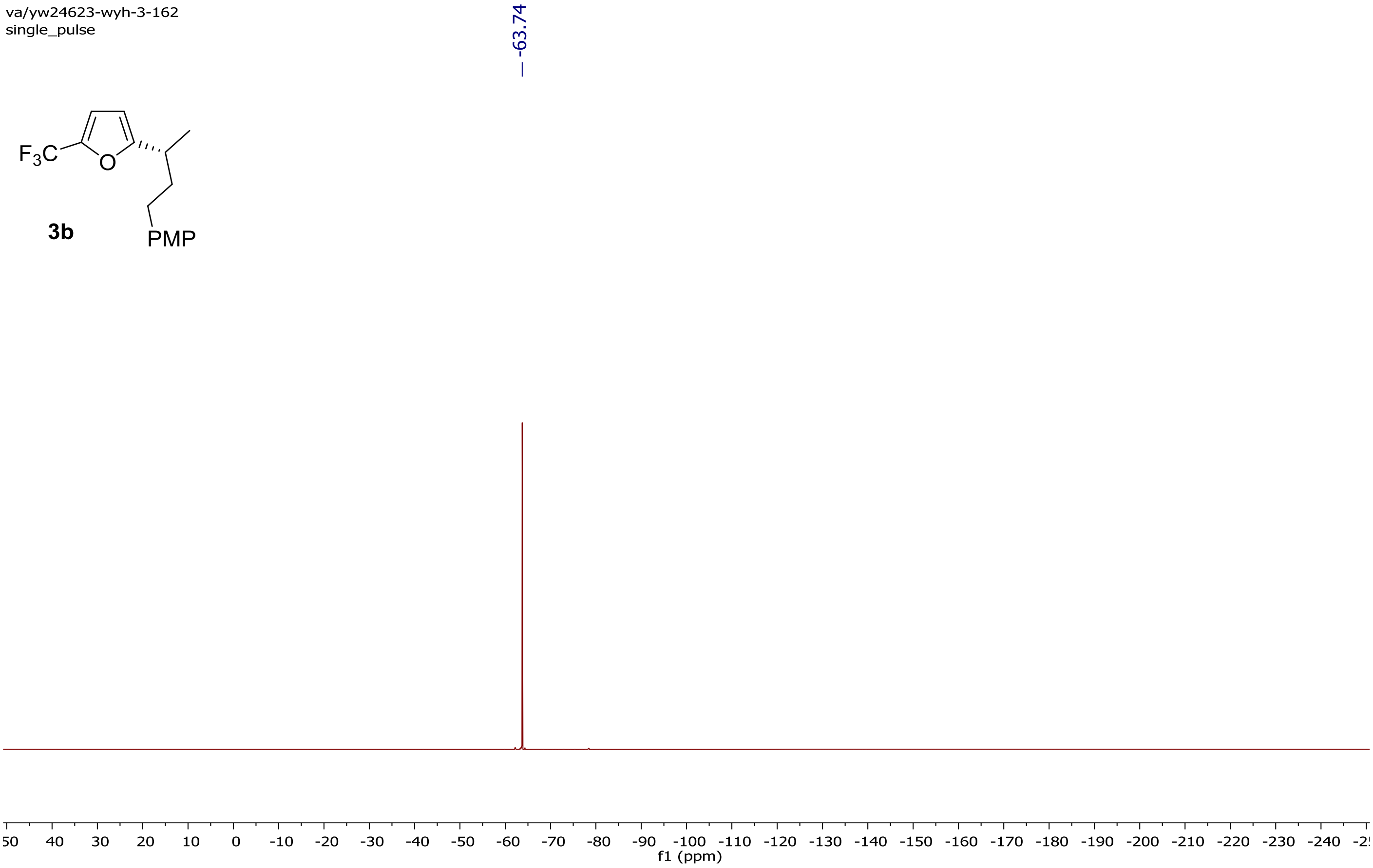

va/yw24751-wyh-3-166
single_pulse

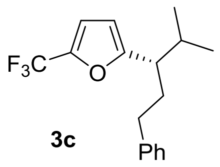

3c

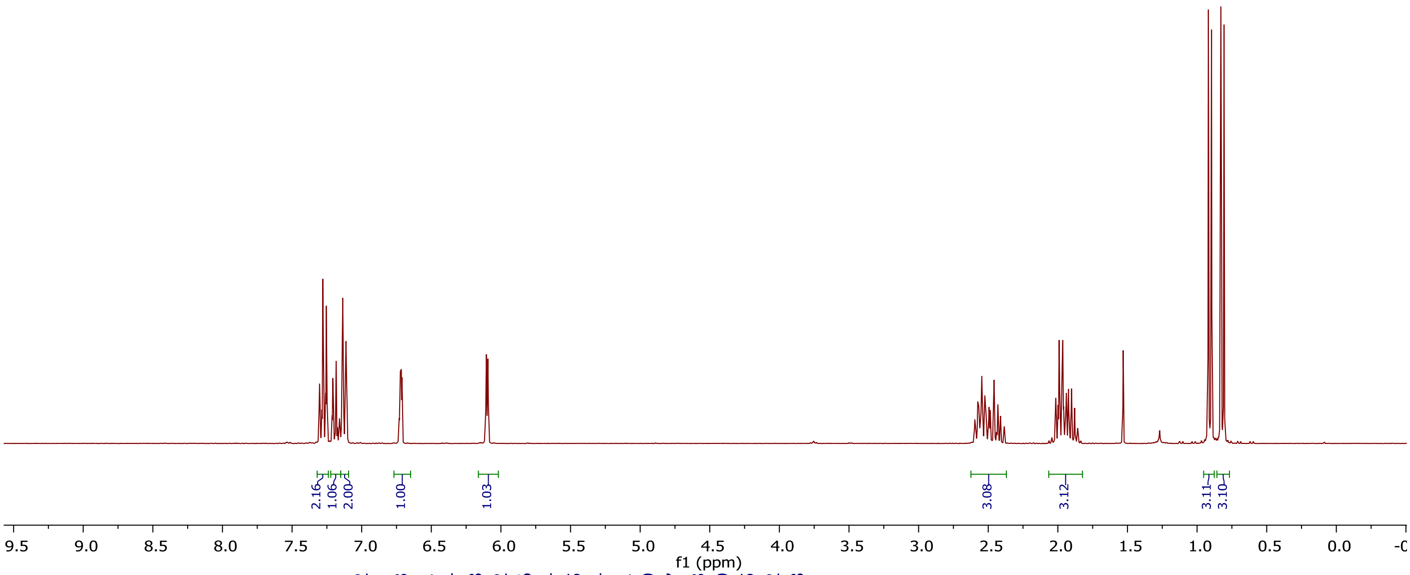

va/yw24751-wyh-3-166
single pulse decoupled gated NOE

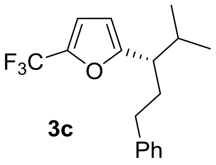

3c

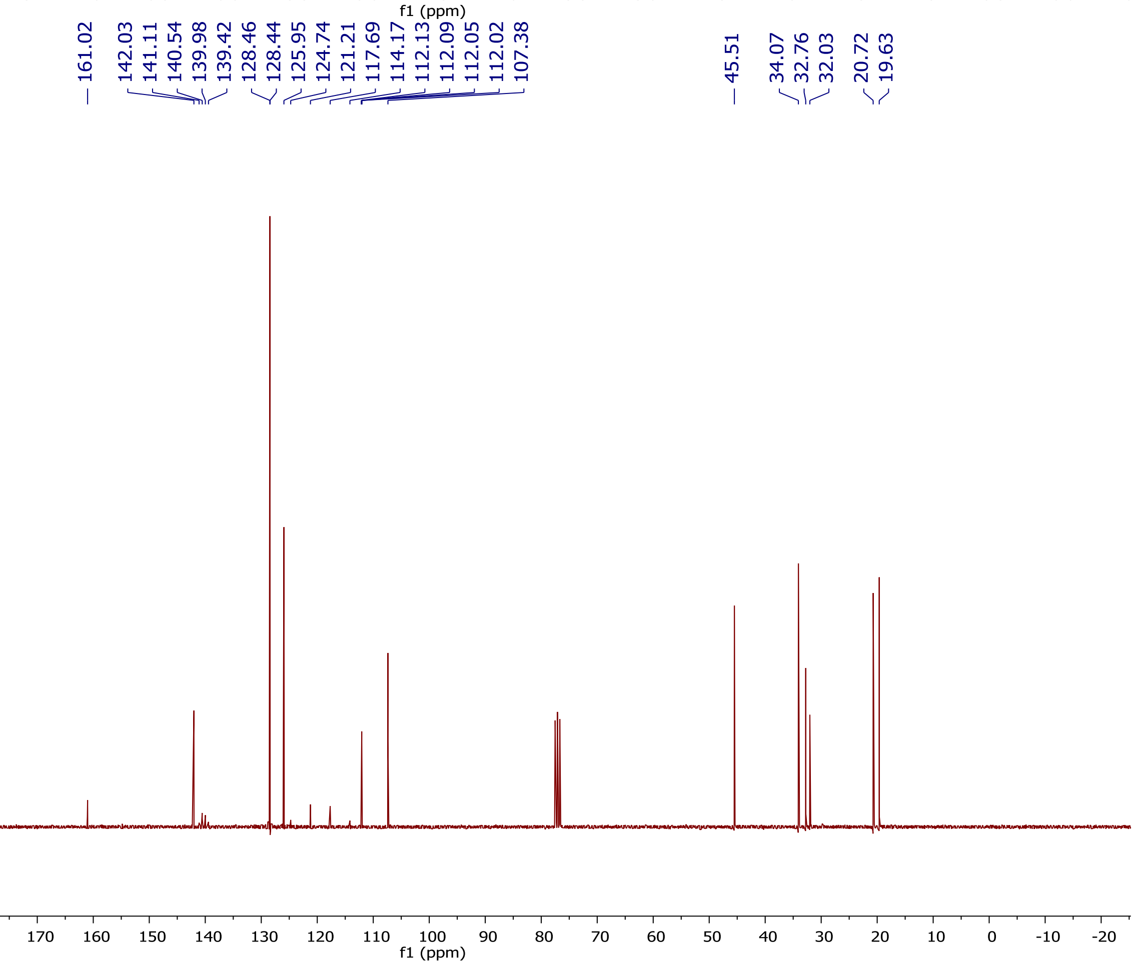

va/yw24751-wyh-3-166
single_pulse

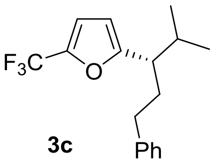

3c

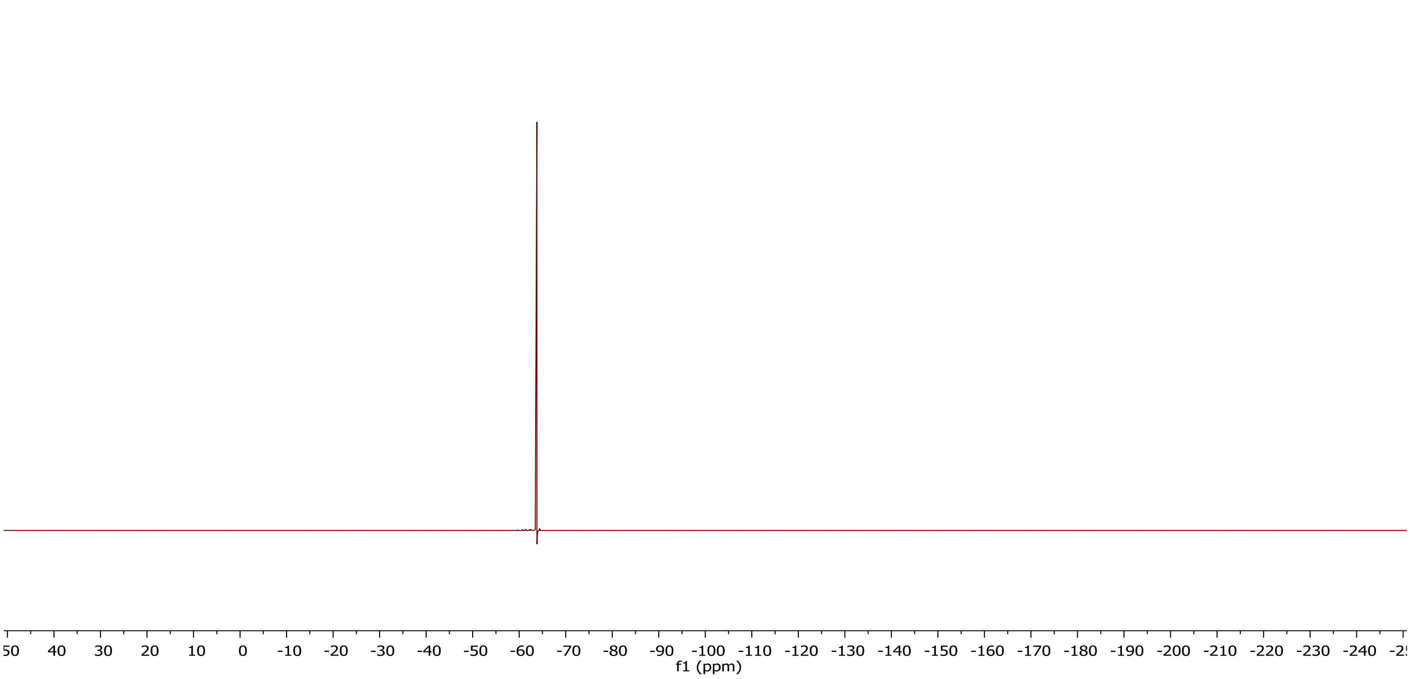

va/yw25217-wyh-3-188
single_pulse

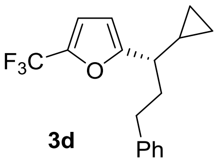

3d

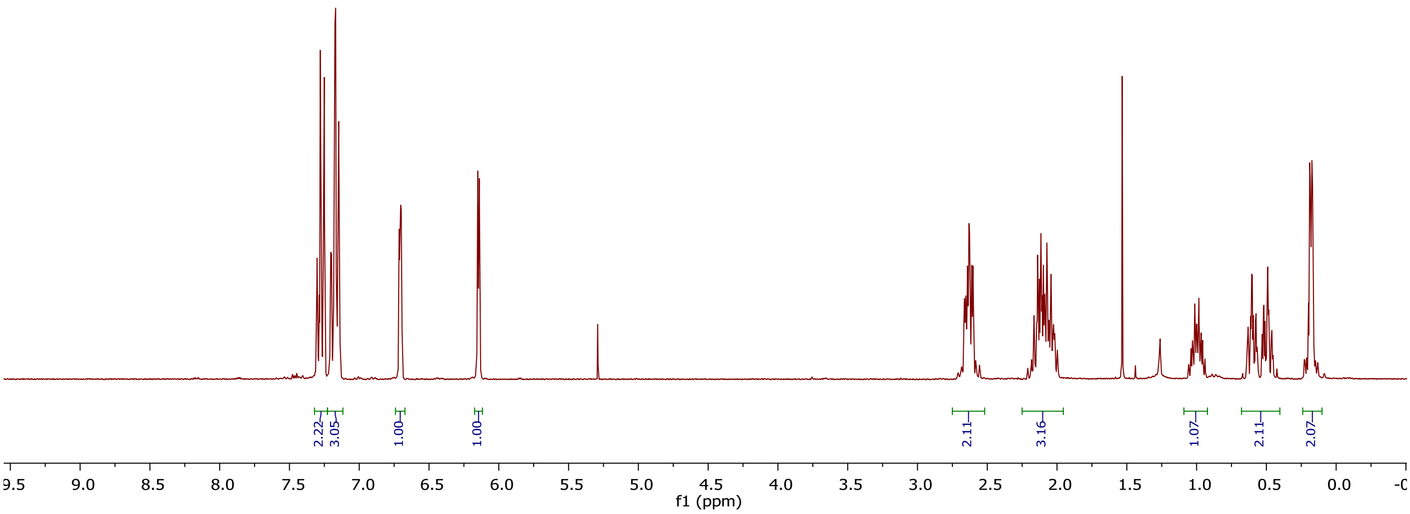

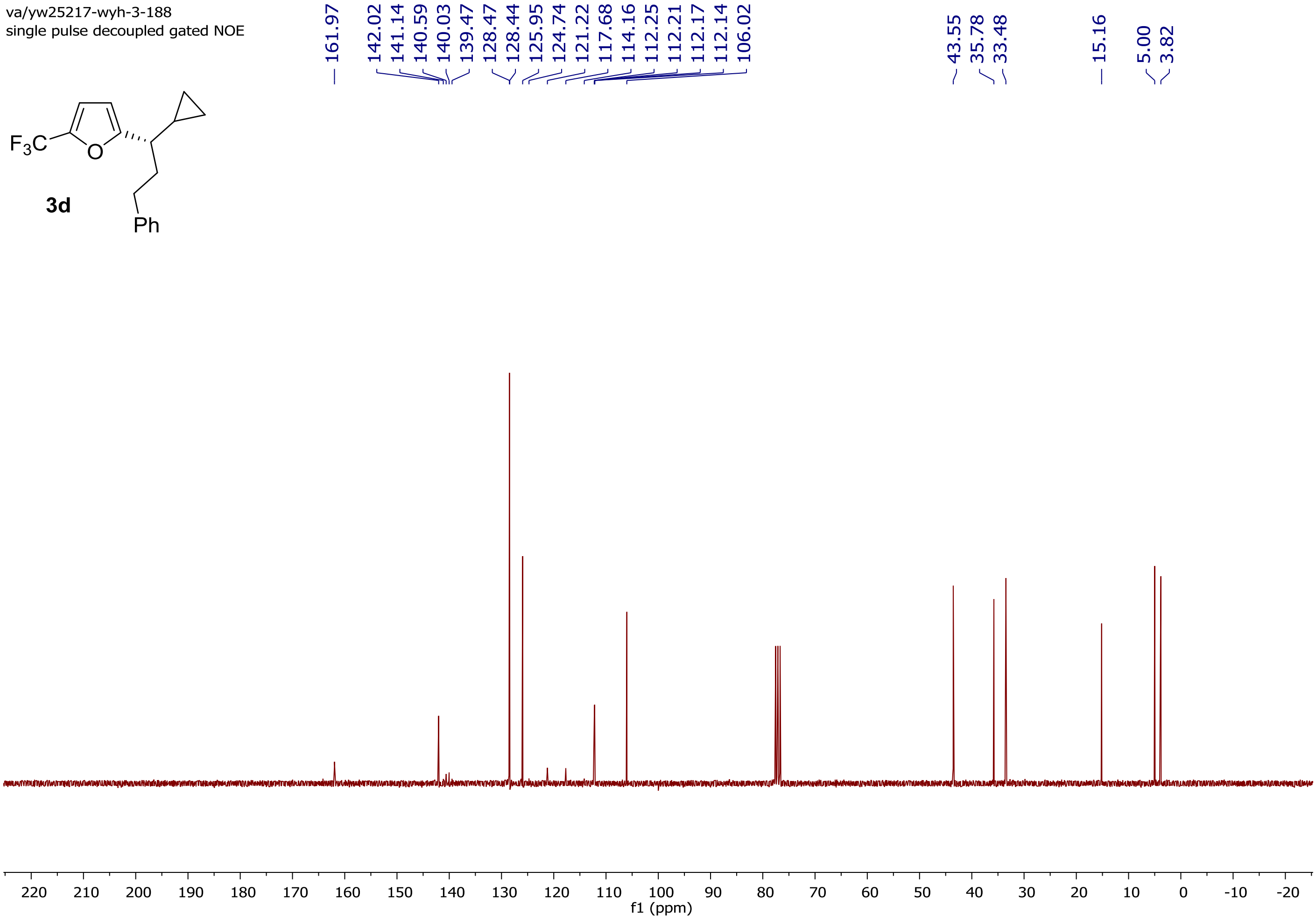
va/yw25217-wyh-3-188
single pulse decoupled gated NOE
161.97
142.02
141.14
140.59
140.03
139.47
128.47
128.44
125.95
124.74
121.22
117.68
114.16
112.25
112.21
112.17
112.14
106.02
43.55
35.78
33.48
15.16
5.00
3.82
F3C
O
Ph
3d
f1 (ppm)

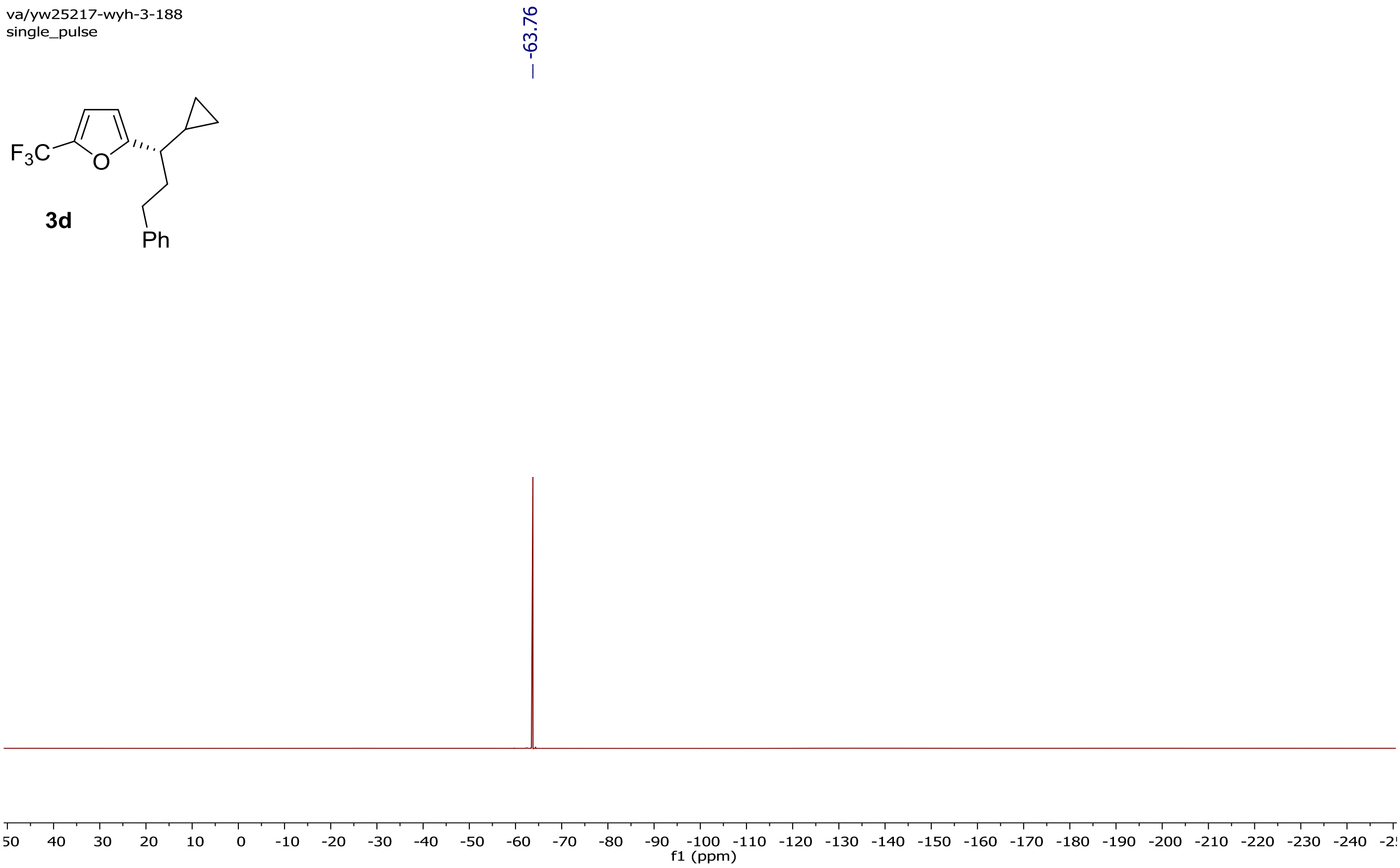
va/yw25217-wyh-3-188
single_pulse
-63.76
F3C
O
Ph
3d
f1 (ppm)

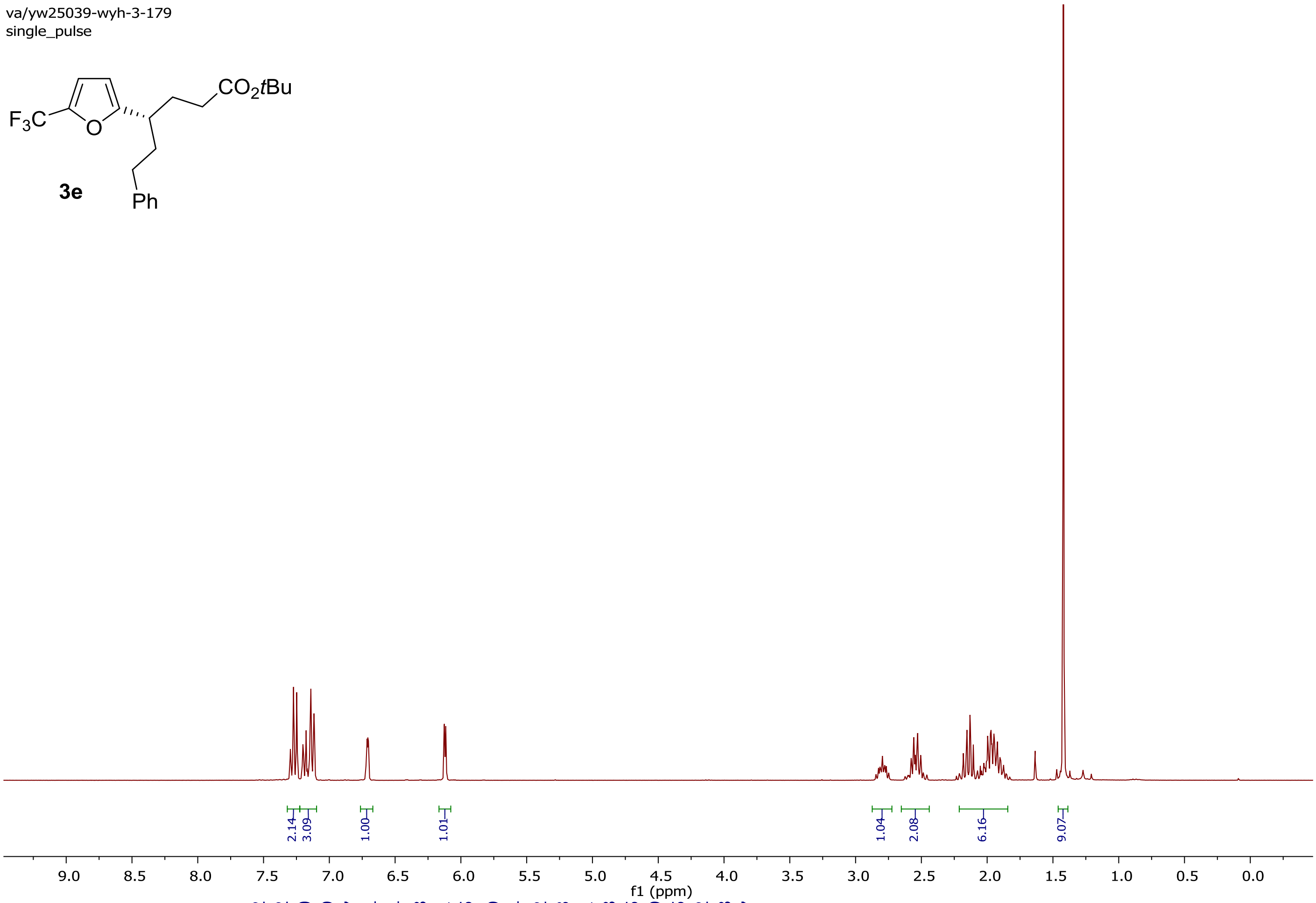
va/yw25039-wyh-3-179
single_pulse
CO_2tBu
F_3C
O
3e
Ph
2.14
3.09
1.00
1.01
1.04
2.08
6.16
9.07
9.0 8.5 8.0 7.5 7.0 6.5 6.0 5.5 5.0 4.5 4.0 3.5 3.0 2.5 2.0 1.5 1.0 0.5 0.0
f1 (ppm)

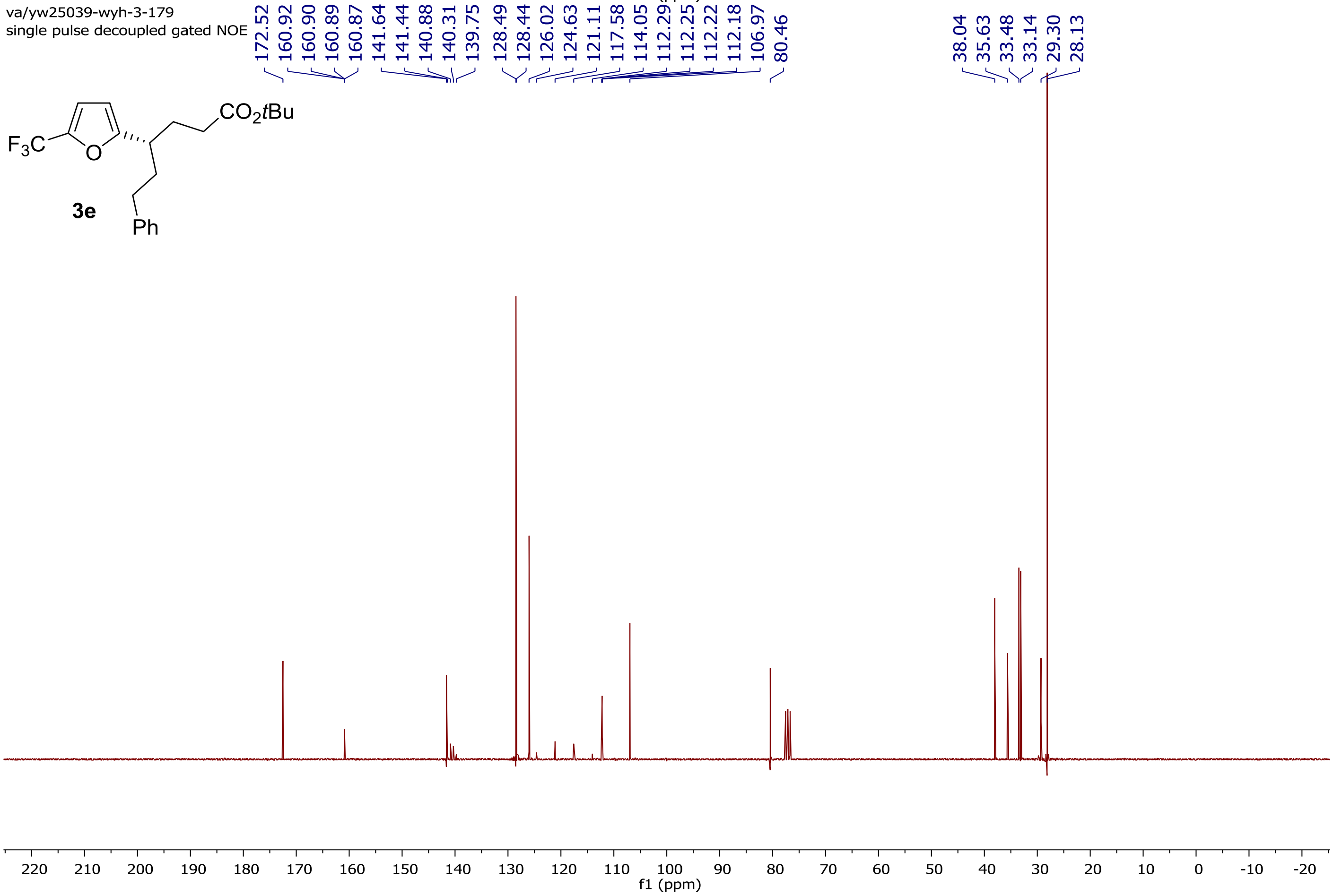
va/yw25039-wyh-3-179
single pulse decoupled gated NOE
172.52
160.92
160.90
160.89
160.87
141.64
141.44
140.88
140.31
139.75
128.49
128.44
126.02
124.63
121.11
117.58
114.05
112.29
112.25
112.22
112.18
106.97
80.46
38.04
35.63
33.48
33.14
29.30
28.13
CO_2tBu
F_3C
O
3e
Ph
220 210 200 190 180 170 160 150 140 130 120 110 100 90 80 70 60 50 40 30 20 10 0 -10 -20
f1 (ppm)

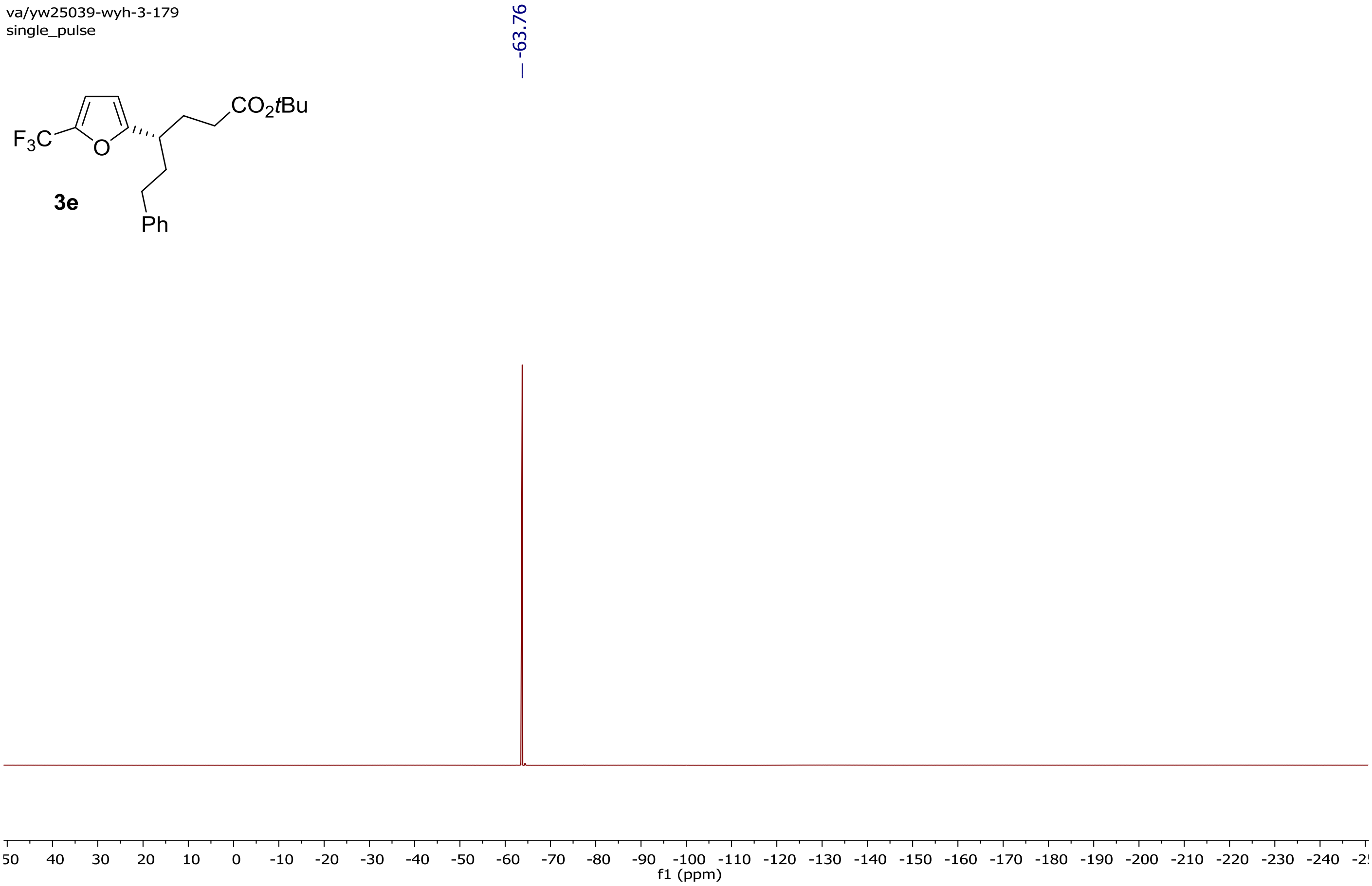
va/yw25039-wyh-3-179
single_pulse
-63.76
F_3C
O
CO_2tBu
Ph
3e
50 40 30 20 10 0 -10 -20 -30 -40 -50 -60 -70 -80 -90 -100 -110 -120 -130 -140 -150 -160 -170 -180 -190 -200 -210 -220 -230 -240
f1 (ppm)

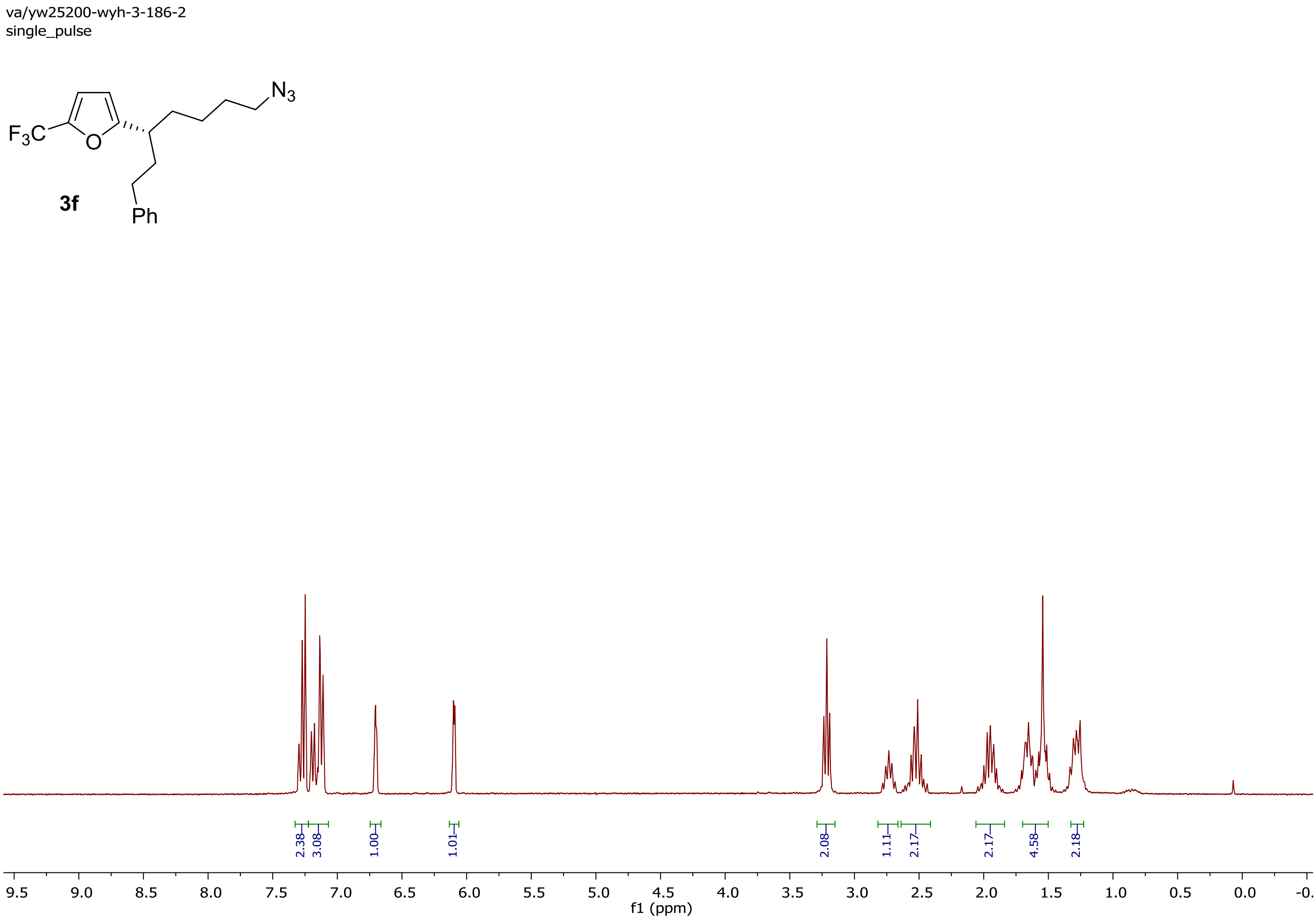
va/yw25200-wyh-3-186-2
single_pulse
F_3C
O
N_3
Ph
3f
2.38
3.08
1.00
1.01
2.08
1.11
2.17
2.17
4.58
2.18
9.5 9.0 8.5 8.0 7.5 7.0 6.5 6.0 5.5 5.0 4.5 4.0 3.5 3.0 2.5 2.0 1.5 1.0 0.5 0.0
f1 (ppm)

va/yw25200-wyh-3-186-2
single pulse decoupled gated NOE

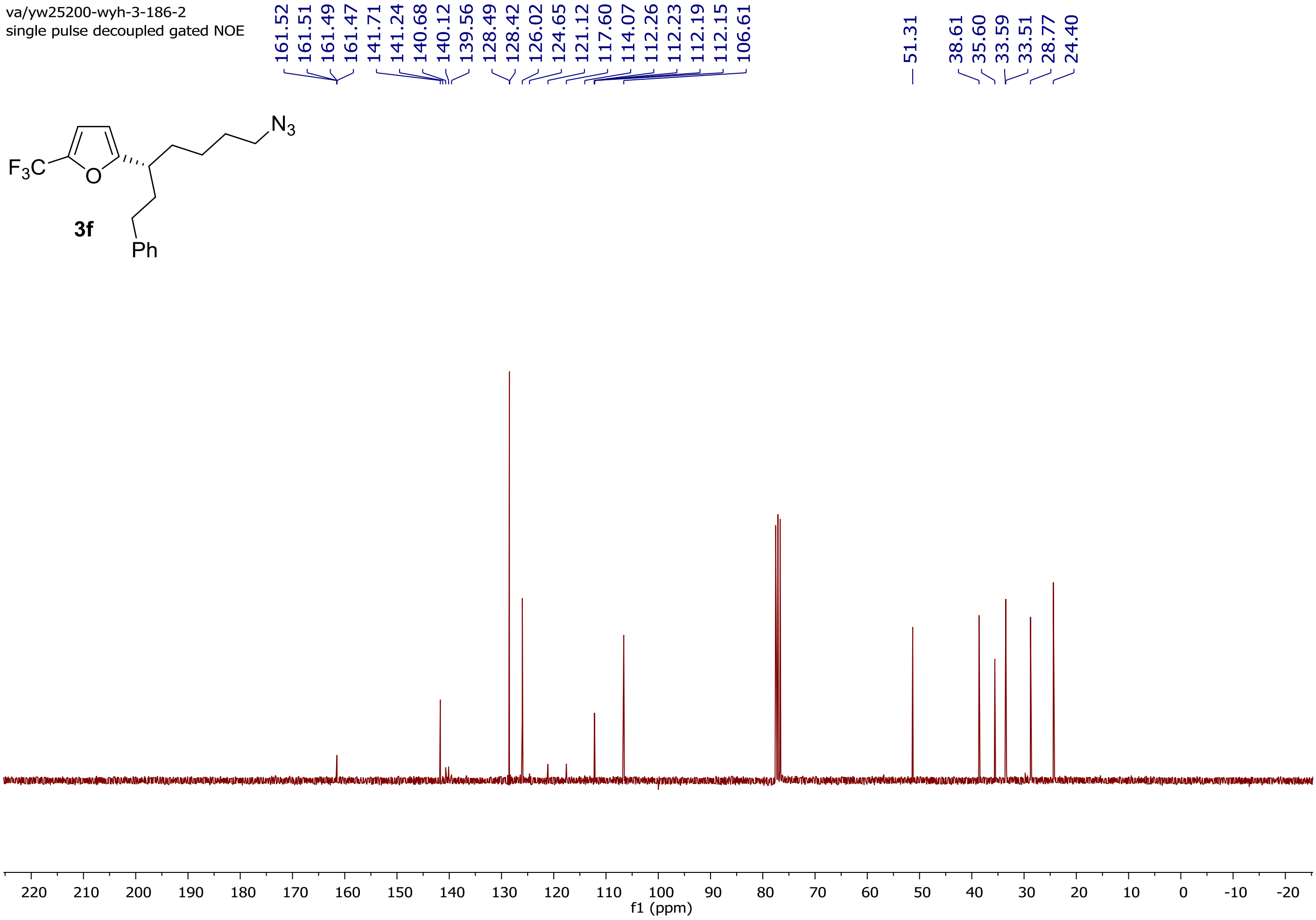

va/yw25200-wyh-3-186-2
single_pulse

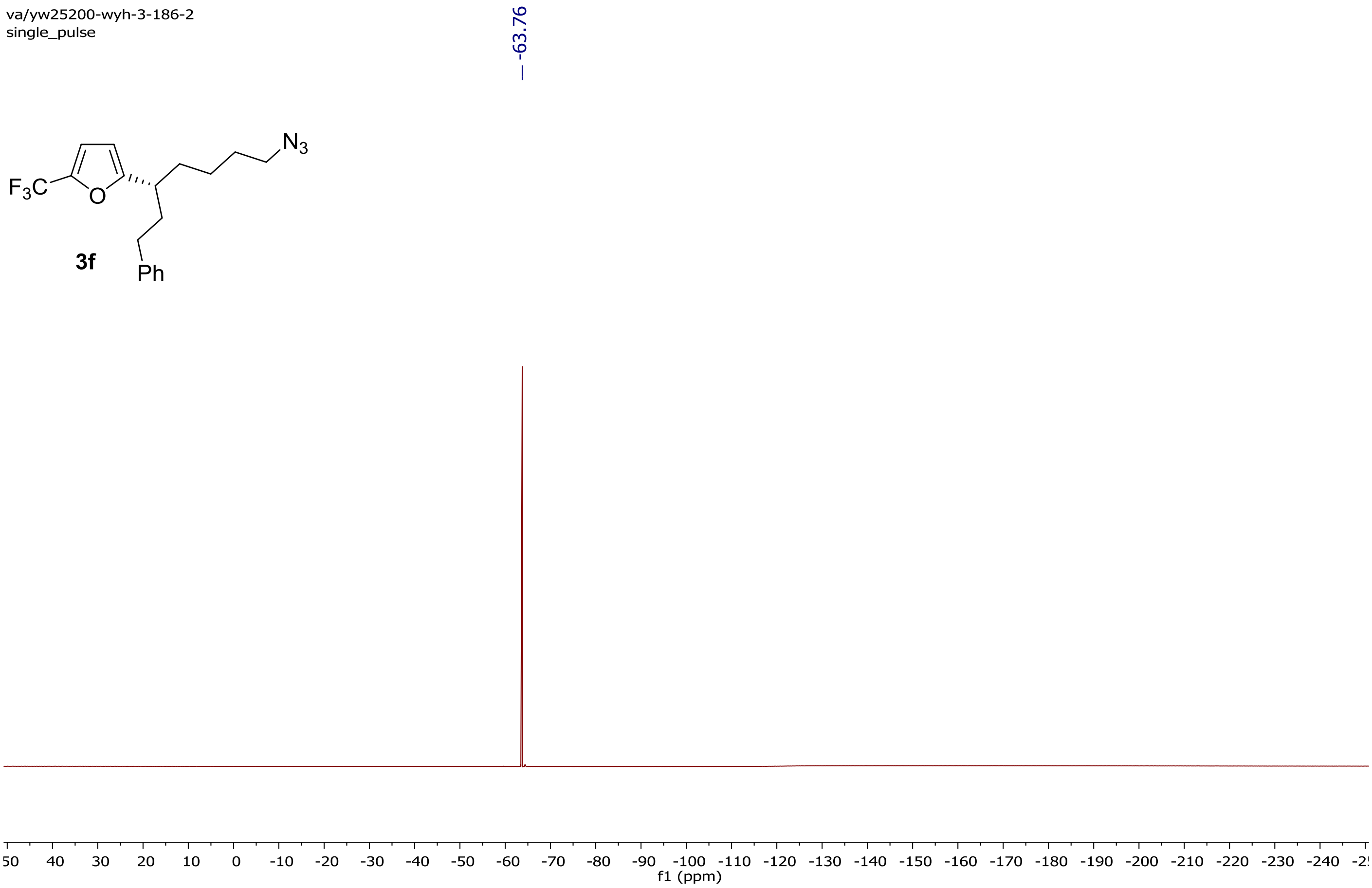

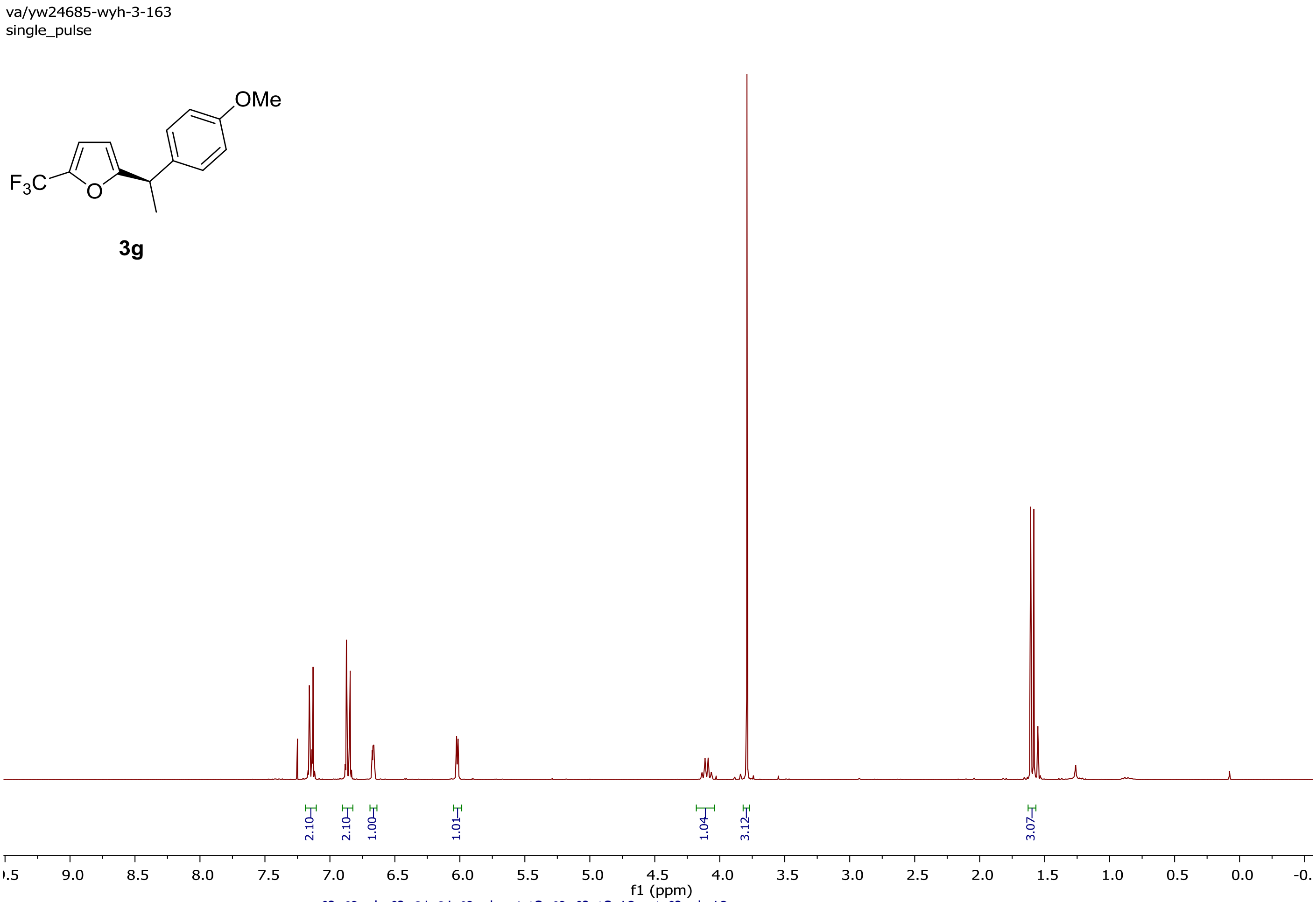
va/yw24685-wyh-3-163
single_pulse
OMe
F3C
O
3g
2.10
2.10
1.00
1.01
1.04
3.12
3.07
f1 (ppm)

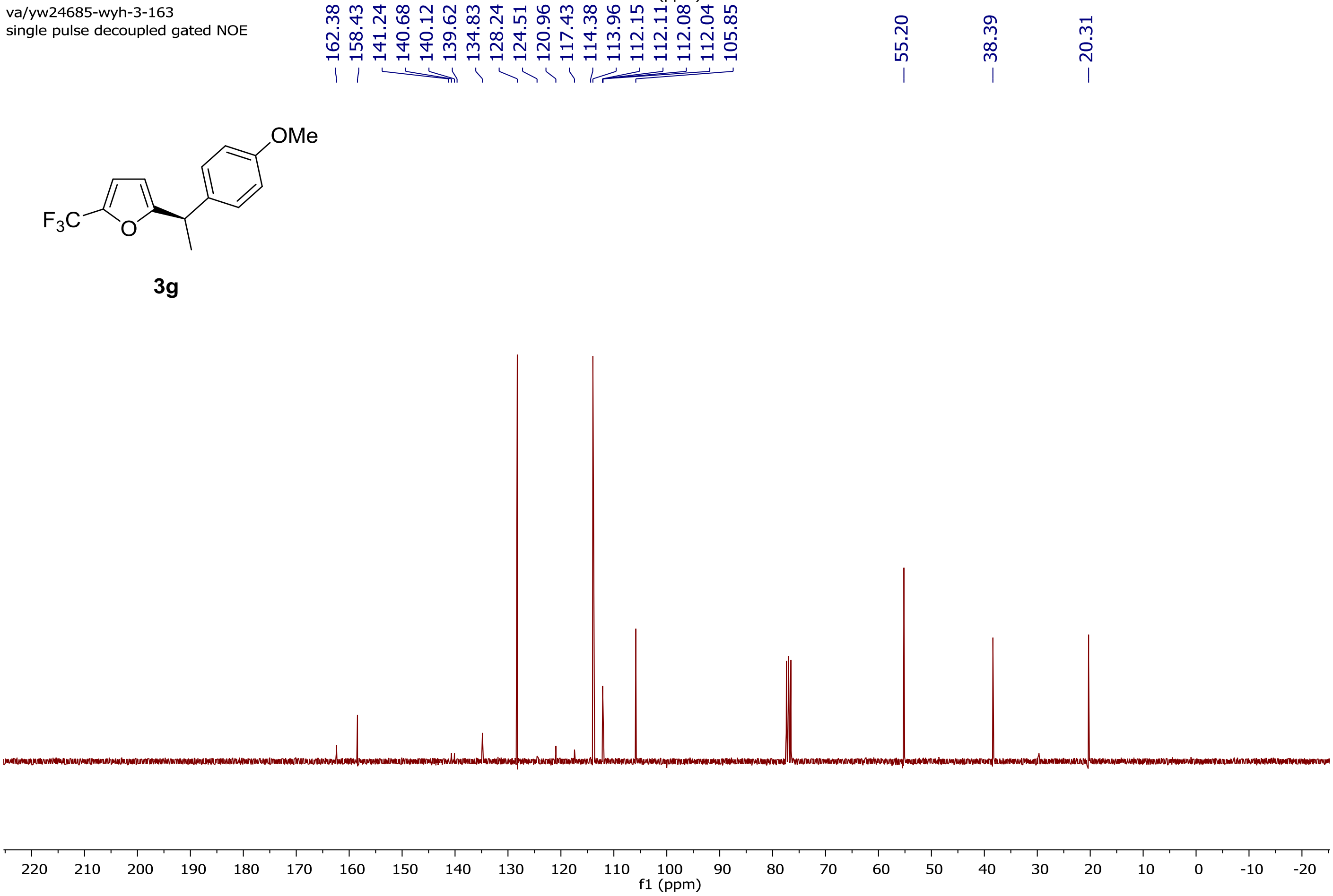
va/yw24685-wyh-3-163
single pulse decoupled gated NOE
162.38
158.43
141.24
140.68
140.12
139.62
134.83
128.24
124.51
120.96
117.43
114.38
113.96
112.15
112.11
112.08
112.04
105.85
55.20
38.39
20.31
OMe
F3C
O
3g
f1 (ppm)

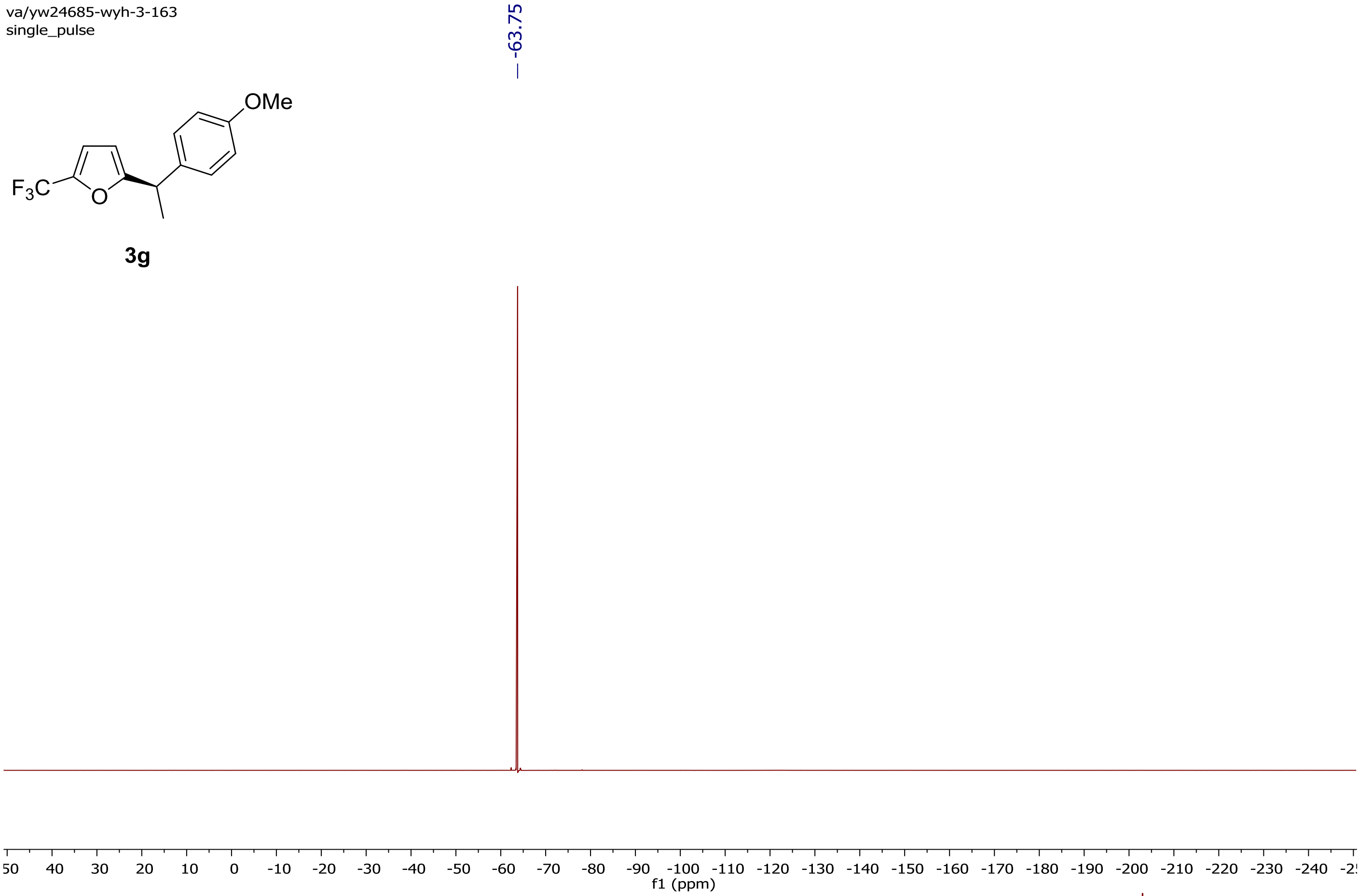
va/yw24685-wyh-3-163
single_pulse
-63.75
OMe
F3C
O
f1 (ppm)

3g

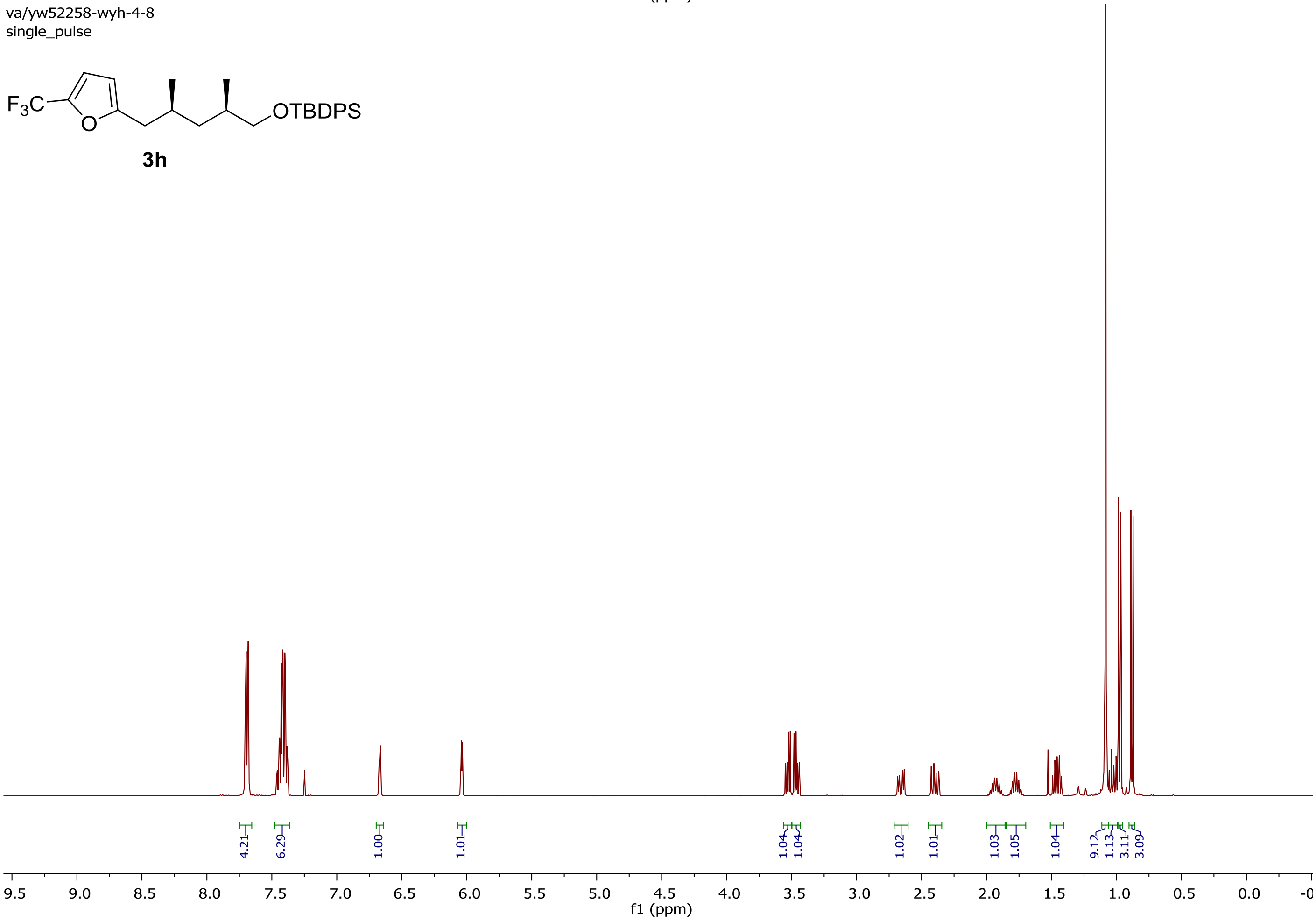
va/yw52258-wyh-4-8
single_pulse
F3C
O
OTBDPS
4.21
6.29
1.00
1.01
1.04
1.04
1.02
1.01
1.03
1.05
1.04
9.12
1.13
3.11
3.09
f1 (ppm)

3h

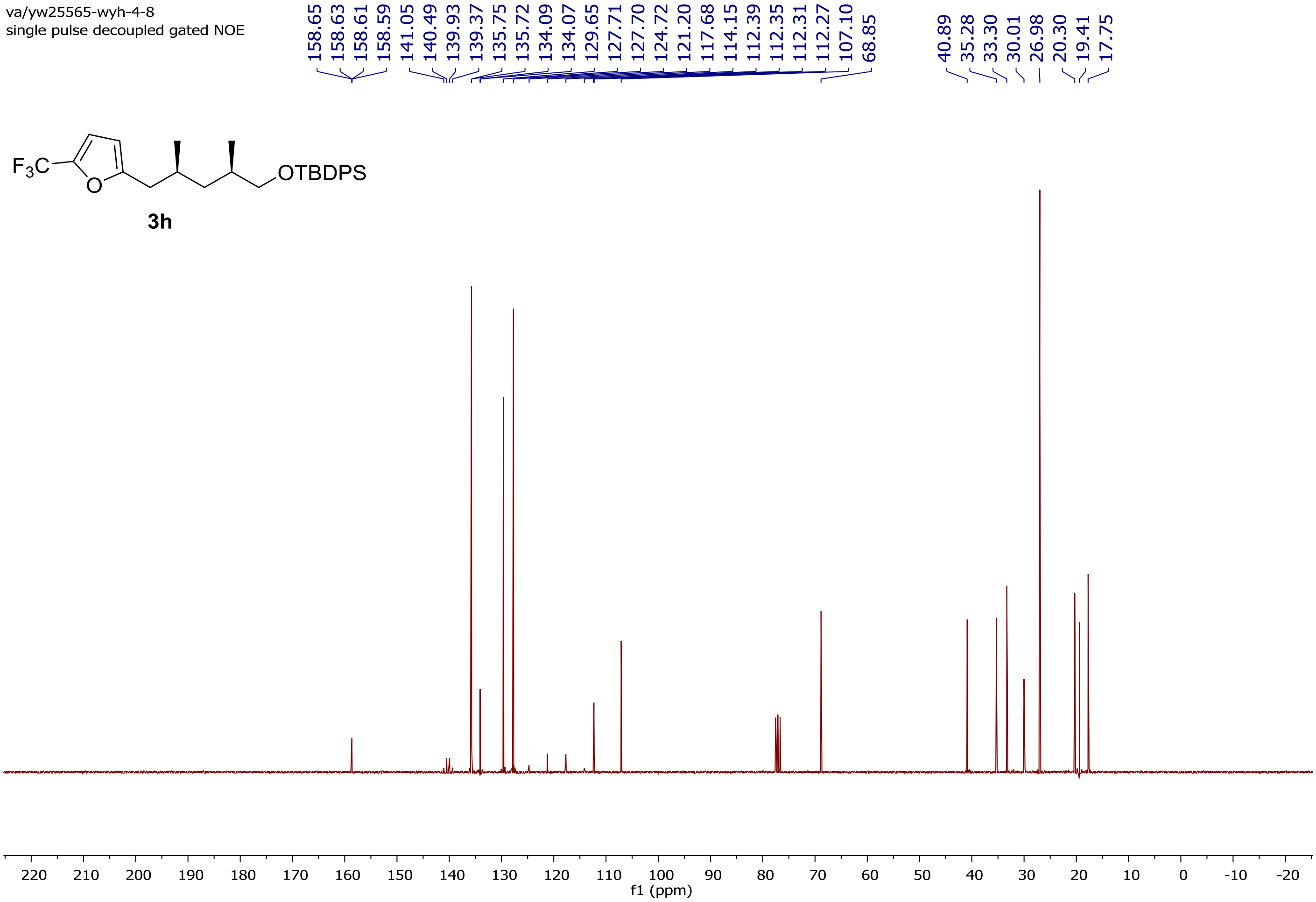
va/yw25565-wyh-4-8
single pulse decoupled gated NOE
158.65 158.63 158.61 158.59 141.05 140.49 139.93 139.37 135.75 135.72 134.09 134.07 129.65 127.71 127.70 124.72 121.20 117.68 114.15 112.39 112.35 112.31 112.27 107.10 68.85
40.89 35.28 33.30 30.01 26.98 20.30 19.41 17.75
F3C
O
OTBDPS
3h
220 210 200 190 180 170 160 150 140 130 120 110 100 90 80 70 60 50 40 30 20 10 0 -10 -20
f1 (ppm)

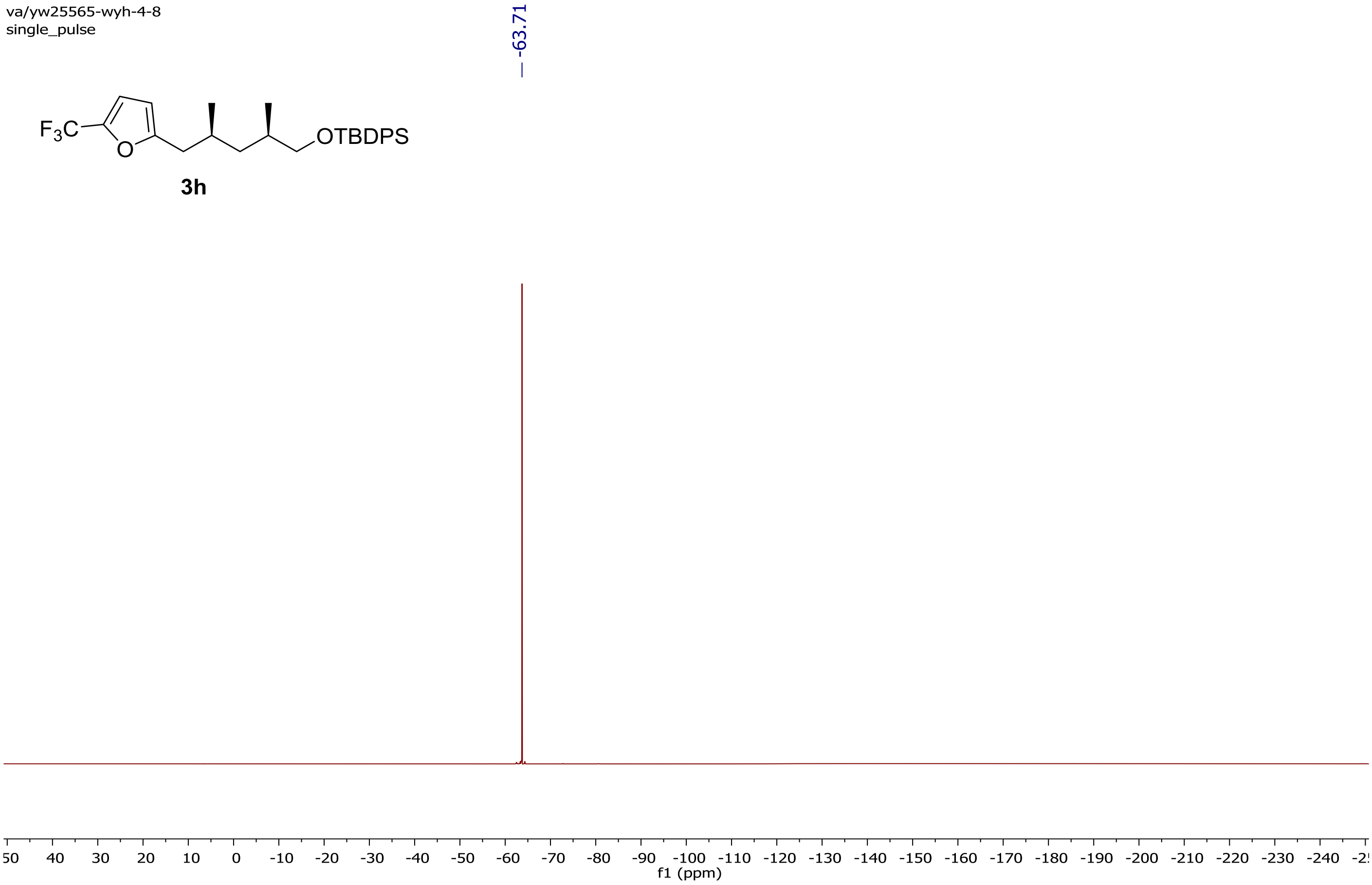
va/yw25565-wyh-4-8
single_pulse
-63.71
F3C
O
OTBDPS
3h
50 40 30 20 10 0 -10 -20 -30 -40 -50 -60 -70 -80 -90 -100 -110 -120 -130 -140 -150 -160 -170 -180 -190 -200 -210 -220 -230 -240
f1 (ppm)

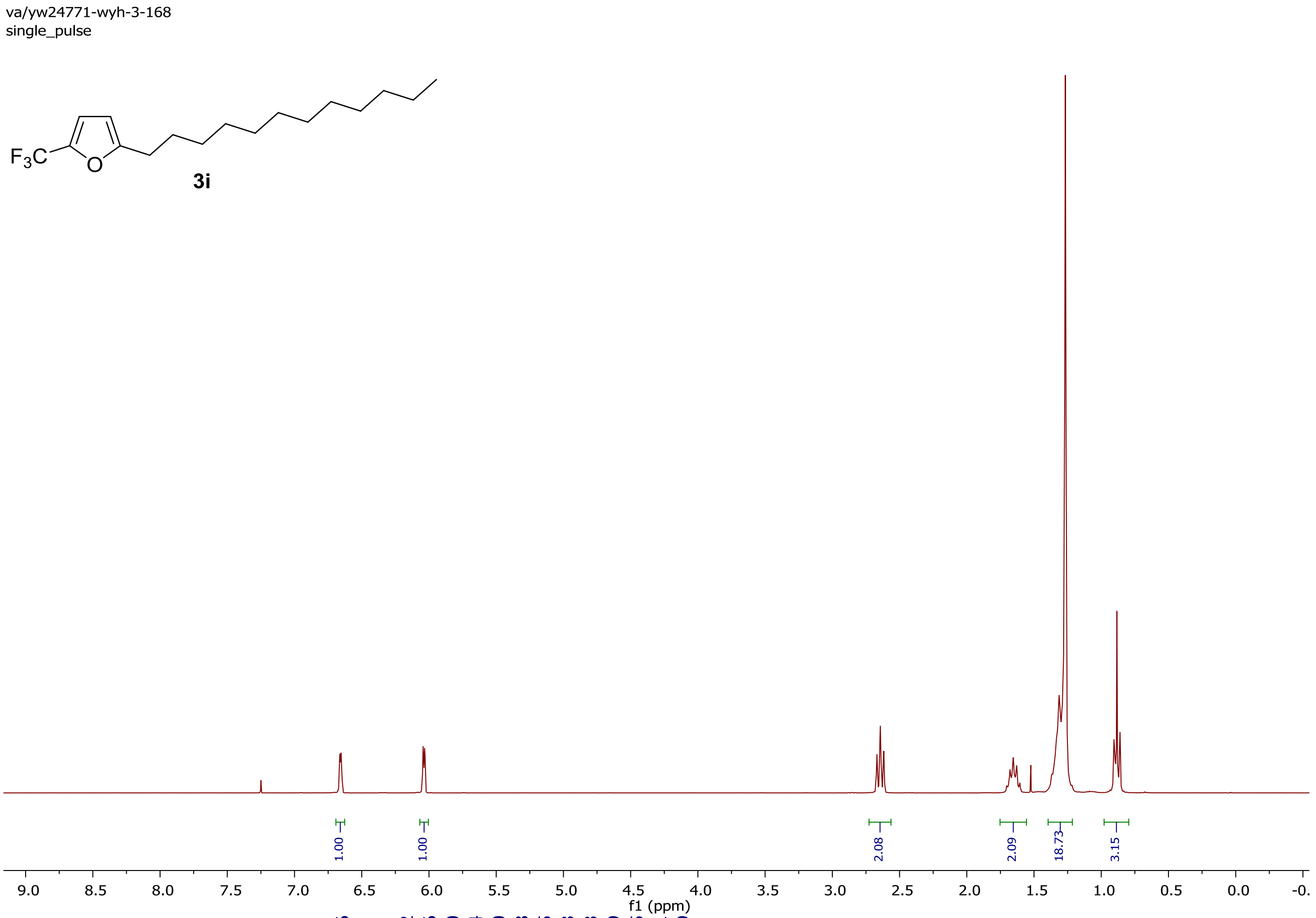
va/yw24771-wyh-3-168
single_pulse
F3C
O
3i
1.00
1.00
2.08
2.09
18.73
3.15
f1 (ppm)

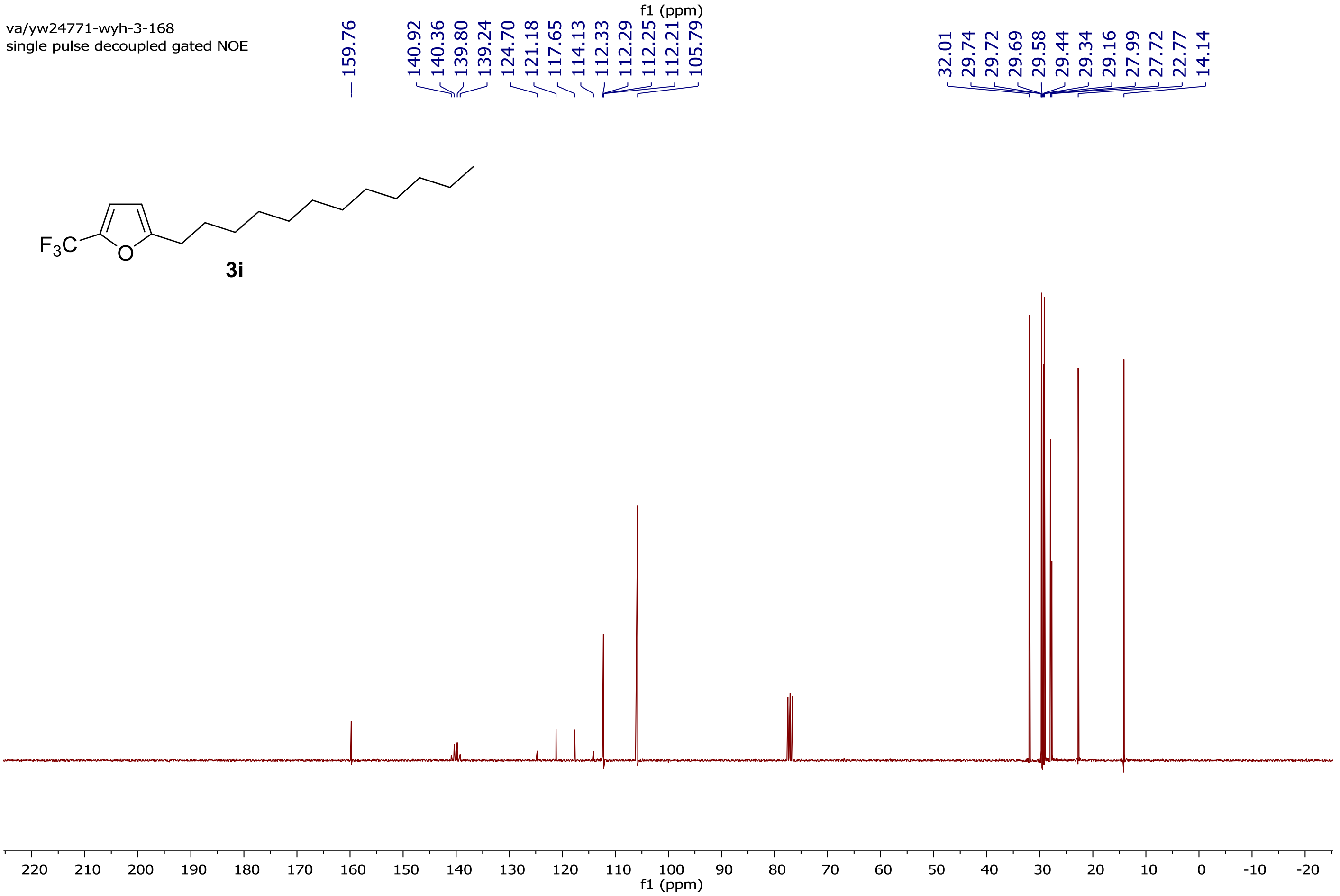
va/yw24771-wyh-3-168
single pulse decoupled gated NOE
159.76
140.92
140.36
139.80
139.24
124.70
121.18
117.65
114.13
112.33
112.29
112.25
112.21
105.79
32.01
29.74
29.72
29.69
29.58
29.44
29.34
29.16
27.99
27.72
22.77
14.14
F3C
O
3i
f1 (ppm)

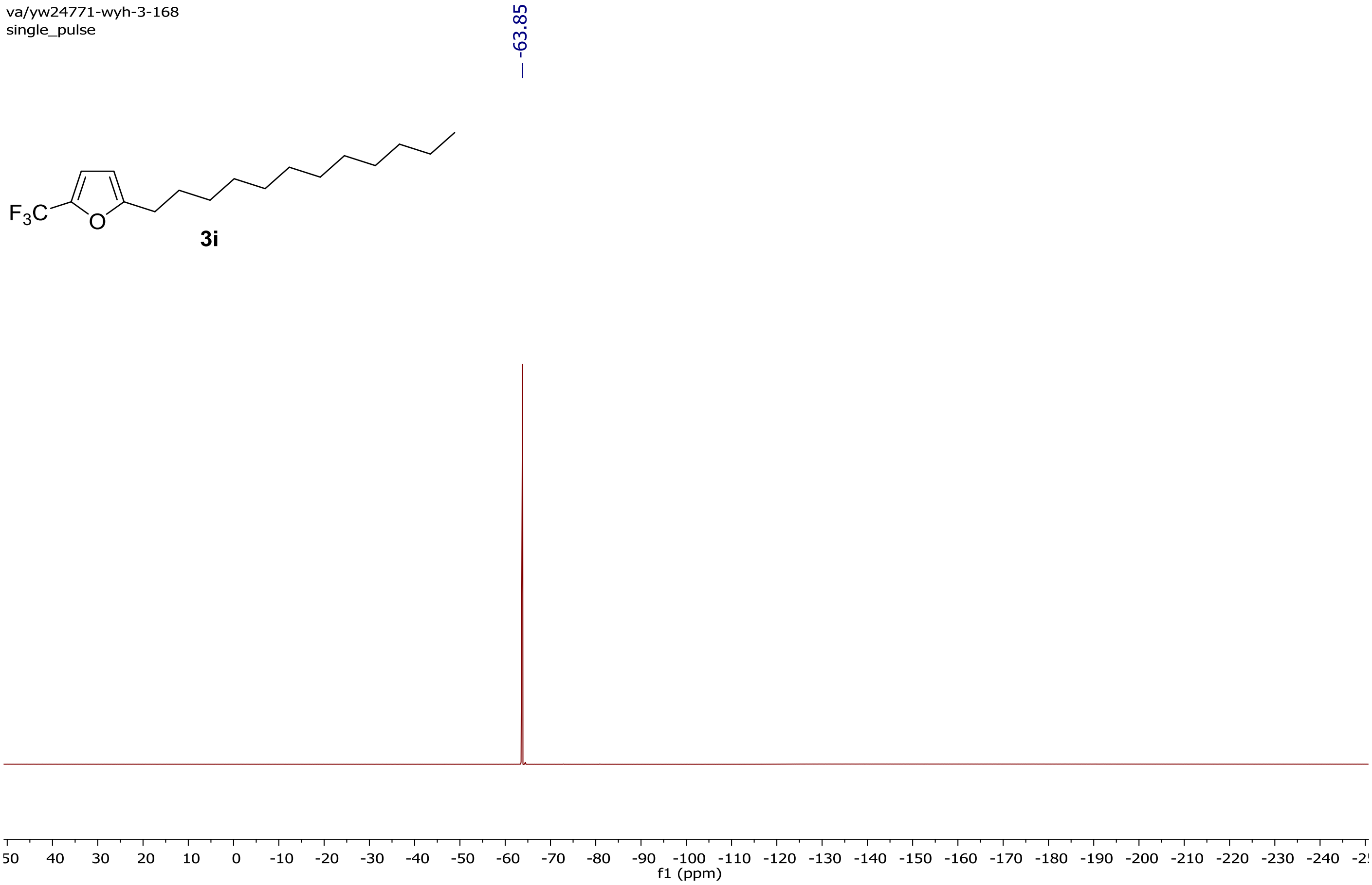
va/yw24771-wyh-3-168
single_pulse
-63.85
F_3C
O
3i
f1 (ppm)

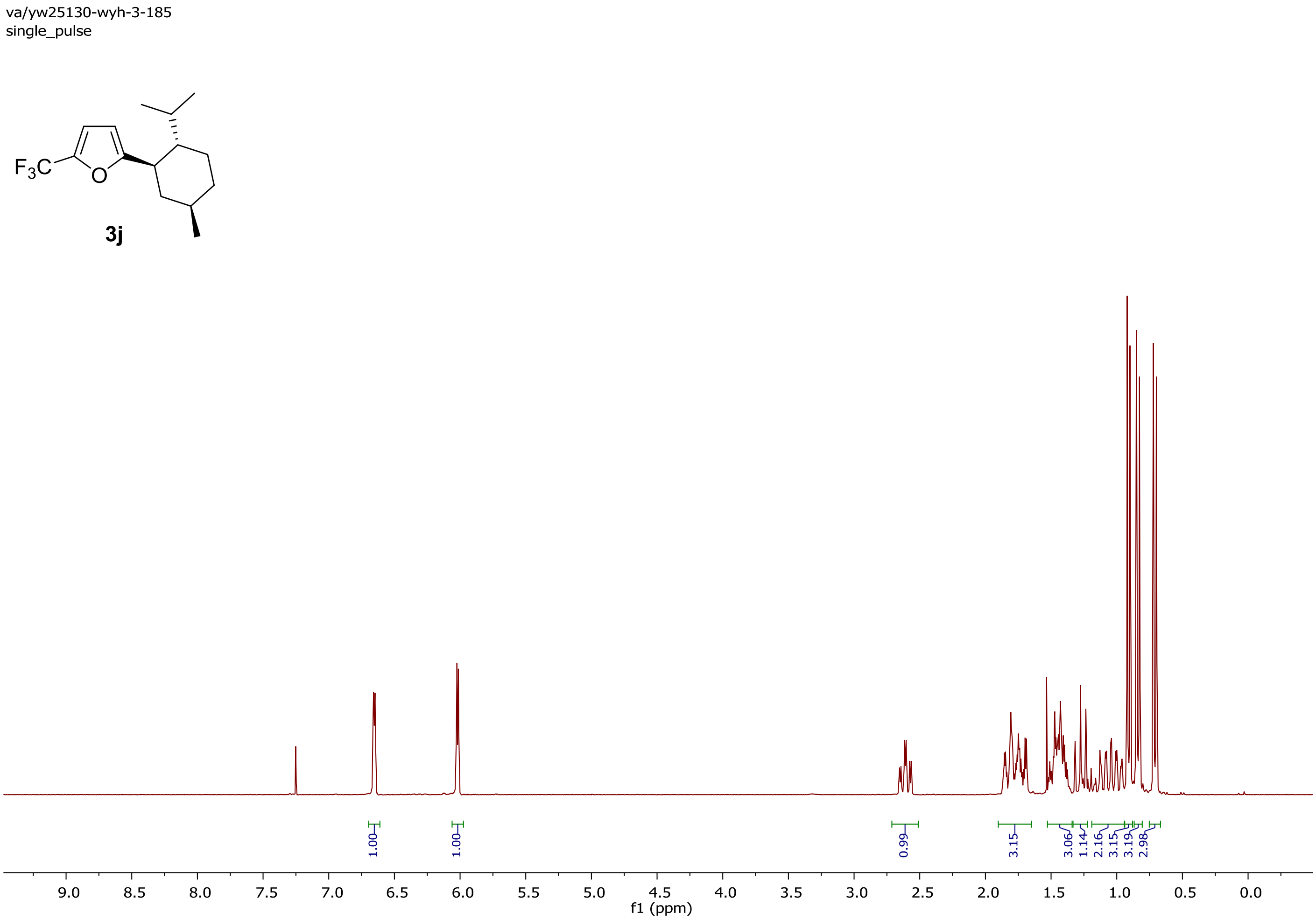
va/yw25130-wyh-3-185
single_pulse
F_3C
O
3j
1.00
1.00
0.99
3.15
3.06
1.14
2.16
3.15
3.19
2.98
f1 (ppm)

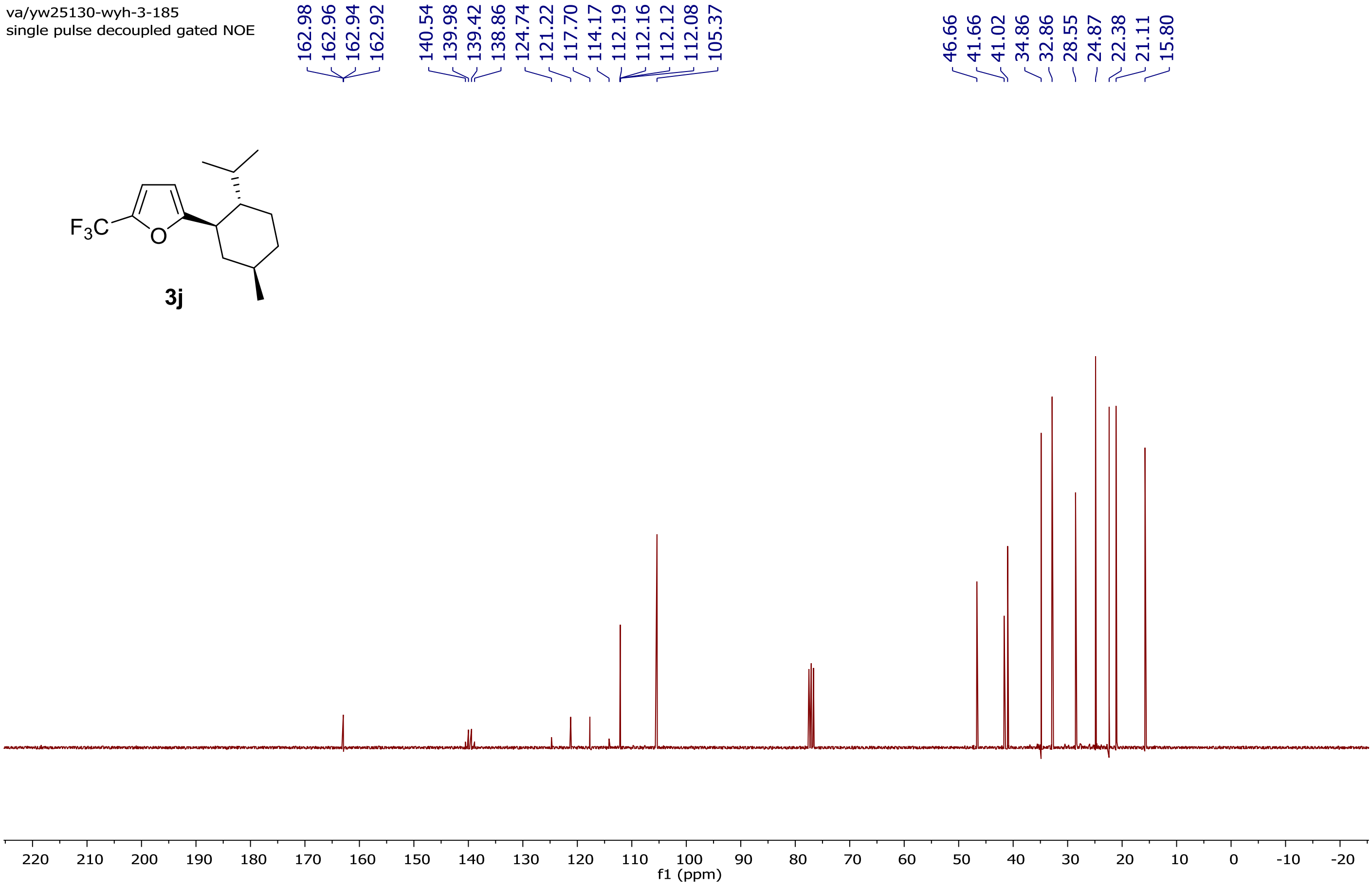
va/yw25130-wyh-3-185
single pulse decoupled gated NOE
162.98
162.96
162.94
162.92
140.54
139.98
139.42
138.86
124.74
121.22
117.70
114.17
112.19
112.16
112.12
112.08
105.37
46.66
41.66
41.02
34.86
32.86
28.55
24.87
22.38
21.11
15.80
F3C
O
3j
f1 (ppm)

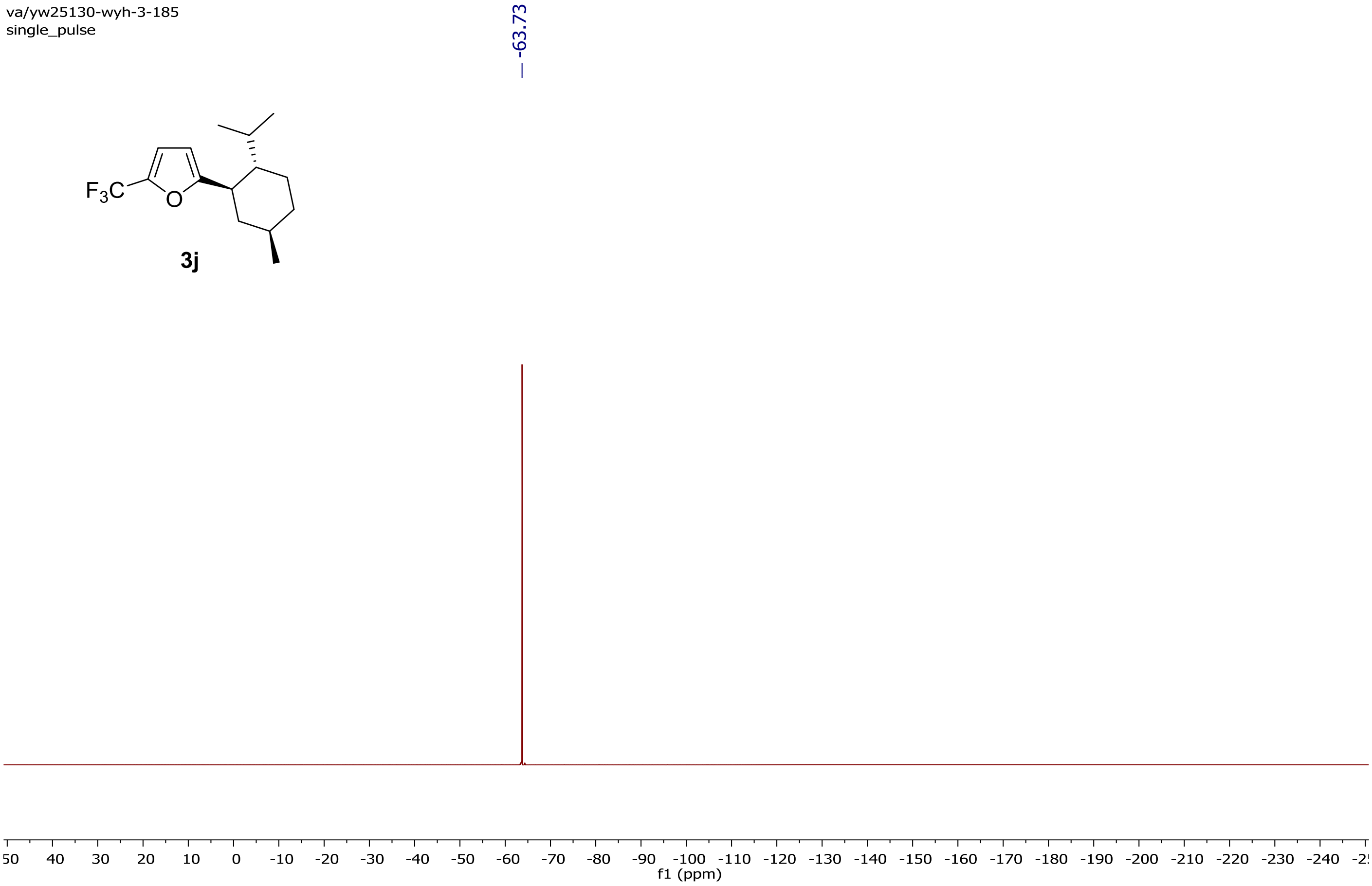
va/yw25130-wyh-3-185
single_pulse
-63.73
F3C
O
3j
f1 (ppm)

va/yw25799-wyh-4-18-n
single_pulse

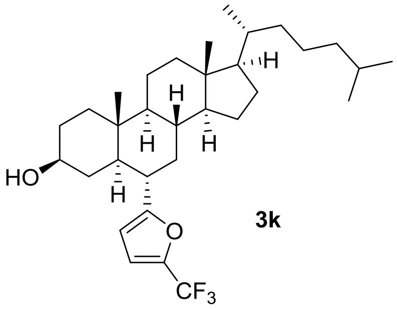

3k

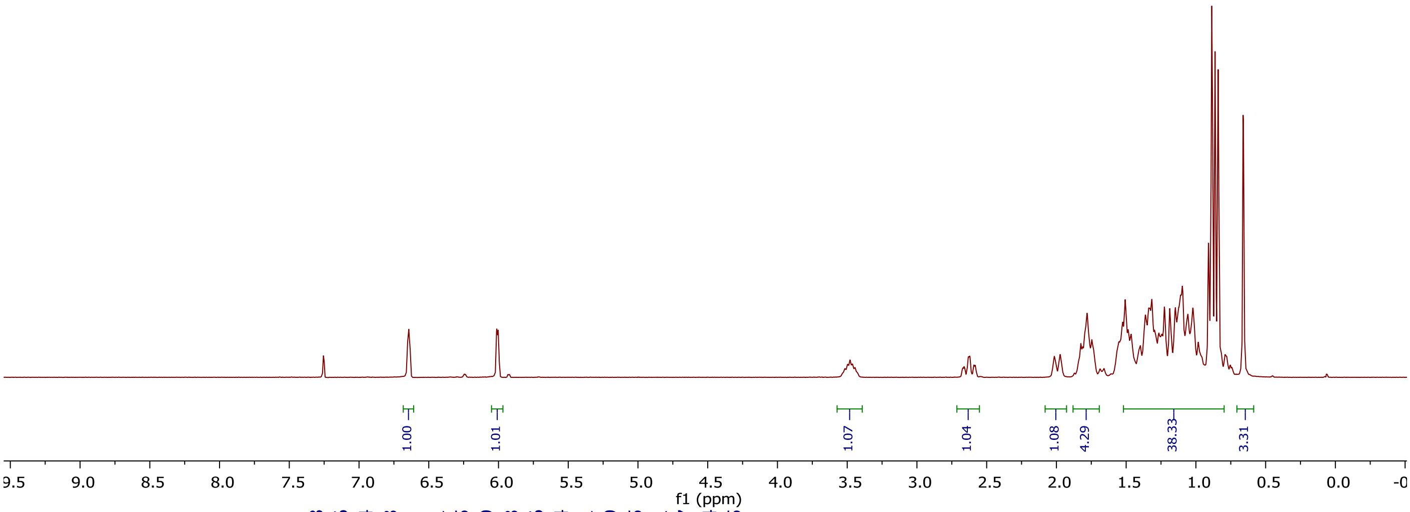

va/yw25799-wyh-4-18-n
single pulse decoupled gated NOE

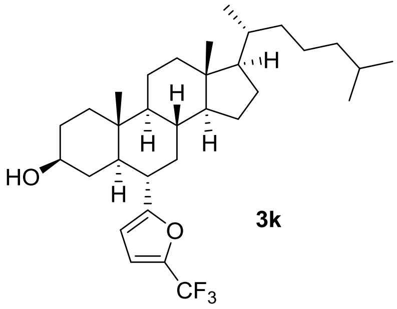

3k

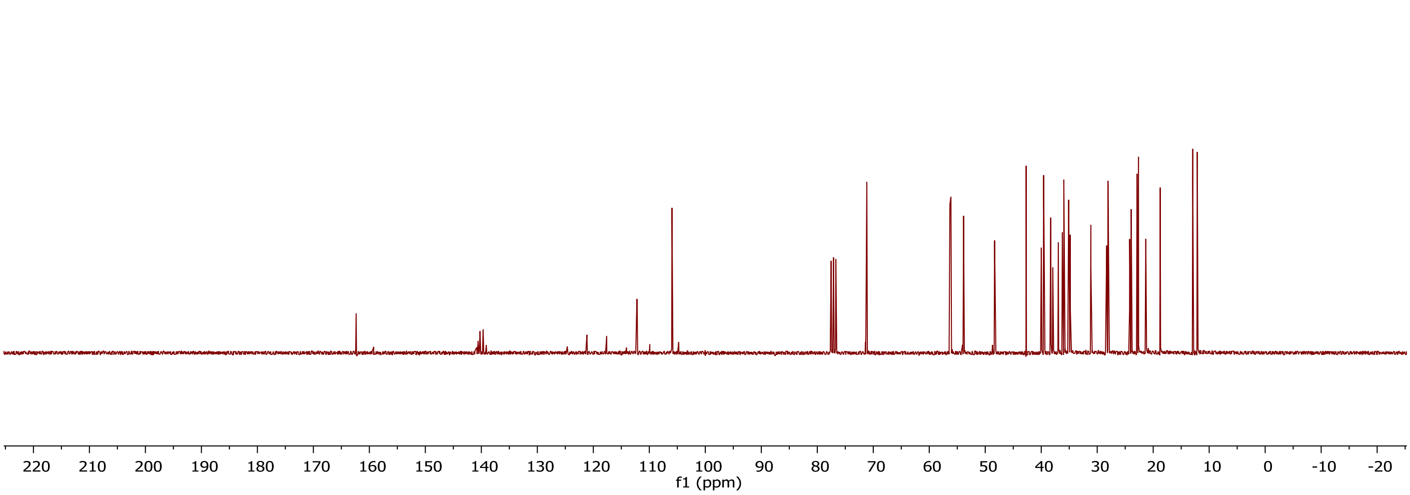

va/yw25799-wyh-4-18-n
single_pulse

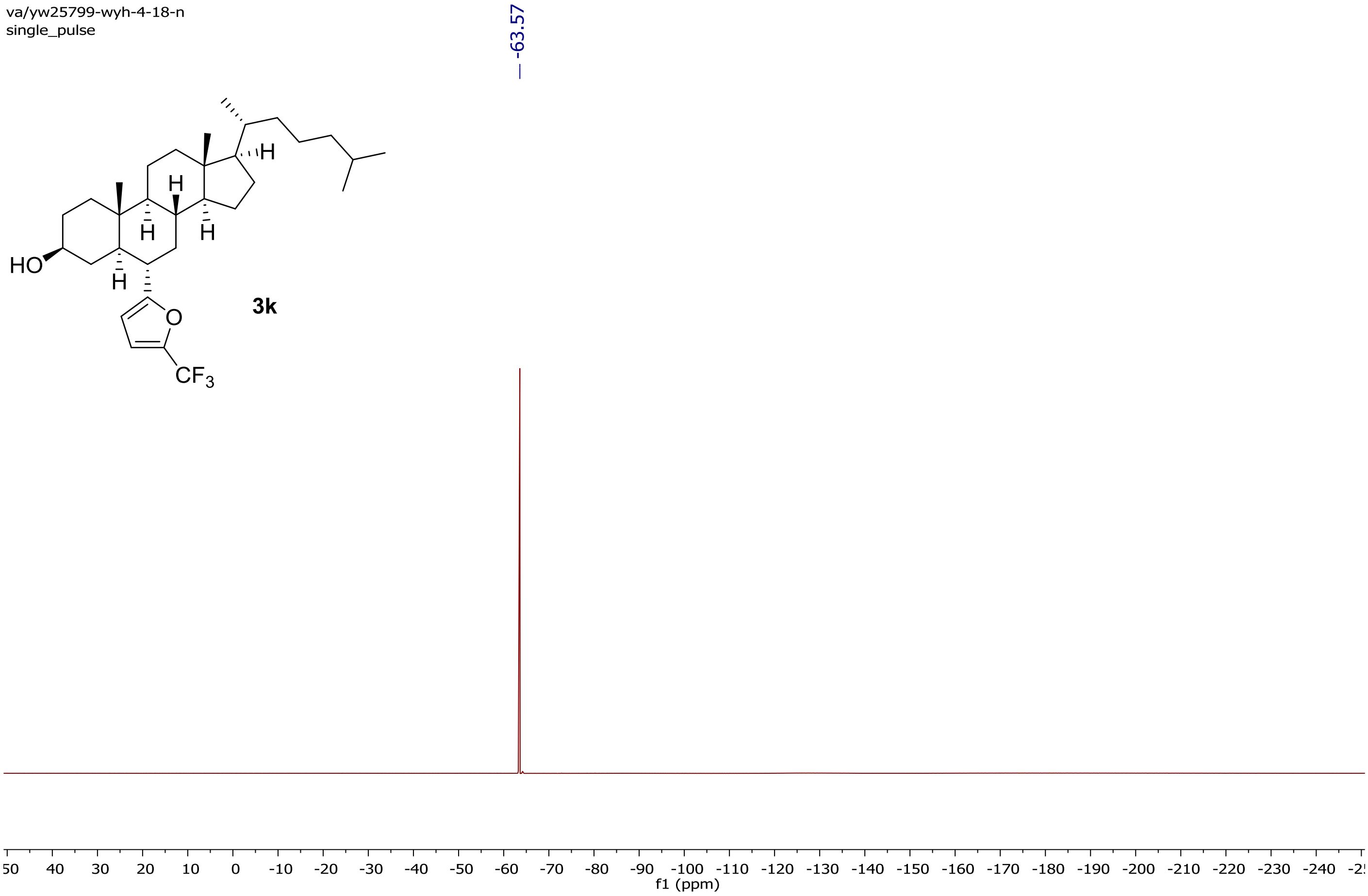

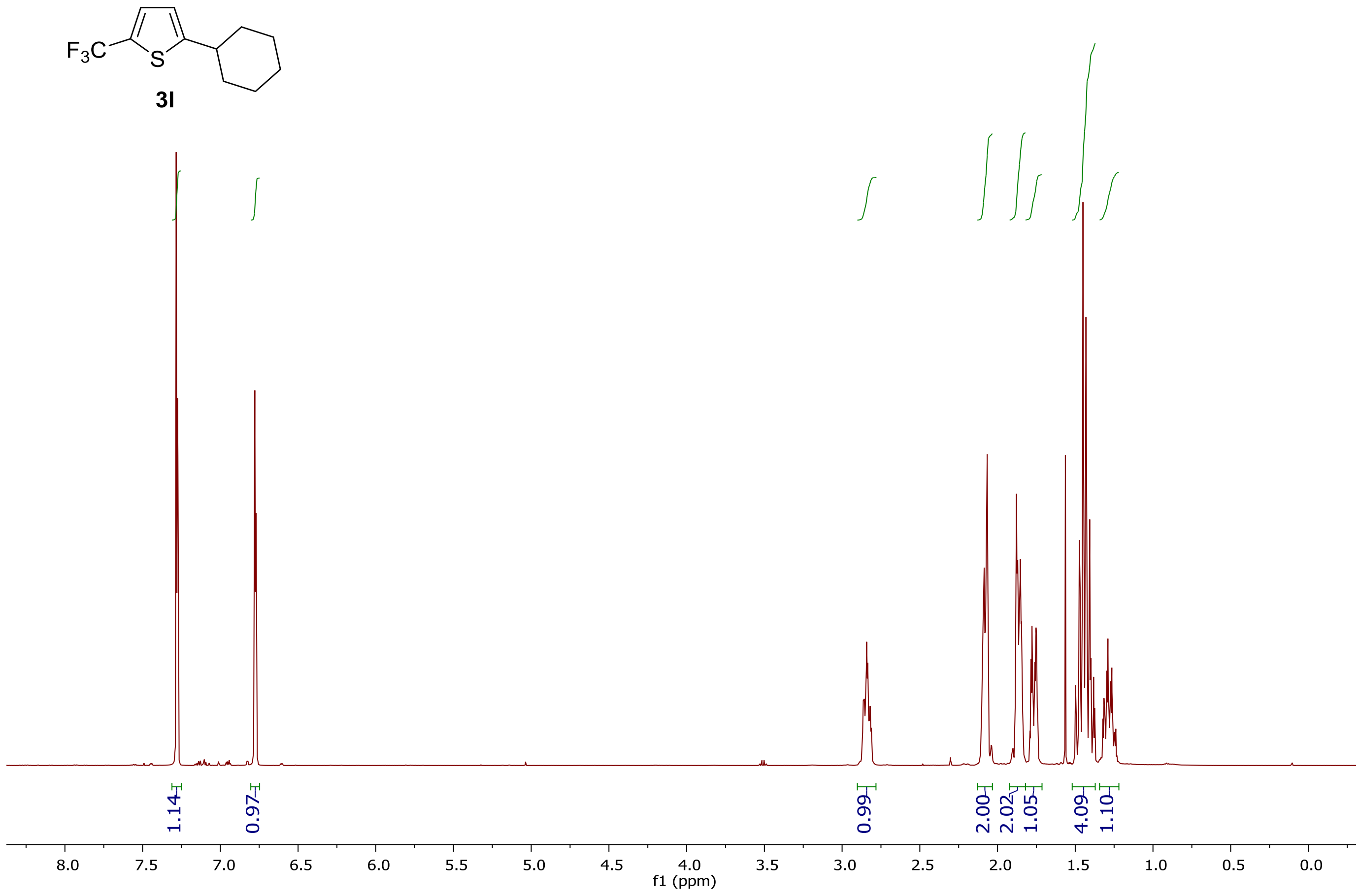

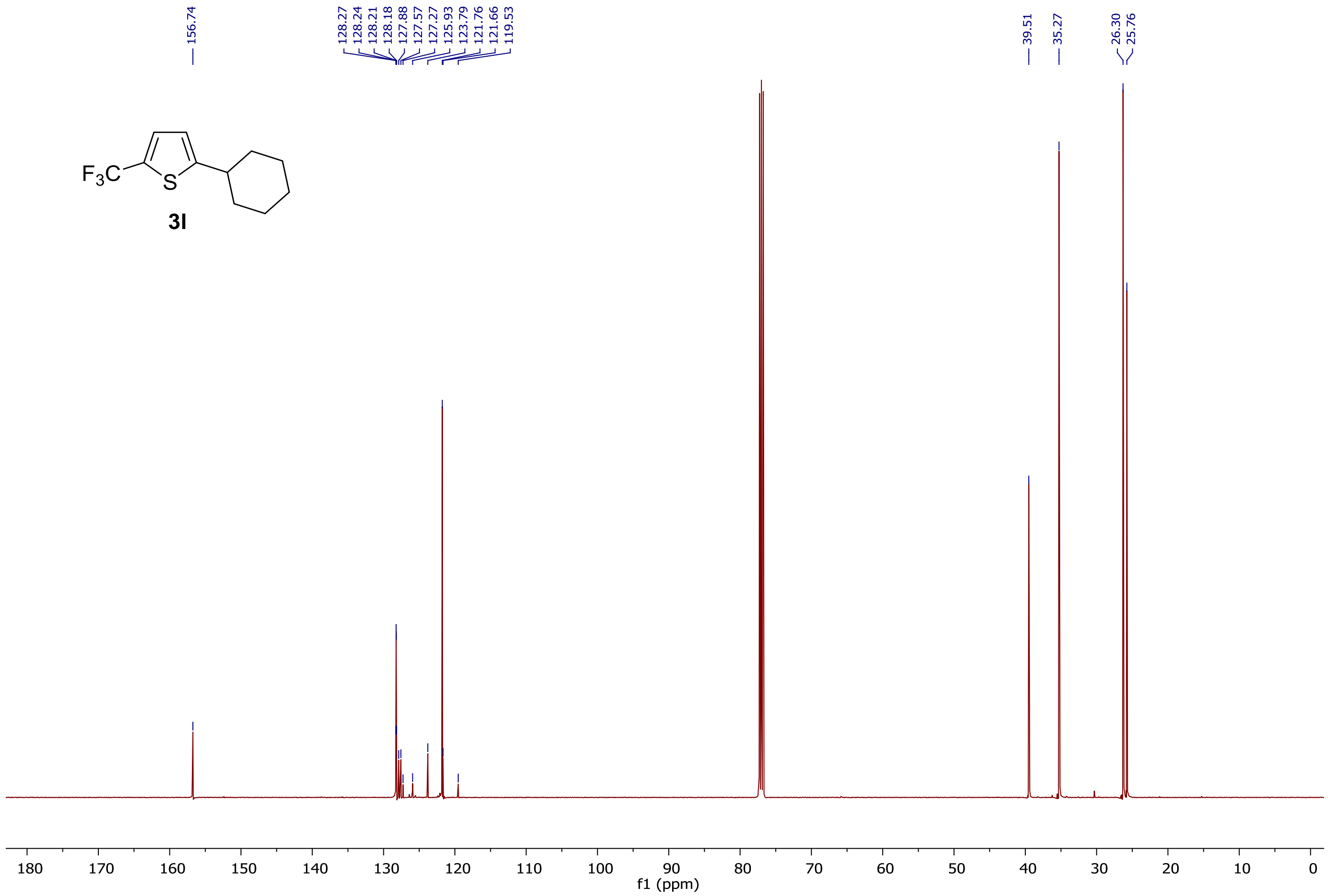
156.74
128.27
128.24
128.21
128.18
127.88
127.57
127.27
125.93
123.79
121.76
121.66
119.53
39.51
35.27
26.30
25.76
F3C
S
3l
180 170 160 150 140 130 120 110 100 90 80 70 60 50 40 30 20 10 0
f1 (ppm)

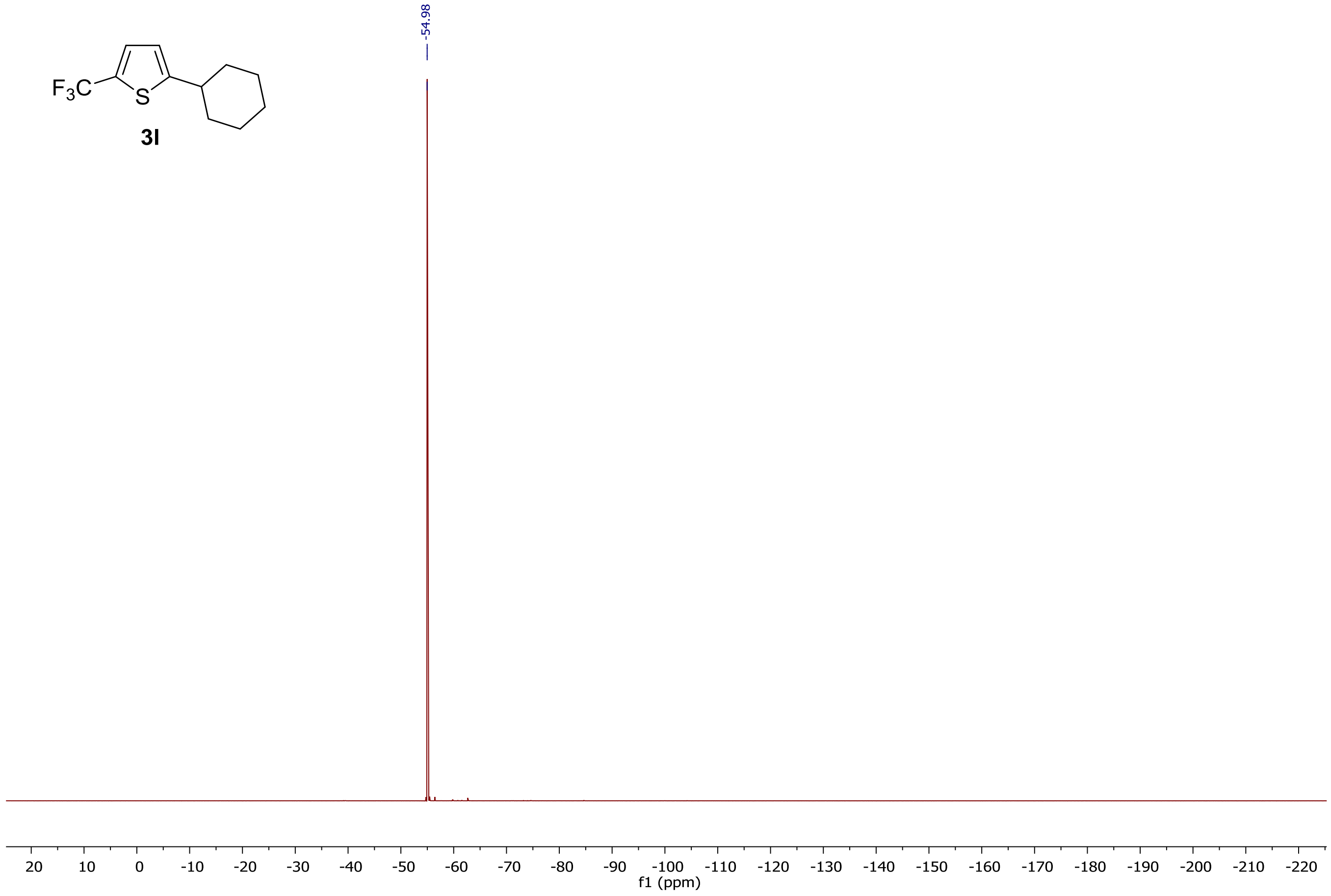
-54.98
F3C
S
3l
20 10 0 -10 -20 -30 -40 -50 -60 -70 -80 -90 -100 -110 -120 -130 -140 -150 -160 -170 -180 -190 -200 -210 -220
f1 (ppm)

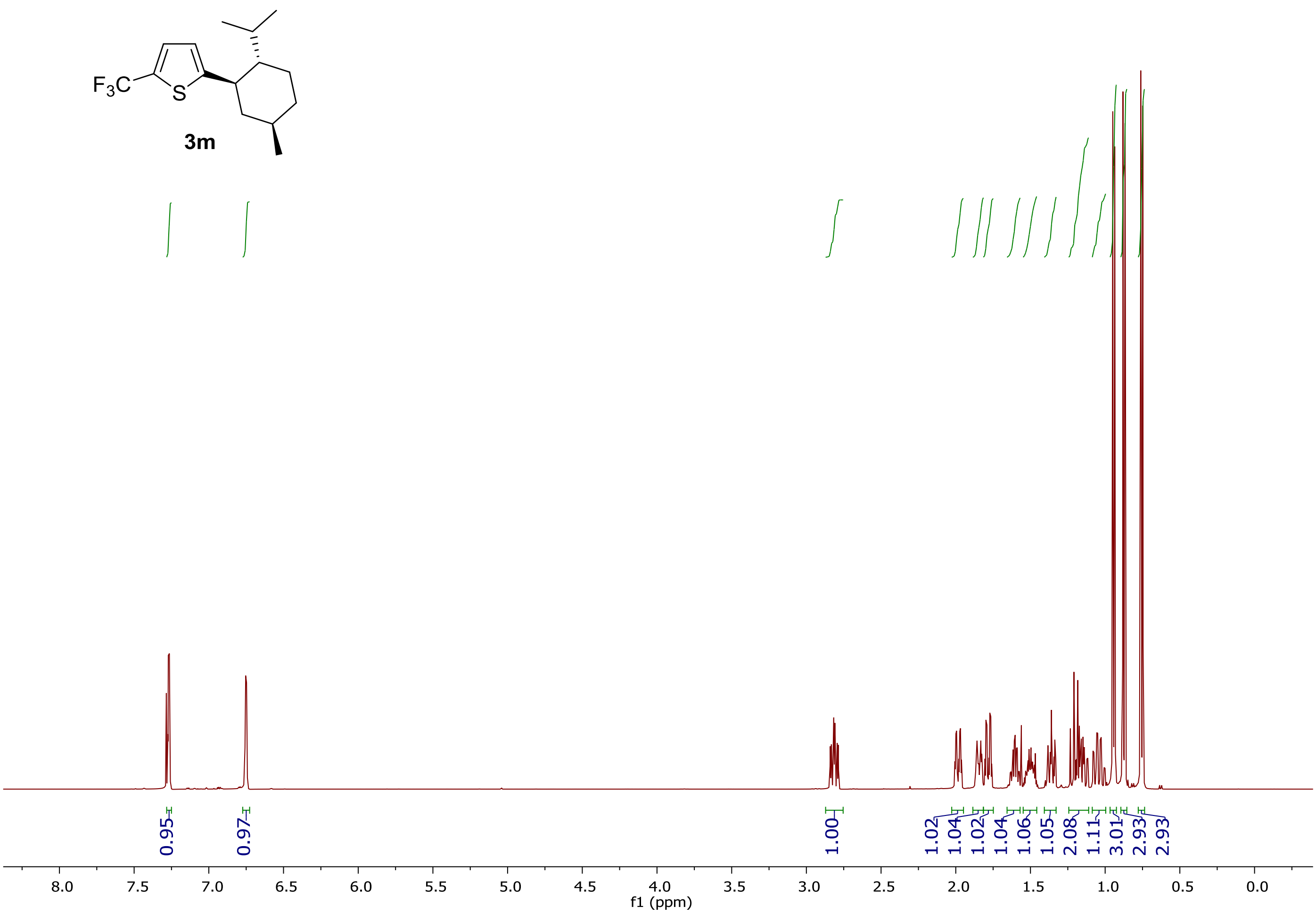
3m
0.95
0.97
1.00
1.02
1.04
1.02
1.04
1.06
1.05
2.08
1.11
3.01
2.93
2.93
8.0
7.5
7.0
6.5
6.0
5.5
5.0
4.5
4.0
3.5
3.0
2.5
2.0
1.5
1.0
0.5
0.0
f1 (ppm)

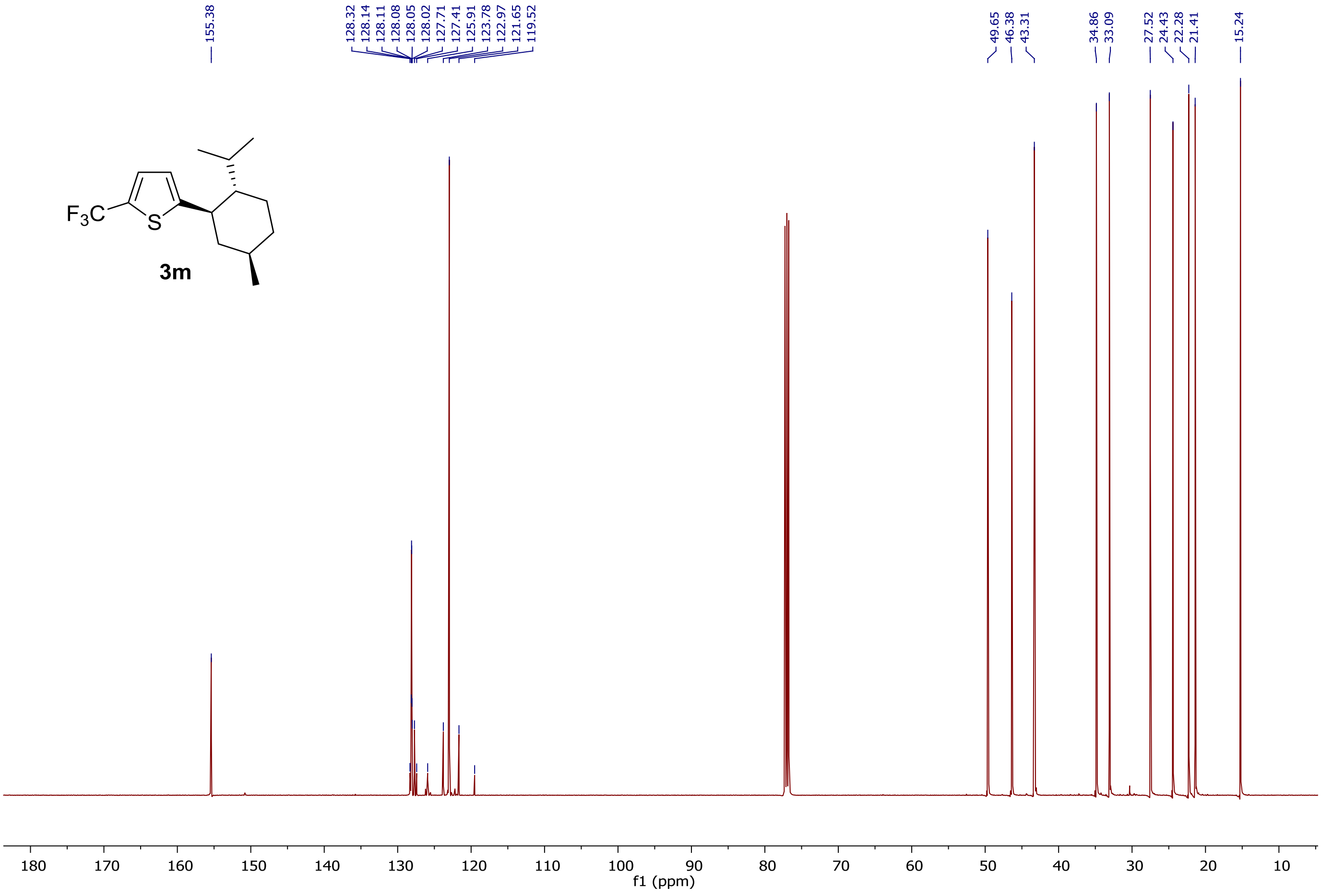
155.38
128.32
128.14
128.11
128.08
128.05
128.02
127.71
127.41
125.91
123.78
122.97
121.65
119.52
49.65
46.38
43.31
34.86
33.09
27.52
24.43
22.28
21.41
15.24
3m
180
170
160
150
140
130
120
110
100
90
80
70
60
50
40
30
20
10
f1 (ppm)

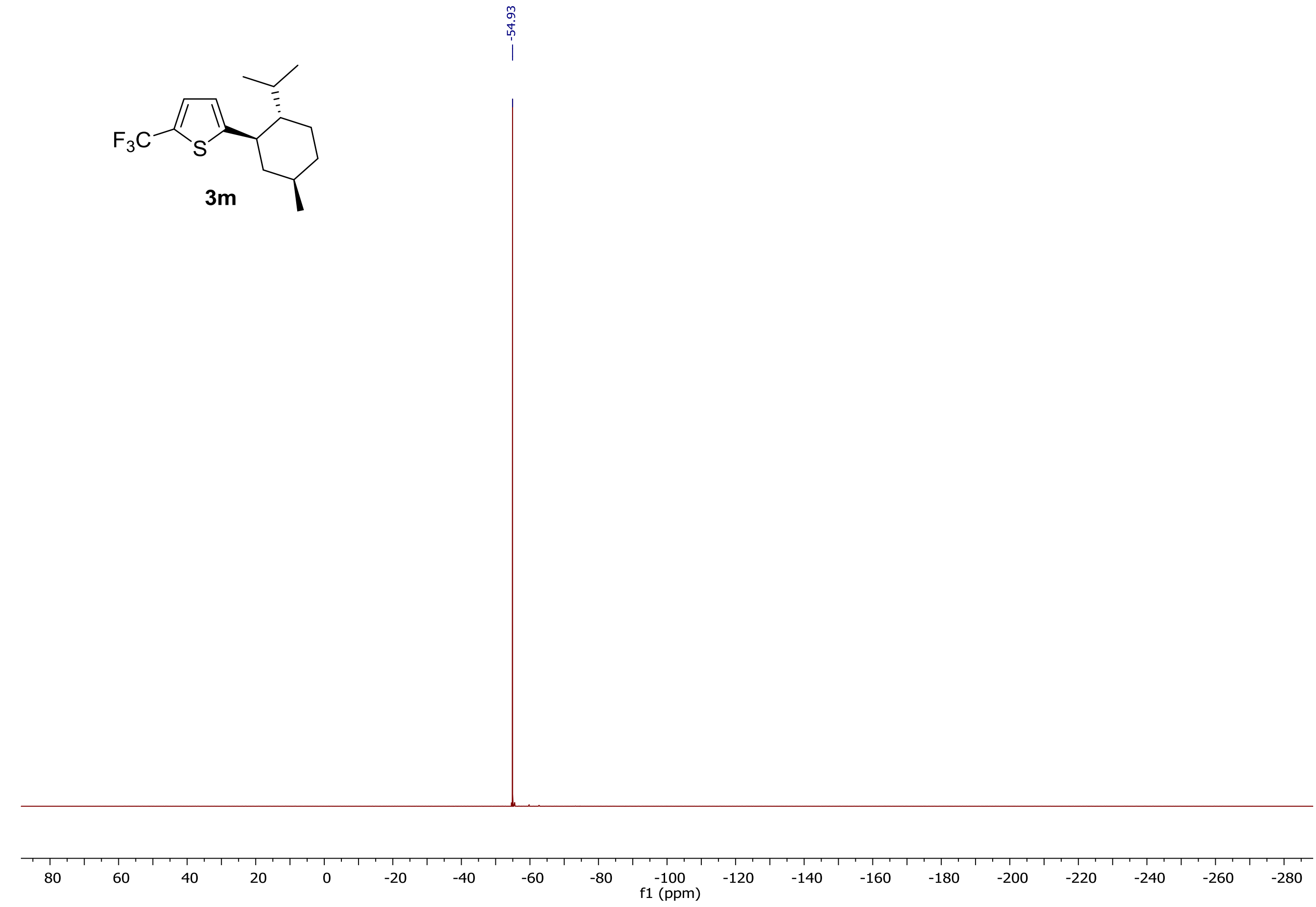
-54.93
F3C
S
3m
80 60 40 20 0 -20 -40 -60 -80 -100 -120 -140 -160 -180 -200 -220 -240 -260 -280
f1 (ppm)

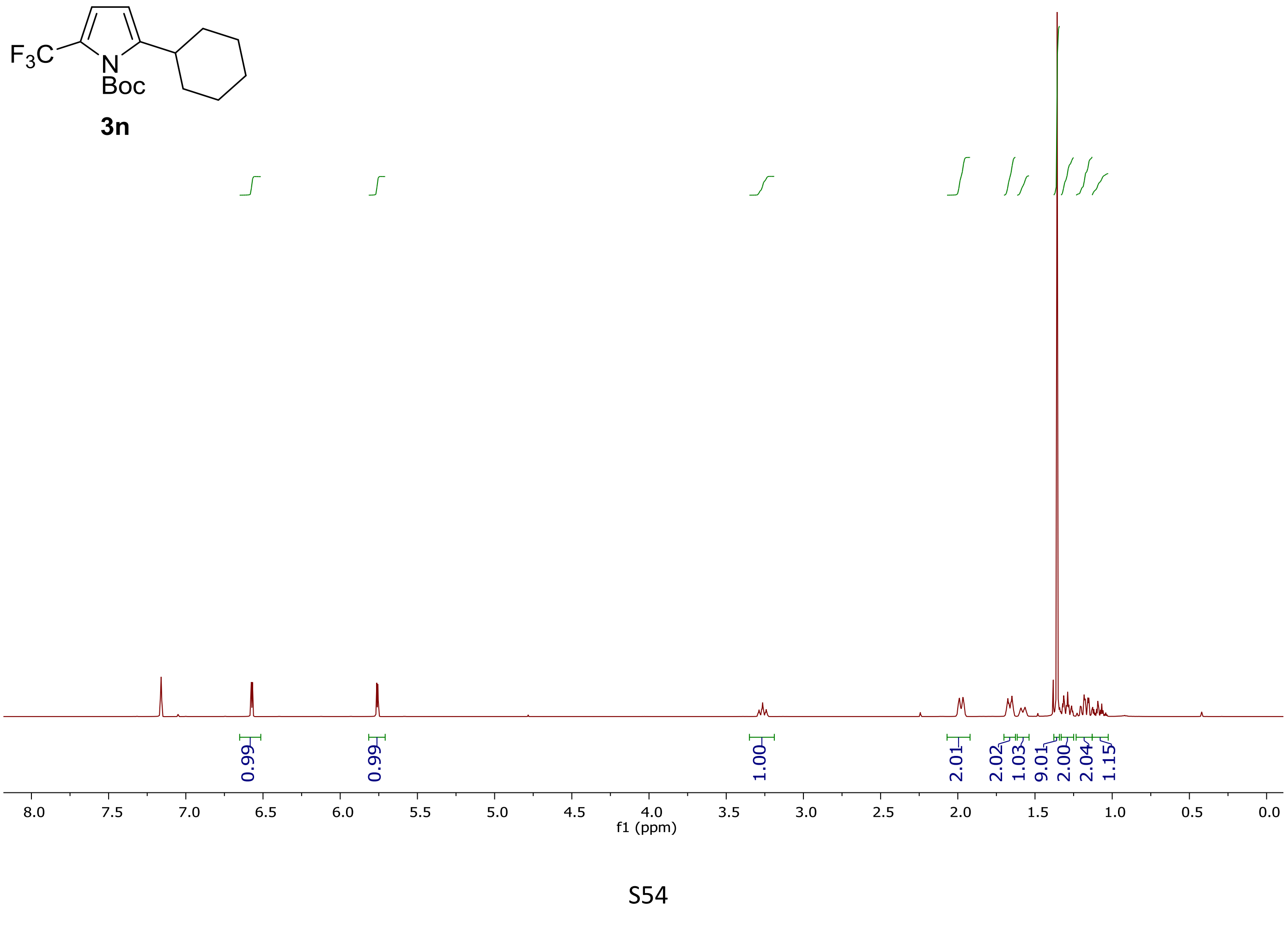
F3C
N
Boc
3n
0.99
0.99
1.00
2.01
2.02
1.03
9.01
2.00
2.04
1.15
8.0 7.5 7.0 6.5 6.0 5.5 5.0 4.5 4.0 3.5 3.0 2.5 2.0 1.5 1.0 0.5 0.0
f1 (ppm)

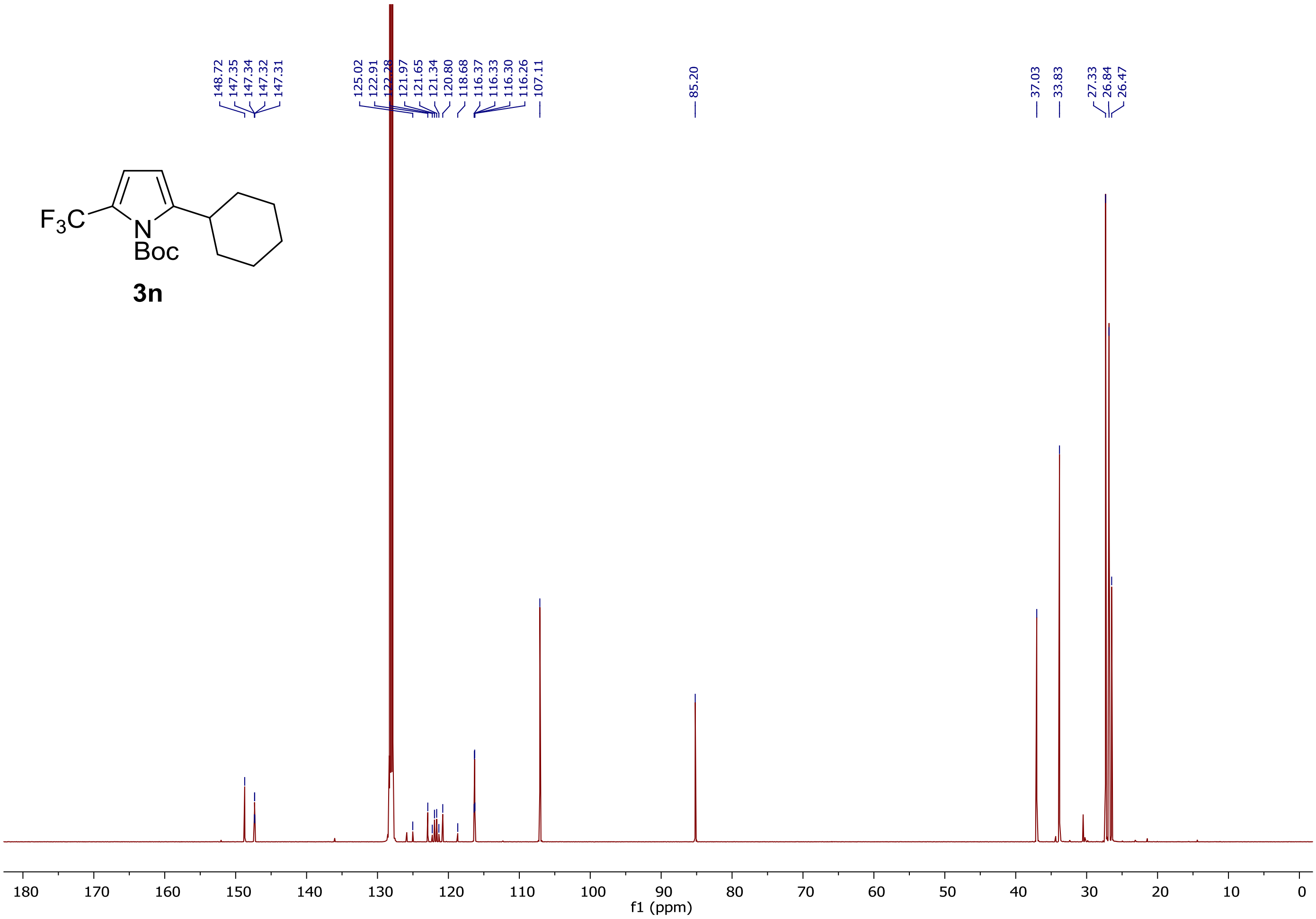
148.72
147.35
147.34
147.32
147.31
125.02
122.91
121.97
121.65
121.34
120.80
118.68
116.37
116.33
116.30
116.26
107.11
85.20
37.03
33.83
27.33
26.84
26.47
F3C
N
Boc
3n
f1 (ppm)

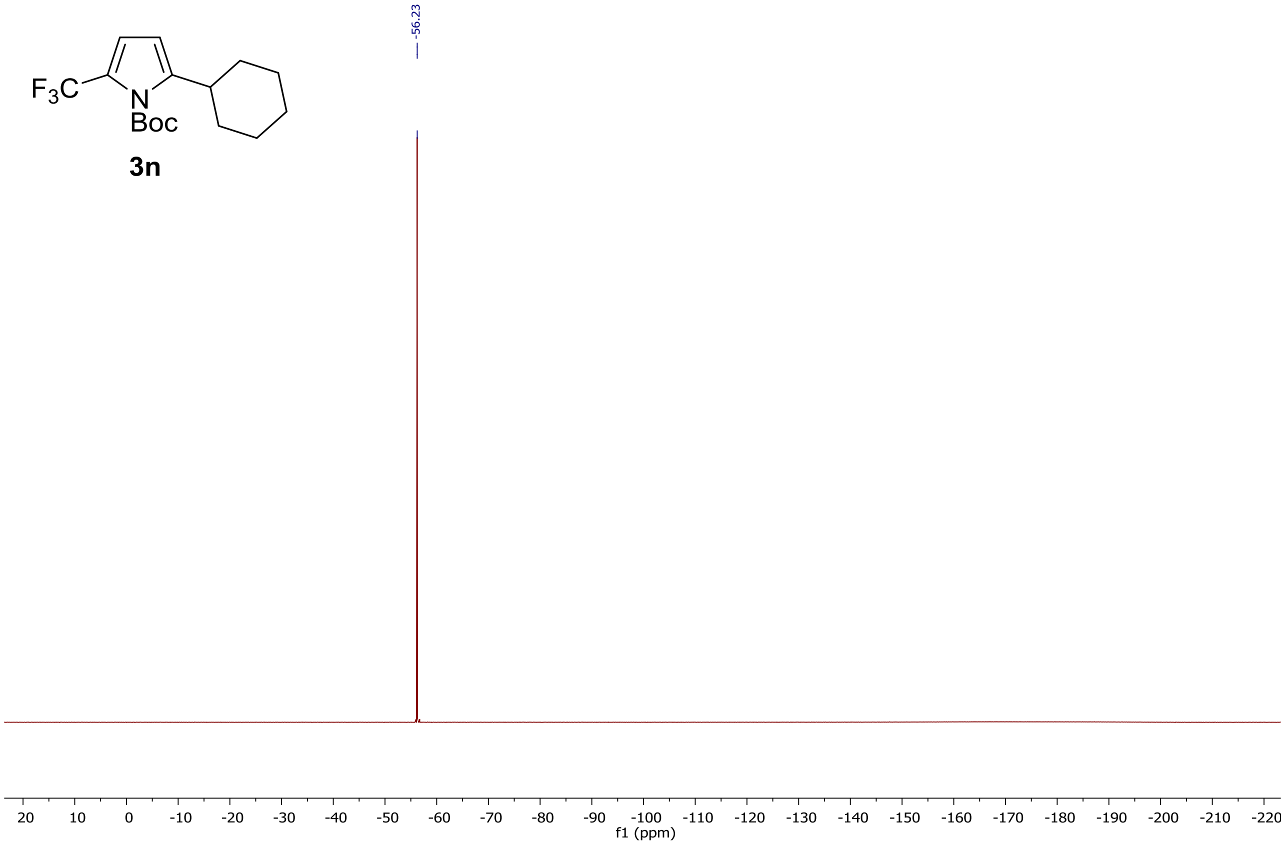
-56.23
F3C
N
Boc
3n
f1 (ppm)

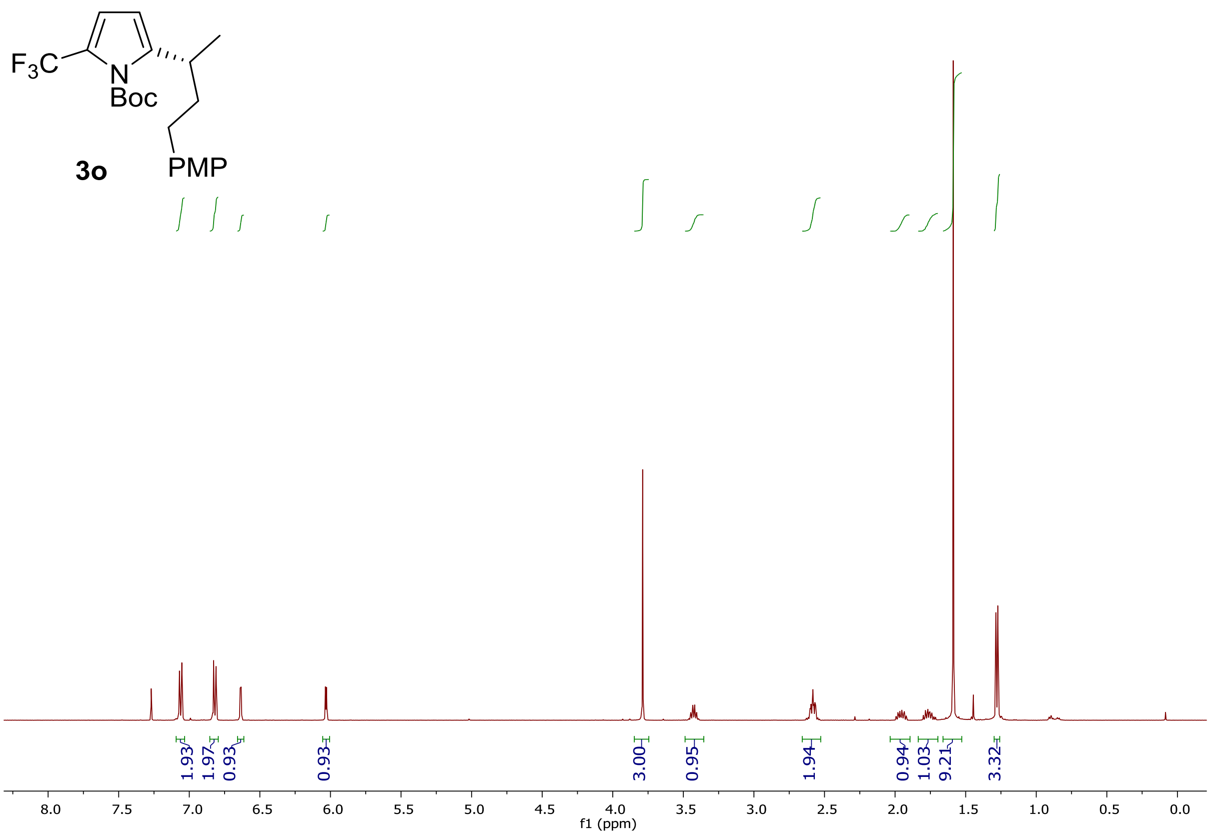

F3C
N
Boc
PMP
3o
1.93
1.97
0.93
0.93
3.00
0.95
1.94
0.94
1.03
9.21
3.32
f1 (ppm)

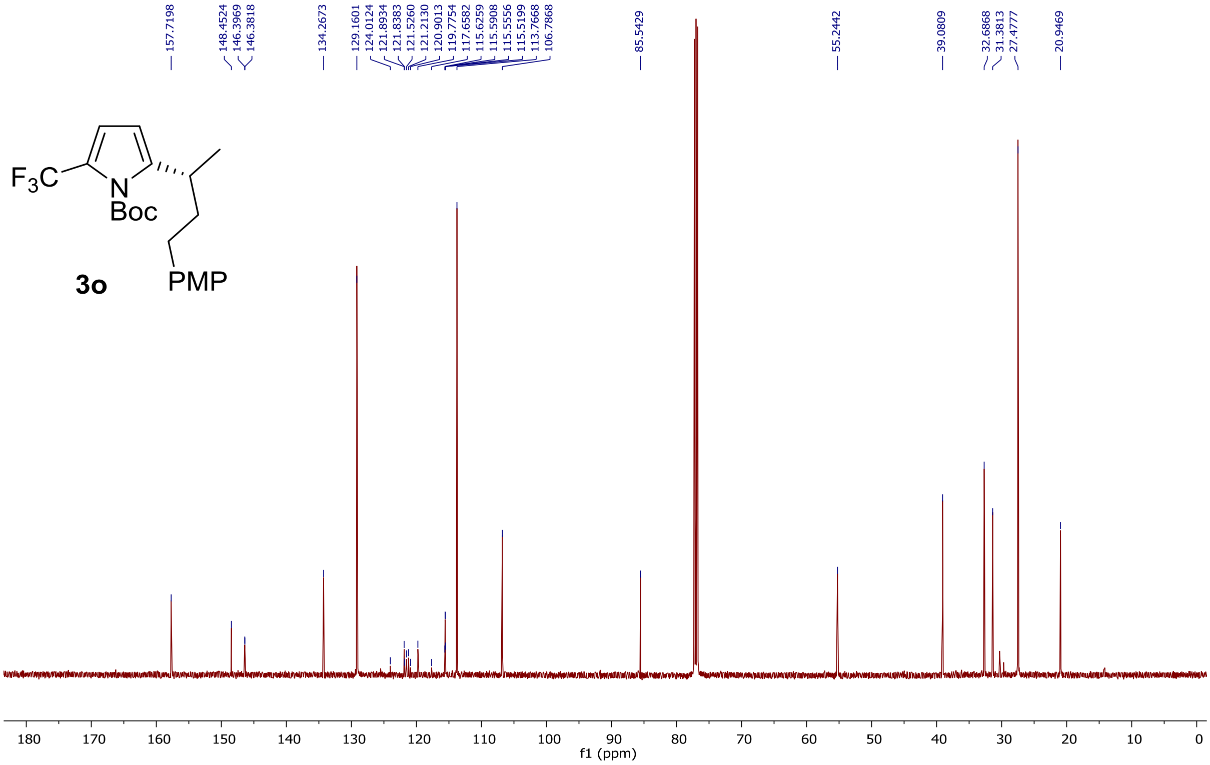

157.7198
148.4524
146.3969
146.3818
134.2673
129.1601
124.0124
121.8934
121.8383
121.5260
121.2130
120.9013
119.7754
117.6582
115.6259
115.5908
115.5556
115.5199
113.7668
106.7868
85.5429
55.2442
39.0809
32.6868
31.3813
27.4777
20.9469
F3C
N
Boc
PMP
3o
f1 (ppm)

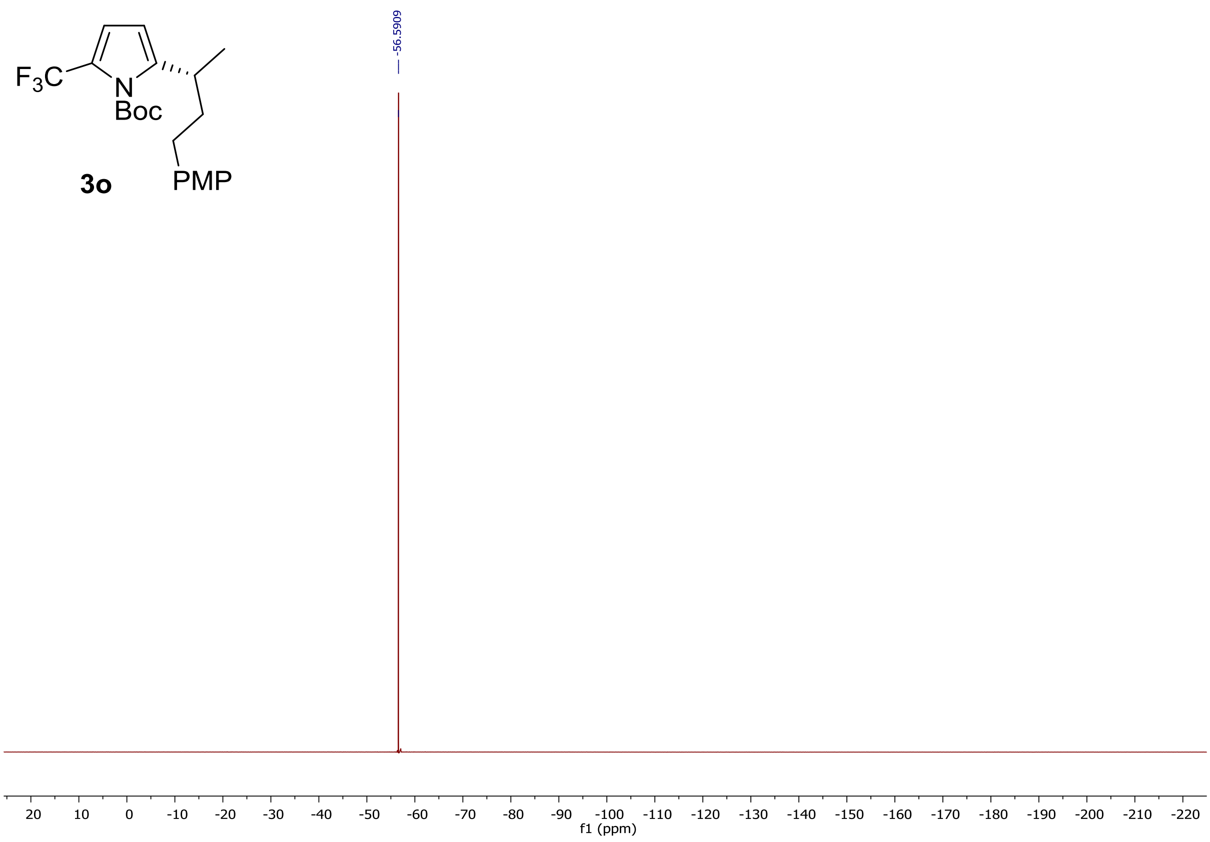

va/yw25541-wyh-4-3
single_pulse

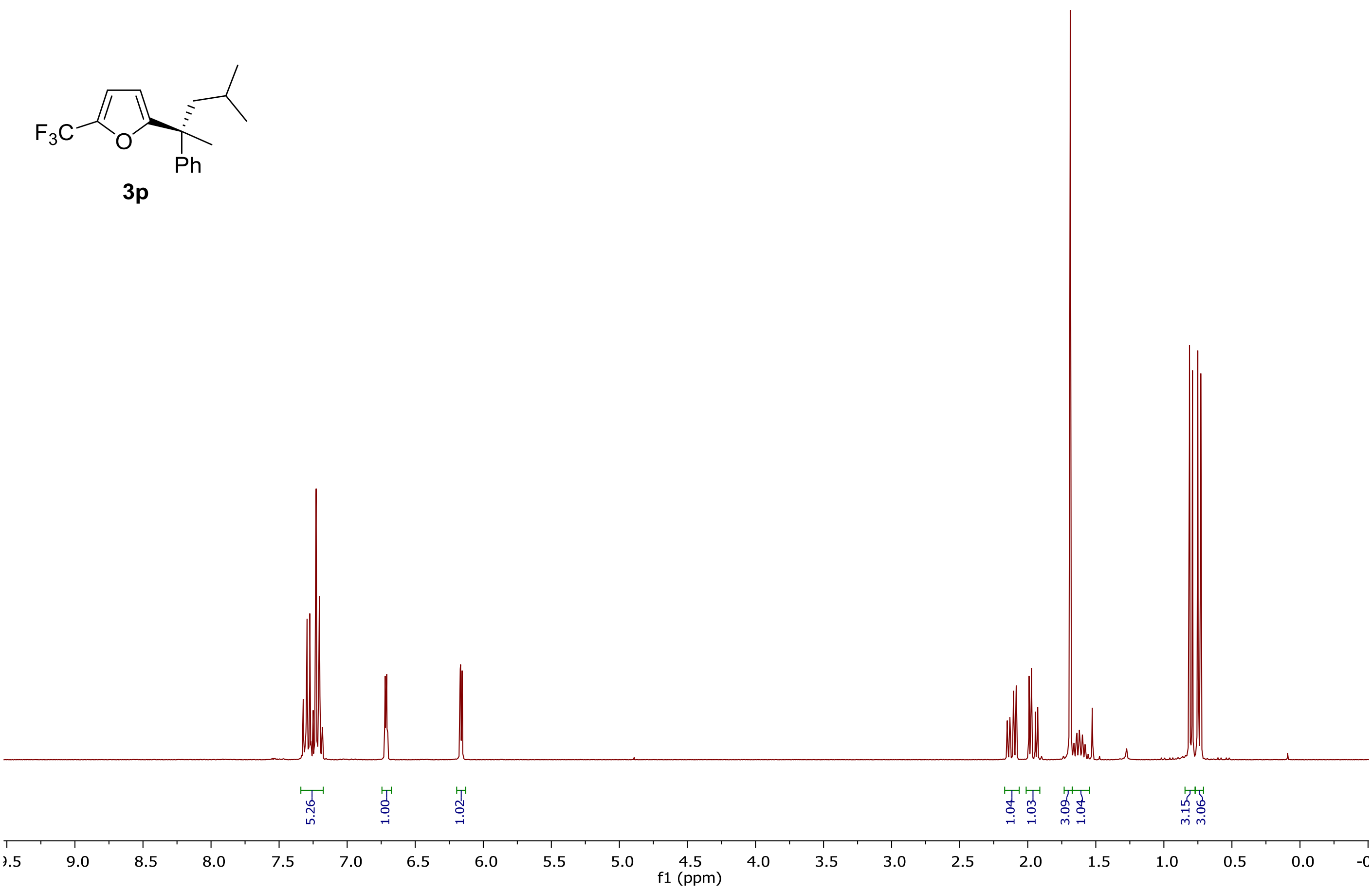

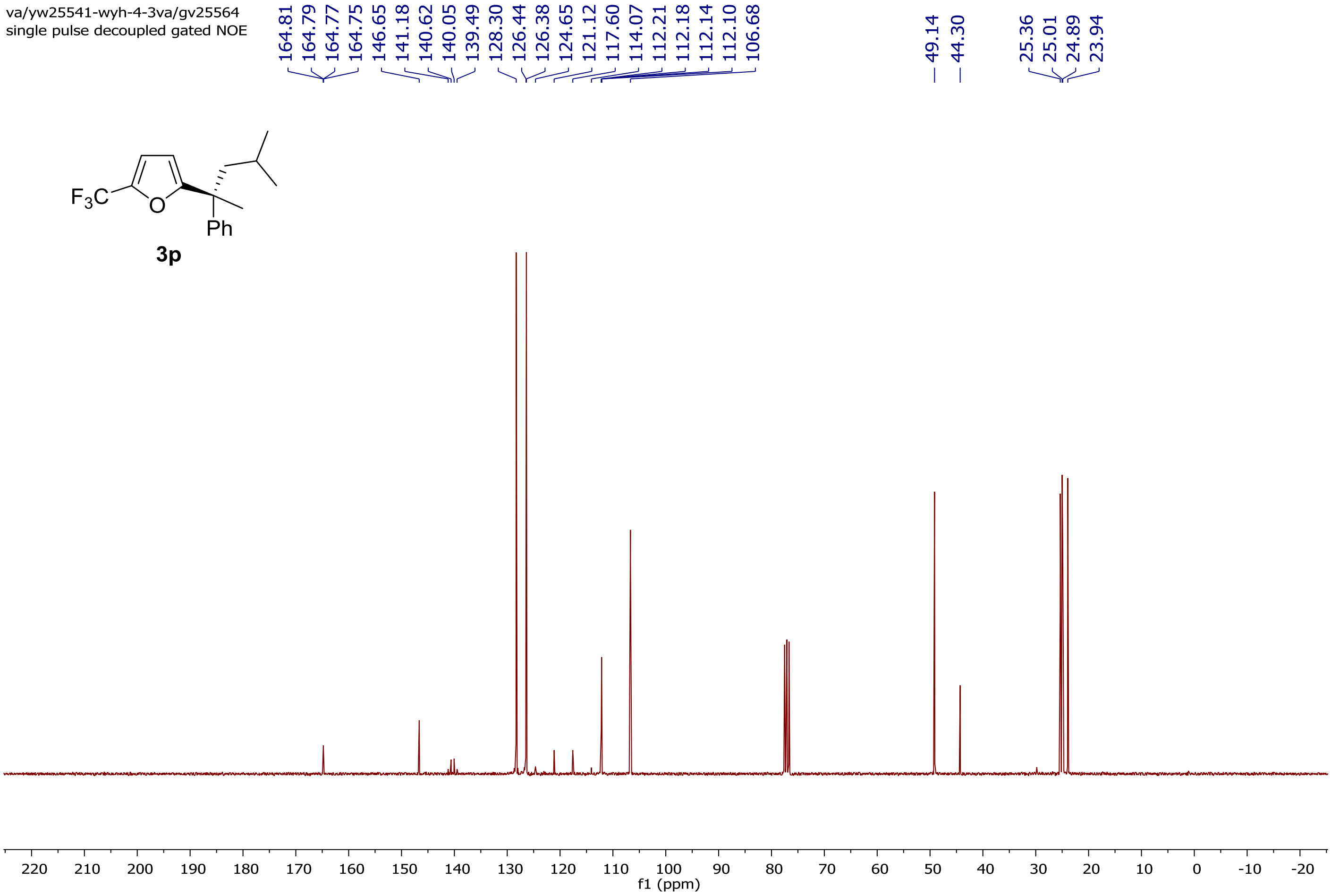
va/yw25541-wyh-4-3va/gv25564
single pulse decoupled gated NOE
164.81
164.79
164.77
164.75
146.65
141.18
140.62
140.05
139.49
128.30
126.44
126.38
124.65
121.12
117.60
114.07
112.21
112.18
112.14
112.10
106.68
49.14
44.30
25.36
25.01
24.89
23.94
F3C
O
Ph
3p
f1 (ppm)

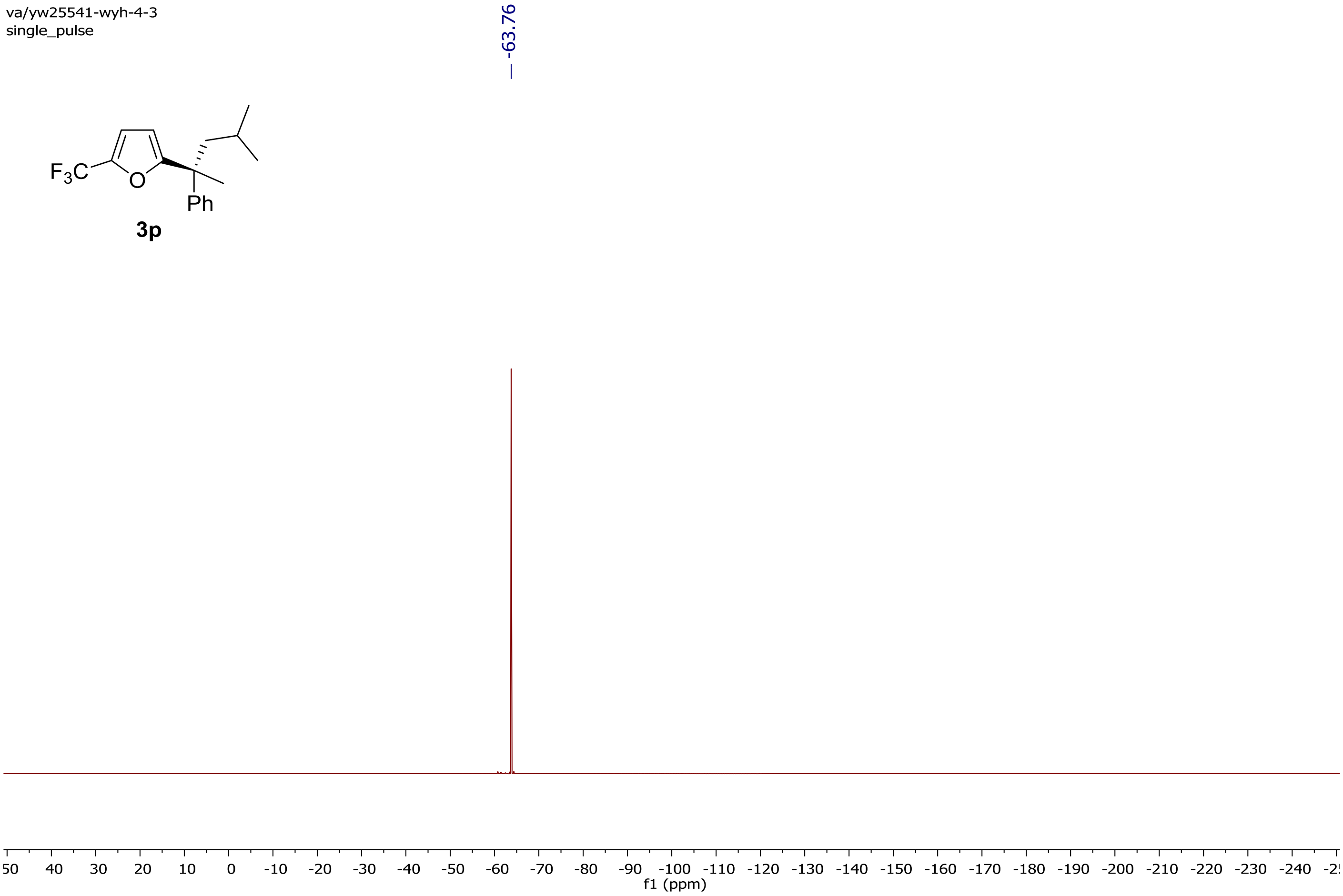
va/yw25541-wyh-4-3
single_pulse
-63.76
F3C
O
Ph
3p
f1 (ppm)

1973 wyh-4-81-n.10.fid

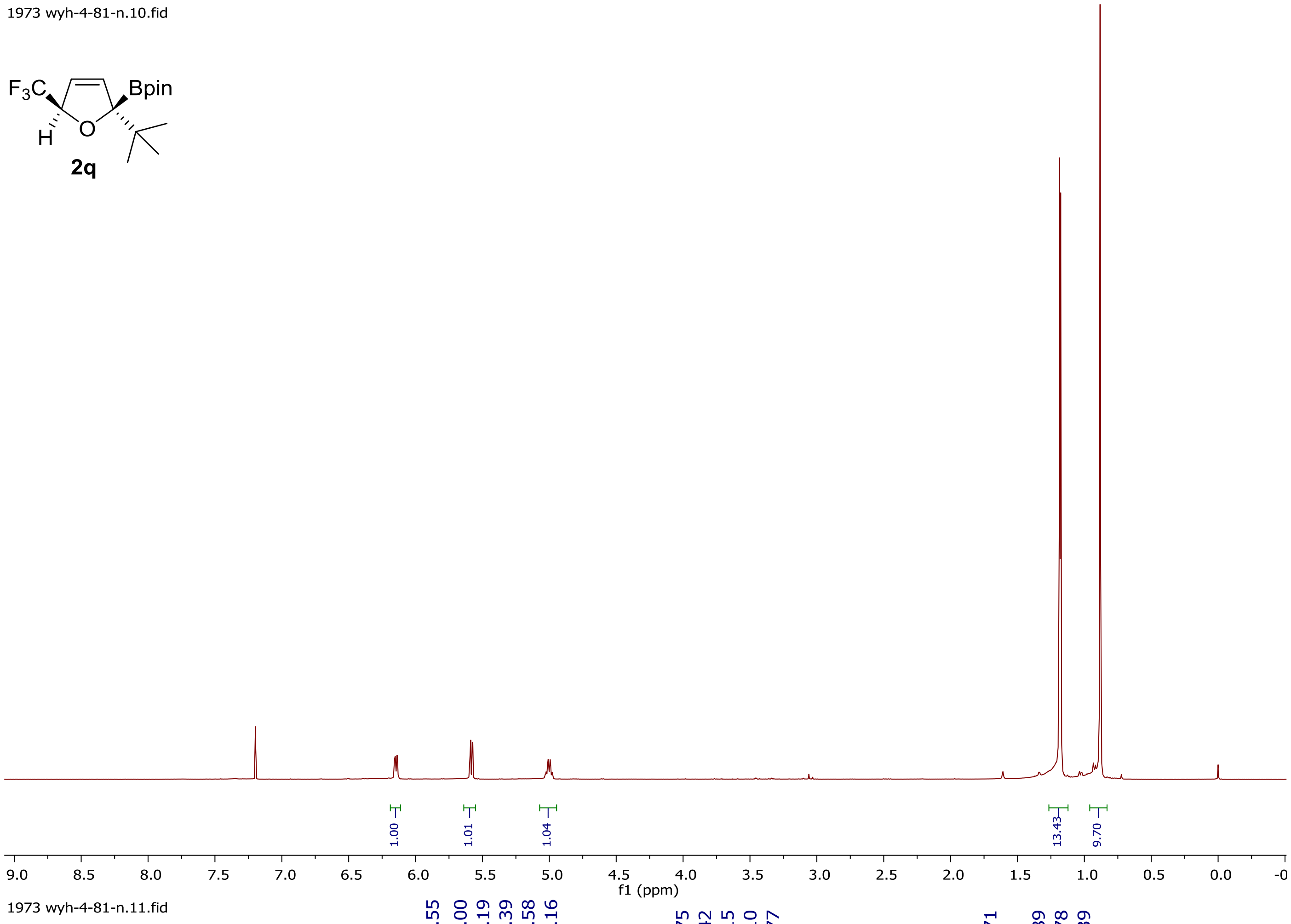

2q

1973 wyh-4-81-n.11.fid

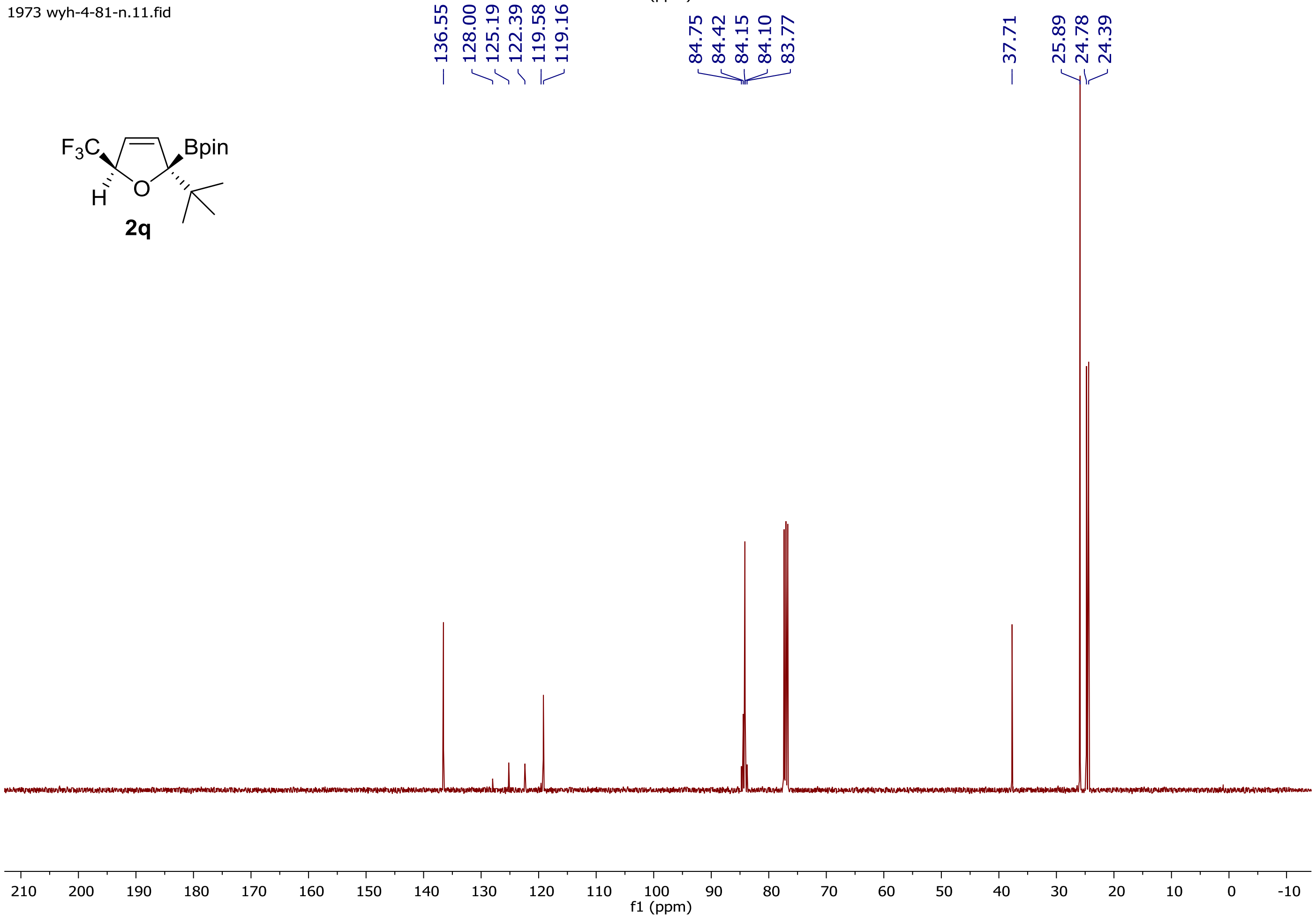

2q

1973 wyh-4-81-n.12.fid

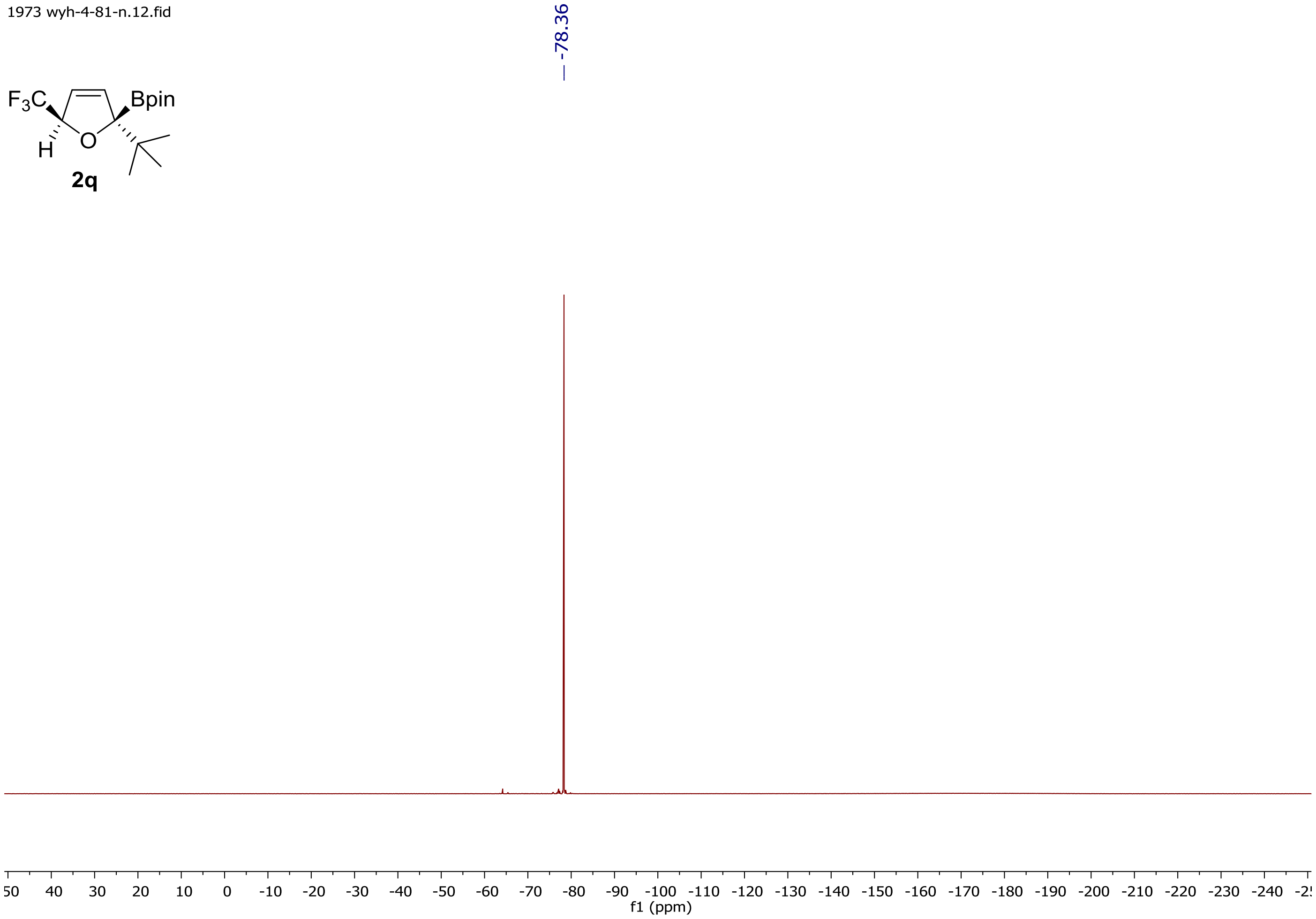

va/yw27712-wyh-4-81
single pulse decoupled gated NOE

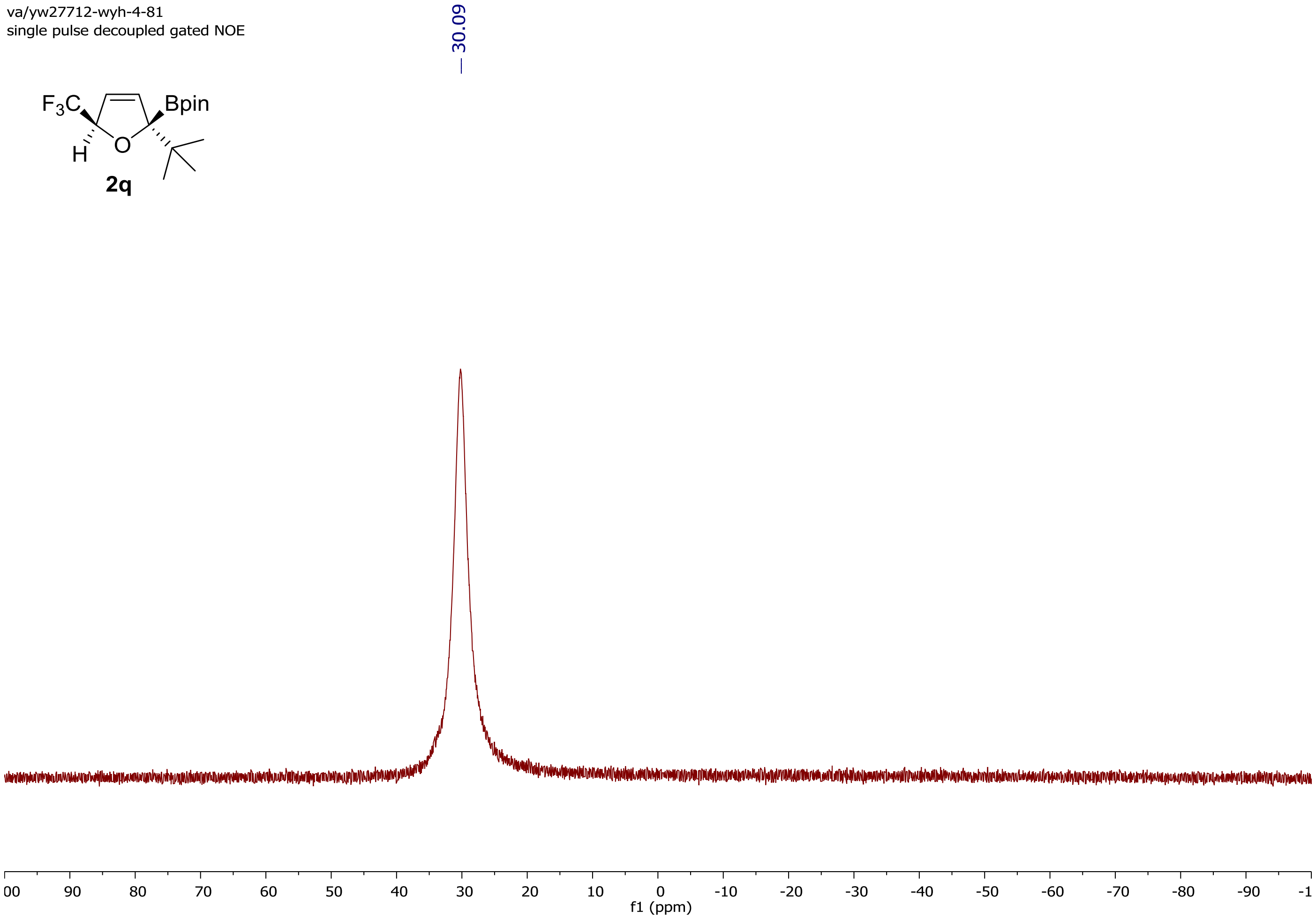

NOESY experiment of **2q**

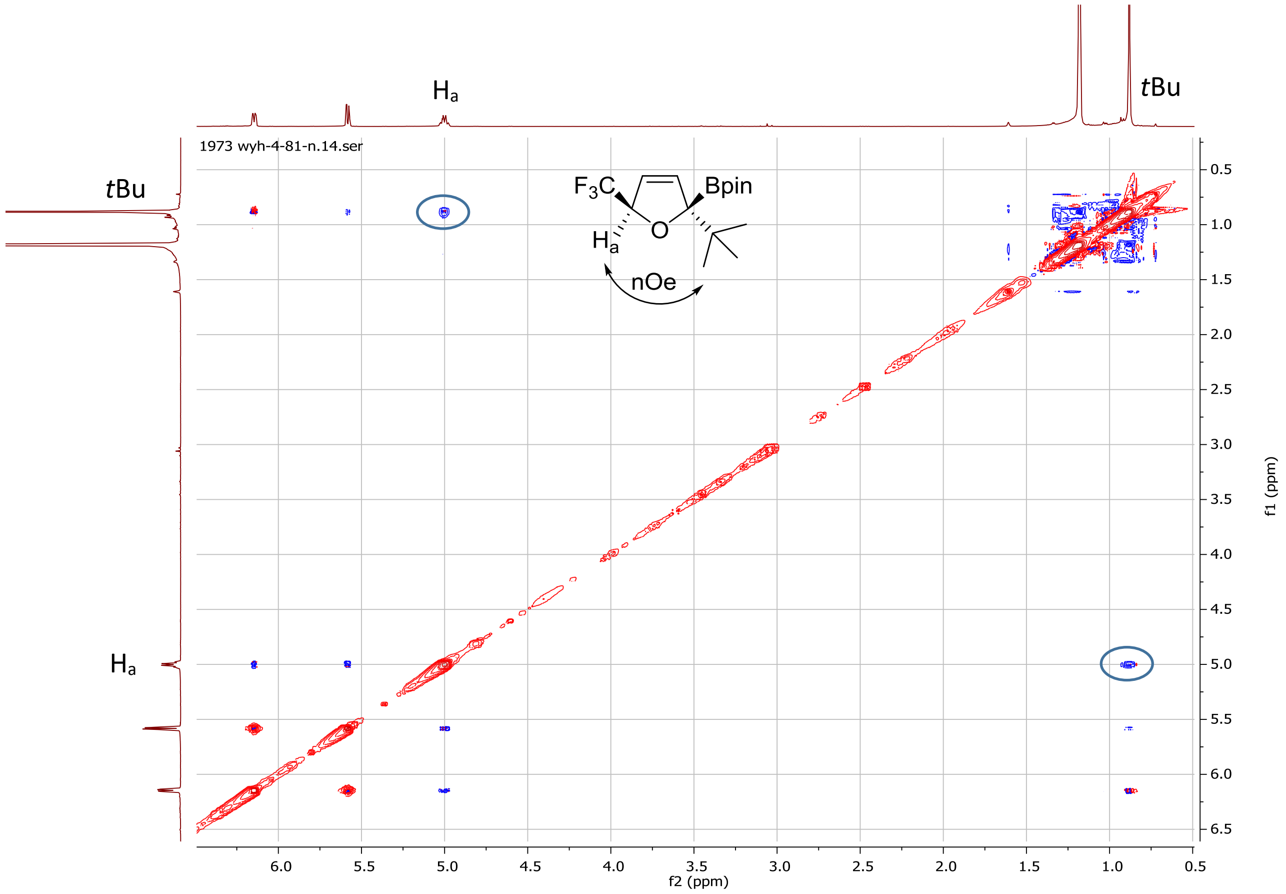

Crude **^{19}F NMR** of the reaction

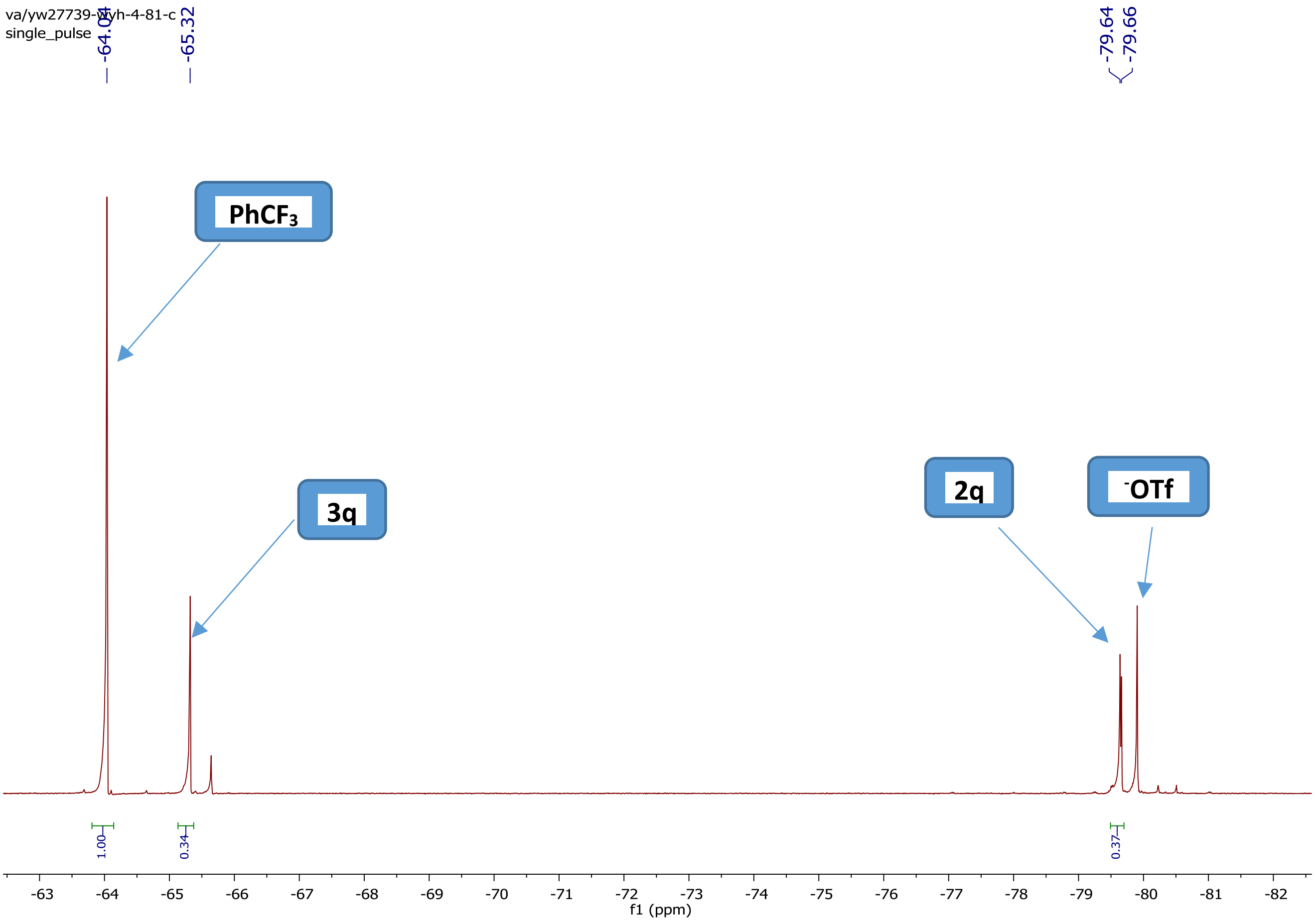

va/yw24911-wyh-3-174
single_pulse

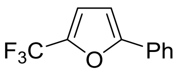

3r

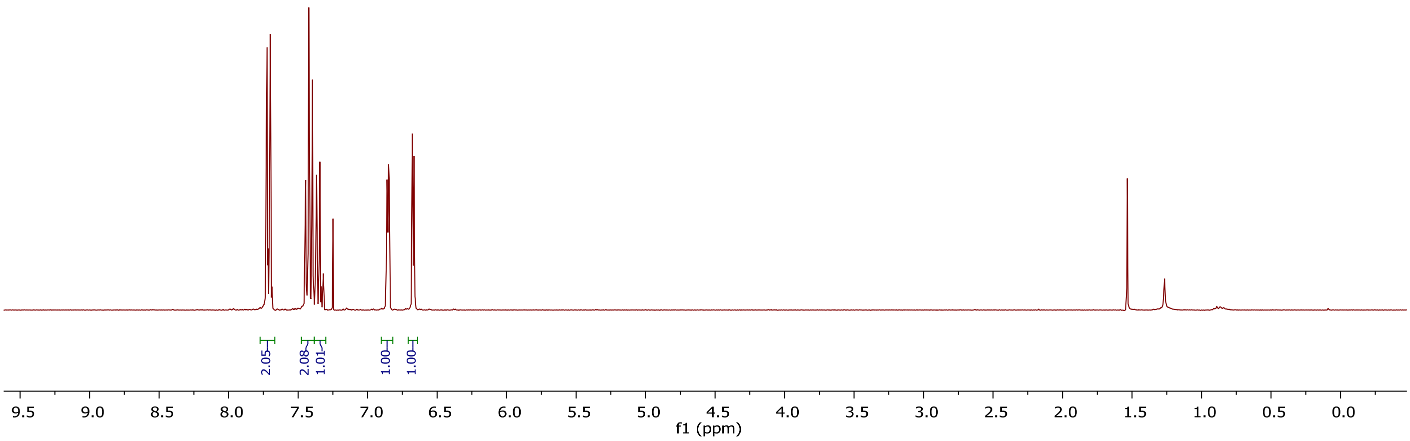

va/yw24911-wyh-3-174
single pulse decoupled gated NOE

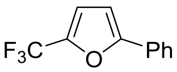

3r

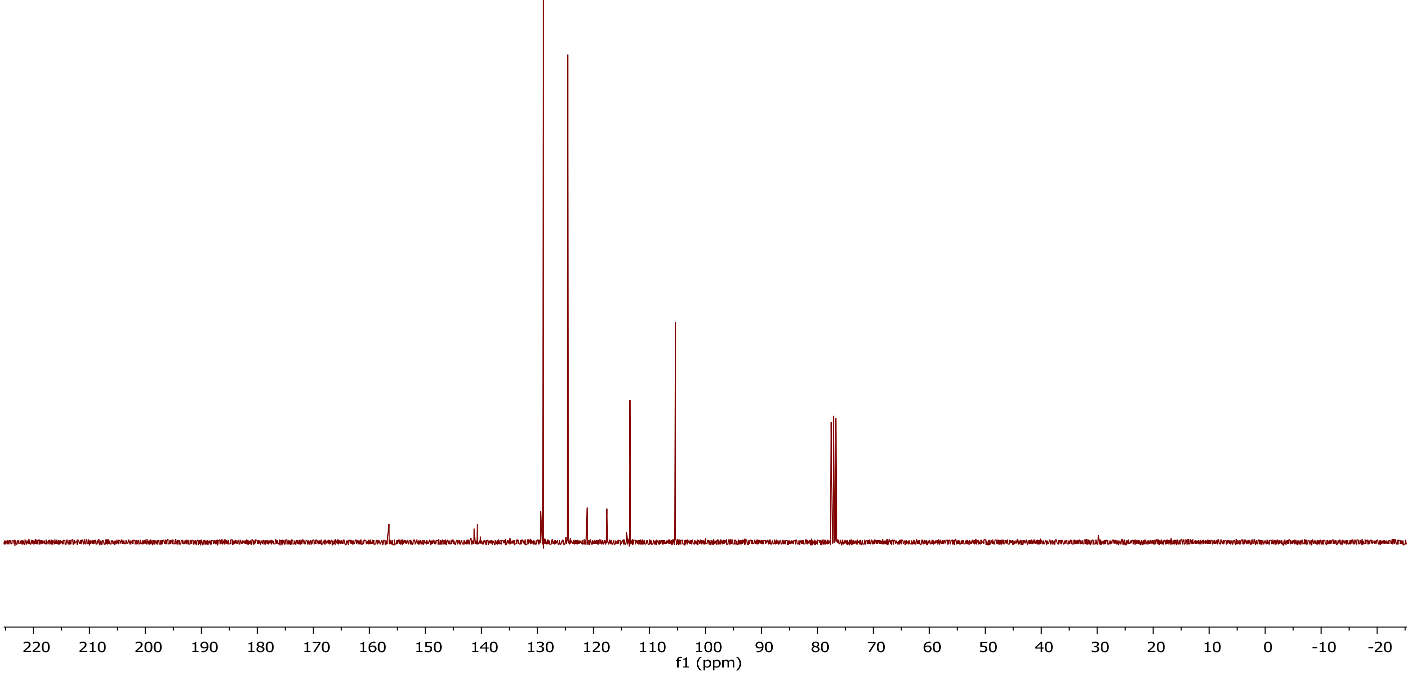

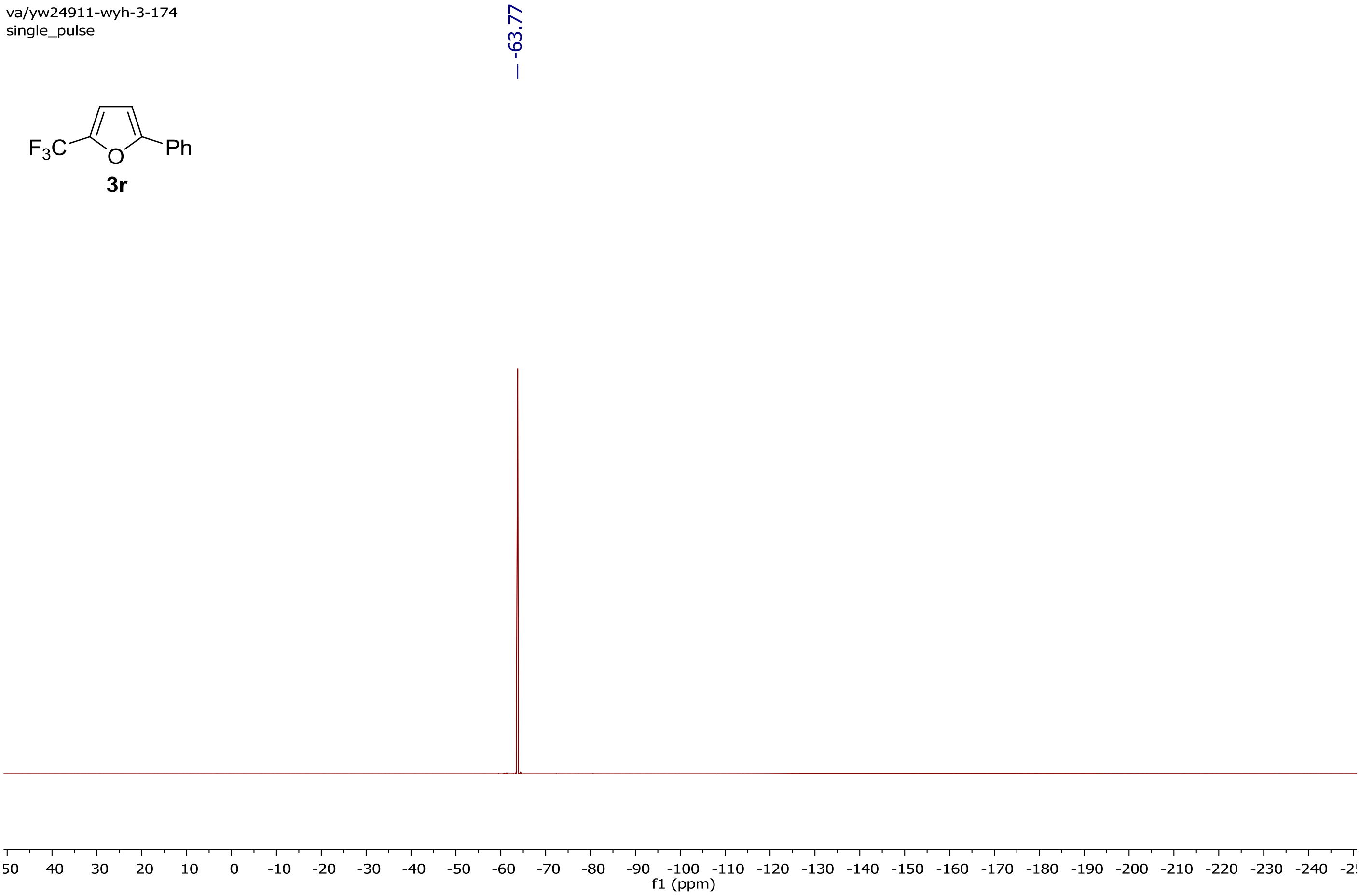
va/yw24911-wyh-3-174
single_pulse
-63.77
F3C
O
Ph
3r
50 40 30 20 10 0 -10 -20 -30 -40 -50 -60 -70 -80 -90 -100 -110 -120 -130 -140 -150 -160 -170 -180 -190 -200 -210 -220 -230 -240
f1 (ppm)

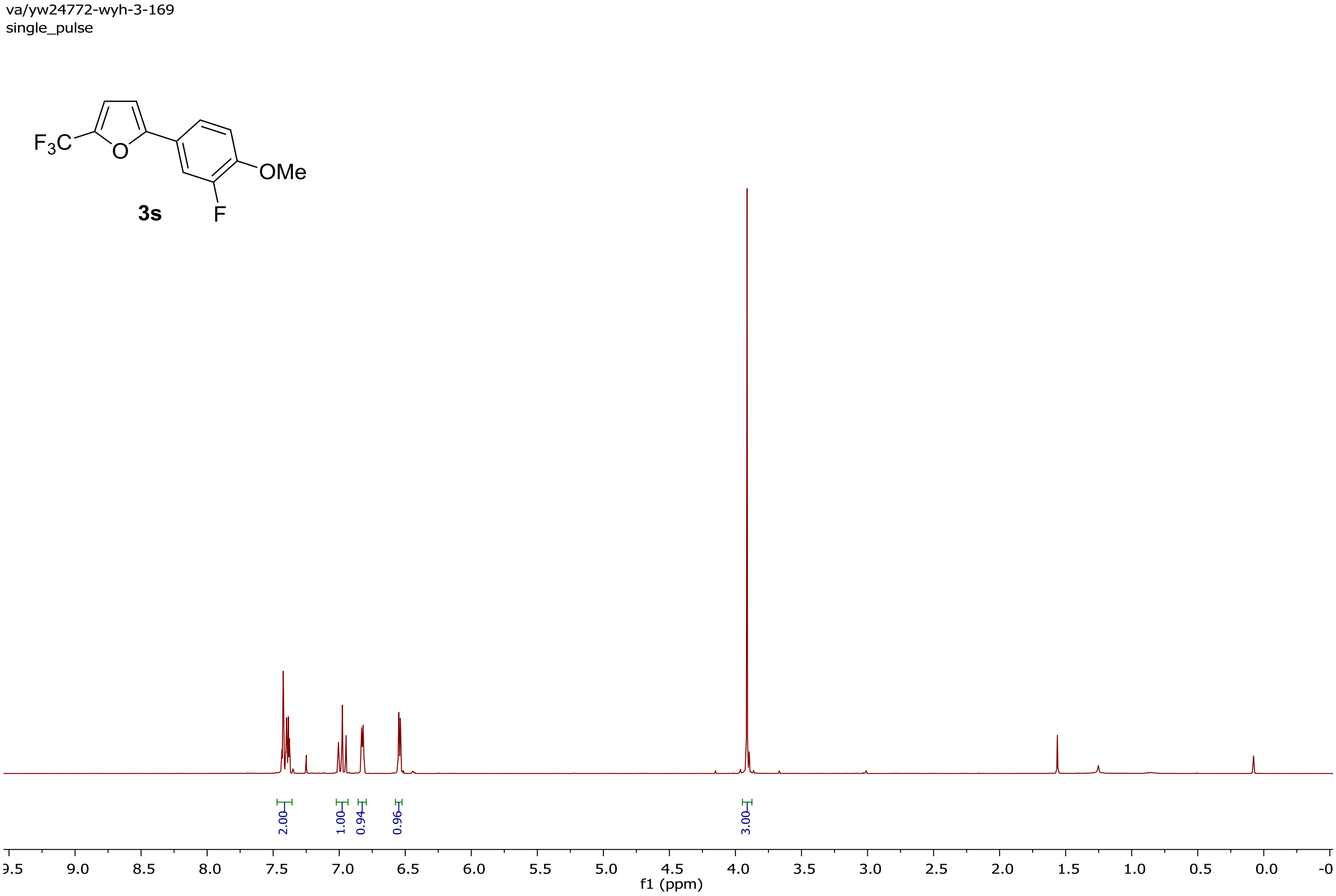
va/yw24772-wyh-3-169
single_pulse
F3C
O
OMe
F
3s
2.00
1.00
0.94
0.96
3.00
9.5 9.0 8.5 8.0 7.5 7.0 6.5 6.0 5.5 5.0 4.5 4.0 3.5 3.0 2.5 2.0 1.5 1.0 0.5 0.0
f1 (ppm)

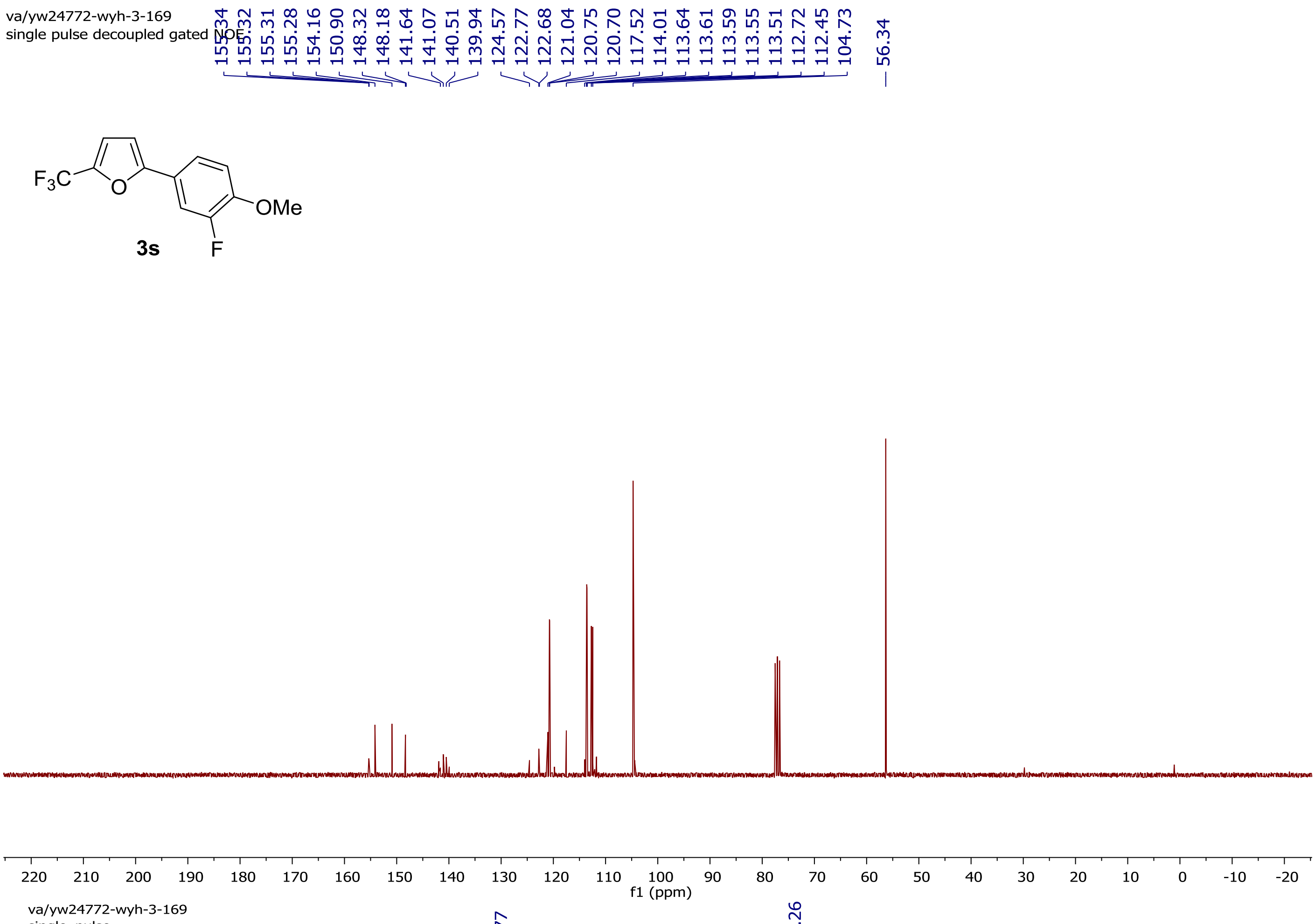
va/yw24772-wyh-3-169
single pulse decoupled gated NOE
155.34
155.32
155.31
155.28
154.16
150.90
148.32
148.18
141.64
141.07
140.51
139.94
124.57
122.77
122.68
121.04
120.75
120.70
117.52
114.01
113.64
113.61
113.59
113.55
113.51
112.72
112.45
104.73
56.34
F_3C
O
OMe
3s
F
220 210 200 190 180 170 160 150 140 130 120 110 100 90 80 70 60 50 40 30 20 10 0 -10 -20
f1 (ppm)

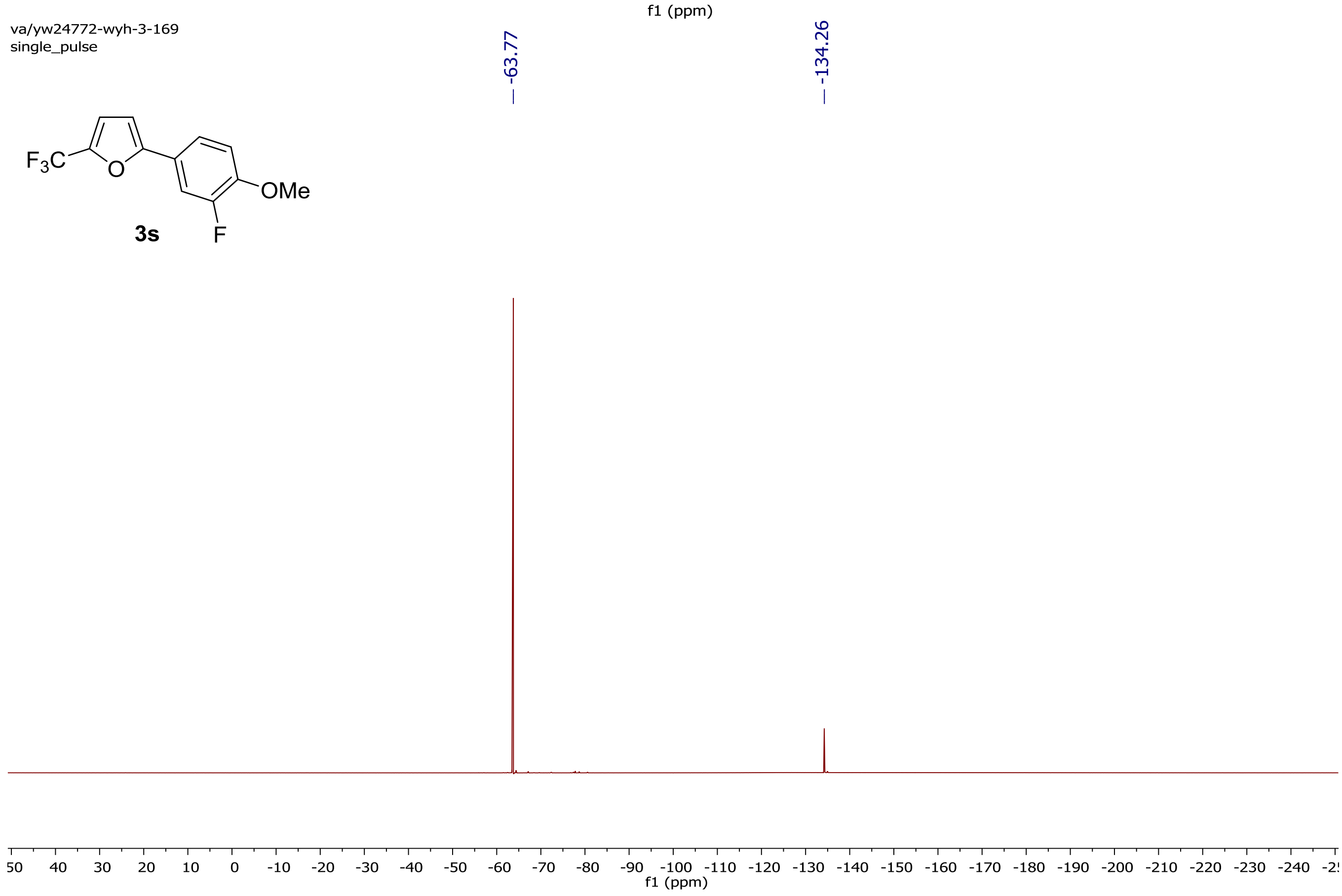
va/yw24772-wyh-3-169
single_pulse
-63.77
-134.26
F_3C
O
OMe
3s
F
50 40 30 20 10 0 -10 -20 -30 -40 -50 -60 -70 -80 -90 -100 -110 -120 -130 -140 -150 -160 -170 -180 -190 -200 -210 -220 -230 -240
f1 (ppm)

yw13155_wyh-4-42-1_PROTON01

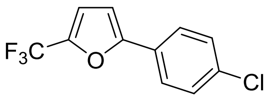

3t

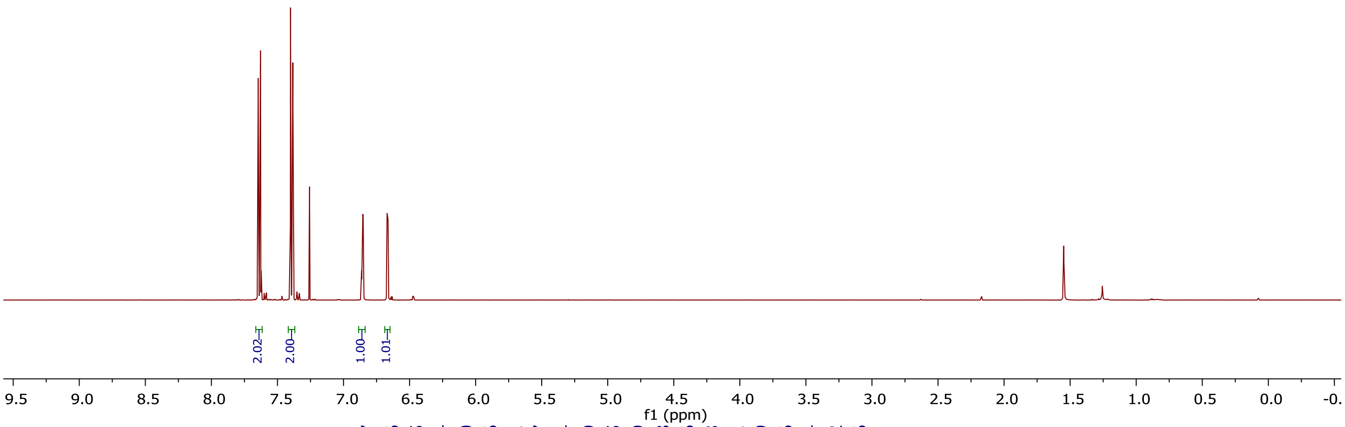

yw13155_wyh-4-42-1_CARBON01

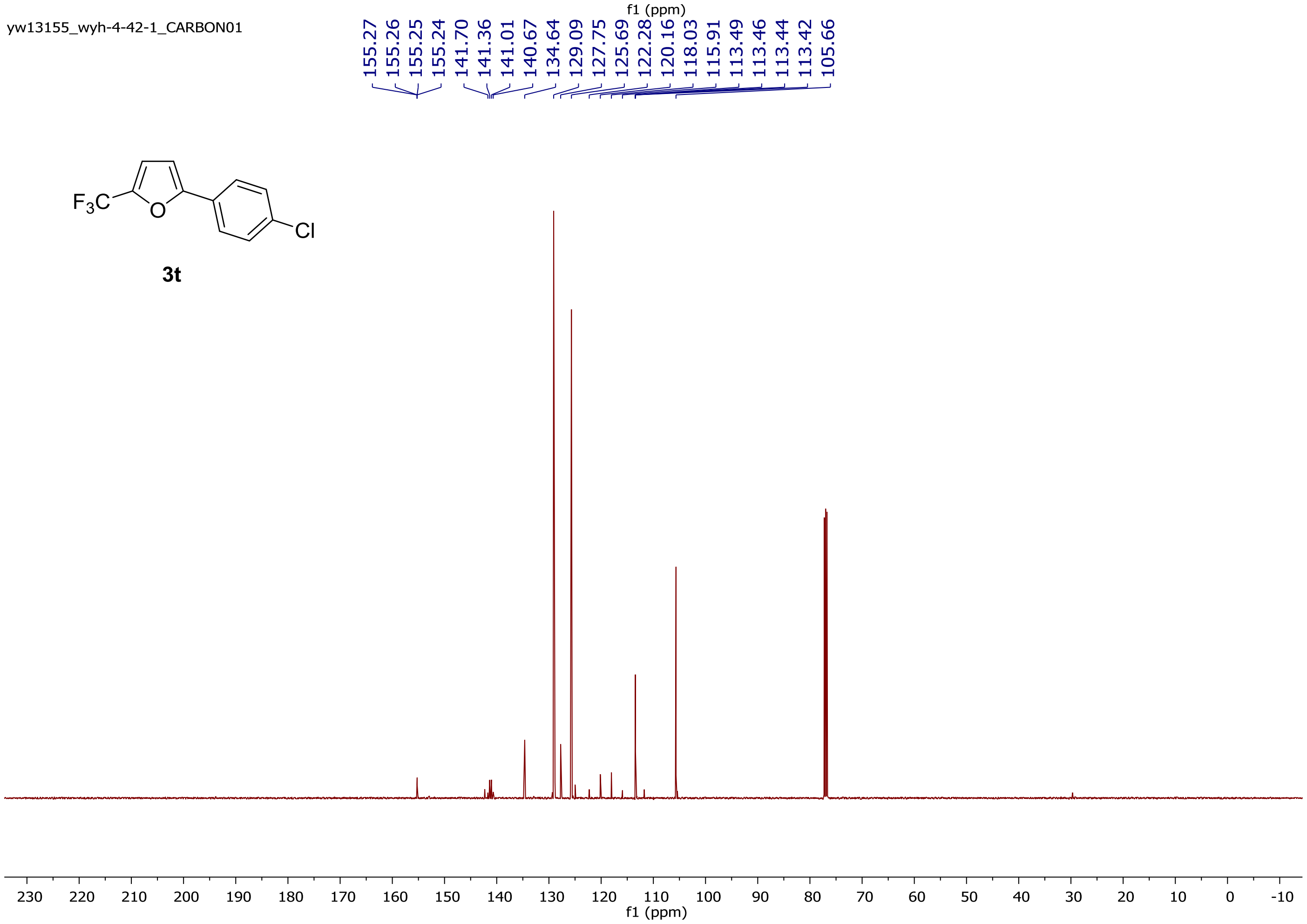

3t

va/yw53624-wyh-4-42
19F_single_pulse

— -63.83

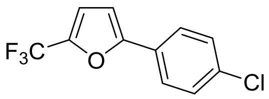

3t

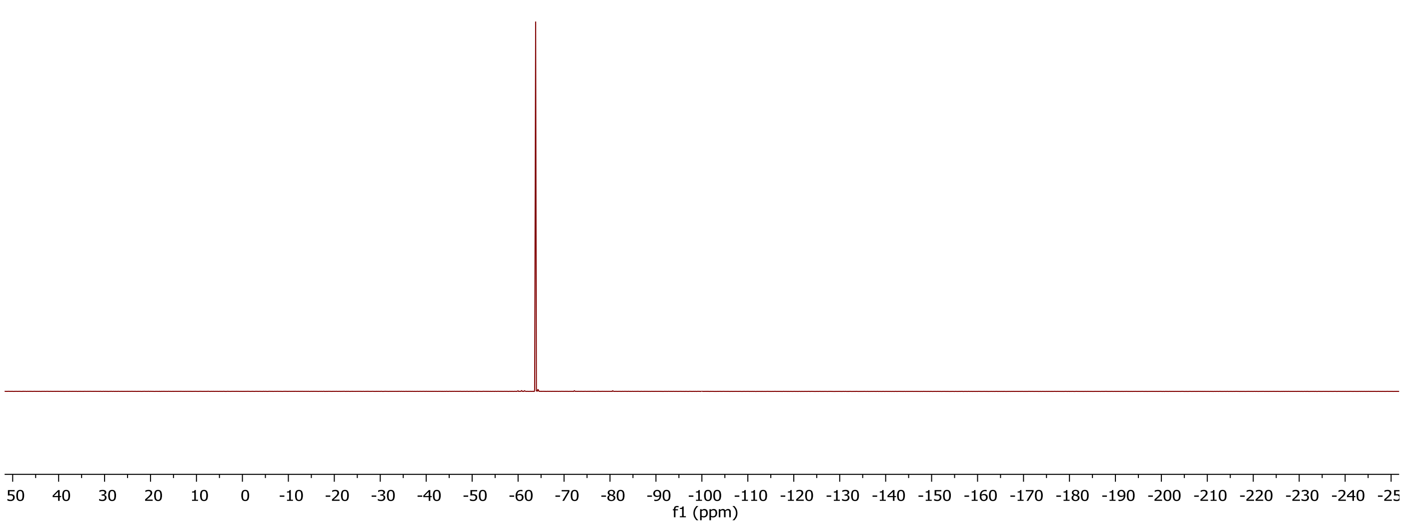

wyh-4-44

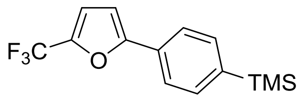

3u

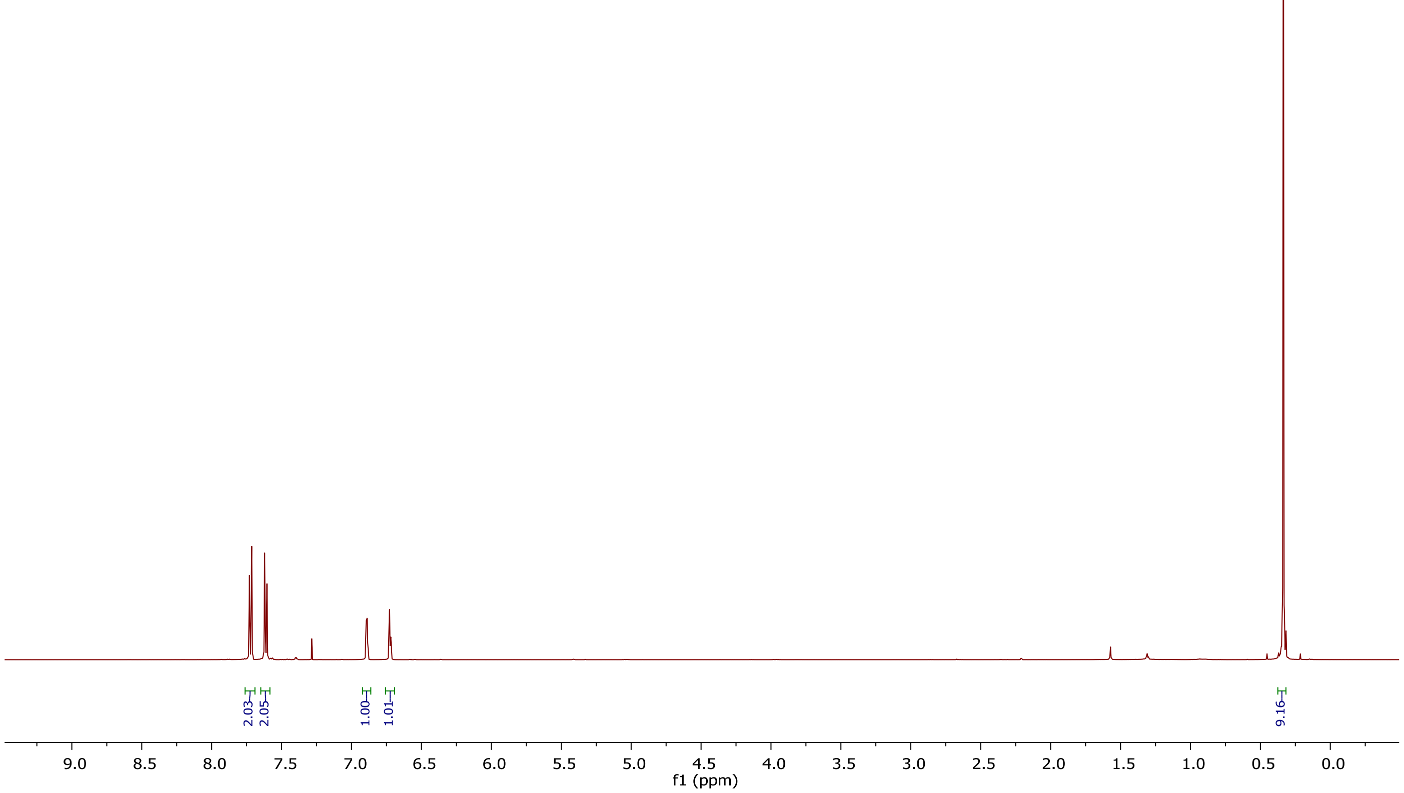

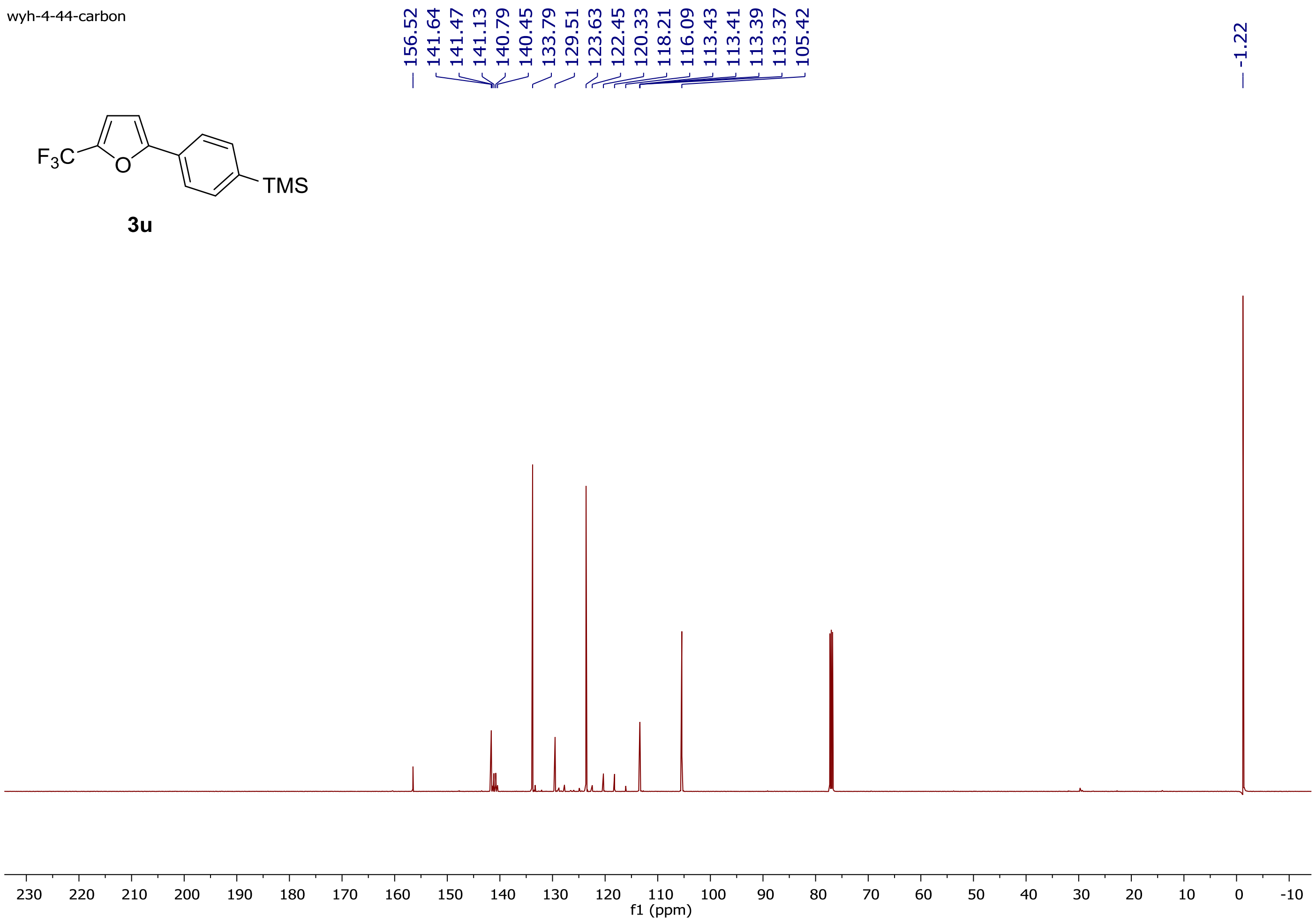
wyh-4-44-carbon
156.52
141.64
141.47
141.13
140.79
140.45
133.79
129.51
123.63
122.45
120.33
118.21
116.09
113.43
113.41
113.39
113.37
105.42
-1.22
F3C
O
TMS
3u
230 220 210 200 190 180 170 160 150 140 130 120 110 100 90 80 70 60 50 40 30 20 10 0 -10
f1 (ppm)

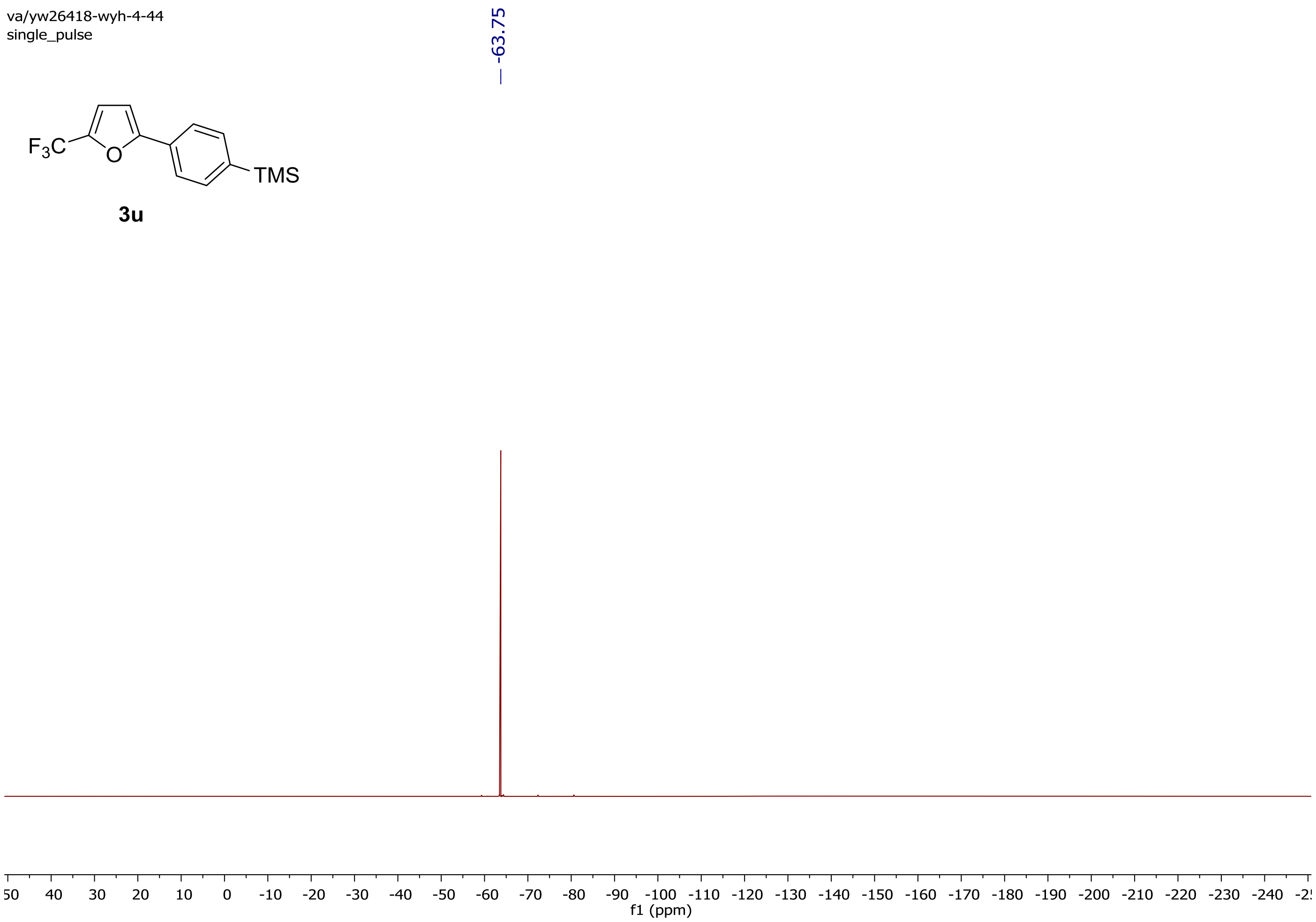
va/yw26418-wyh-4-44
single_pulse
-63.75
F3C
O
TMS
3u
50 40 30 20 10 0 -10 -20 -30 -40 -50 -60 -70 -80 -90 -100 -110 -120 -130 -140 -150 -160 -170 -180 -190 -200 -210 -220 -230 -240
f1 (ppm)

va/yw26200-wyh-4-32-n
single_pulse

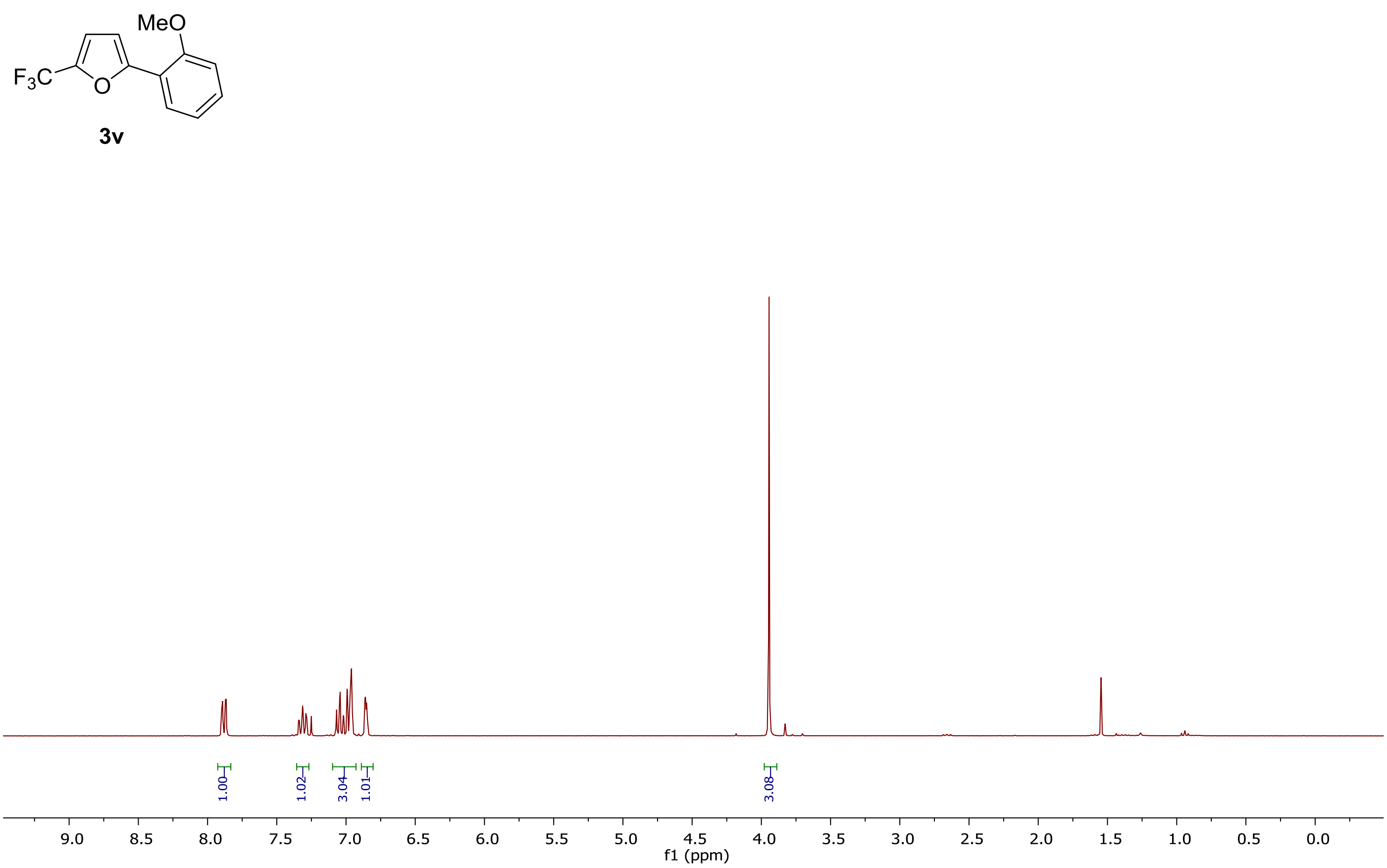

va/yw26200-wyh-4-32-n
single pulse decoupled gated NOE

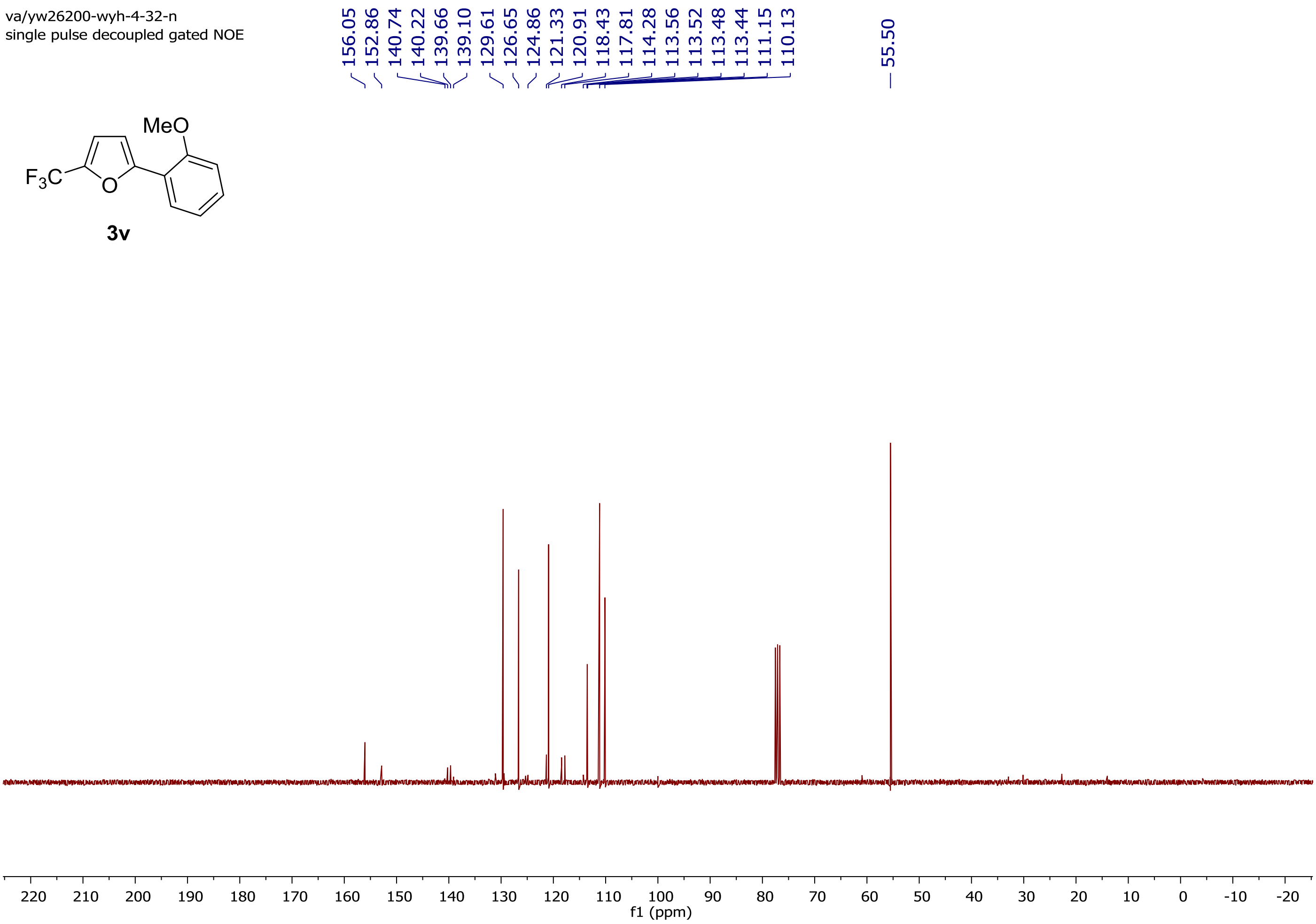

va/yw26200-wyh-4-32-n
single_pulse

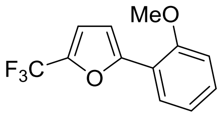

3v

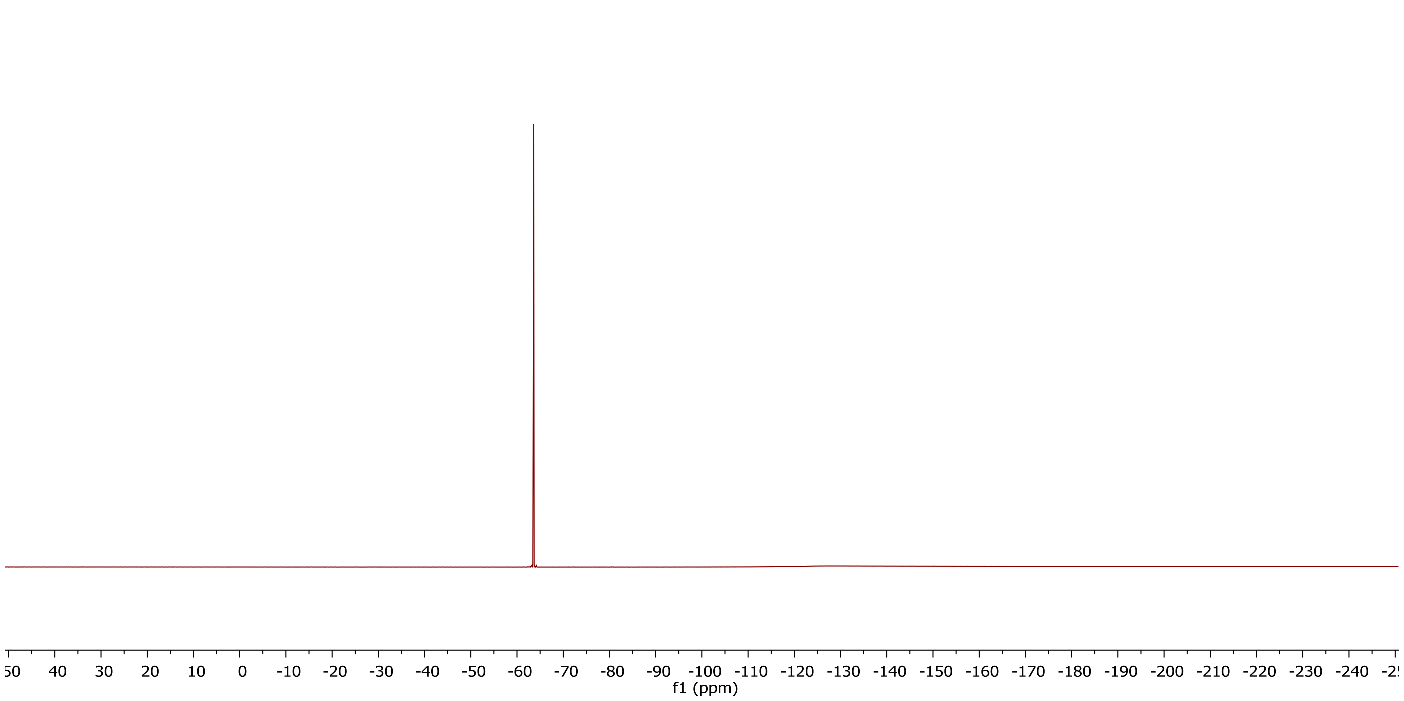

va/yw26108-wyh-4-26
single_pulse

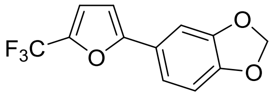

3w

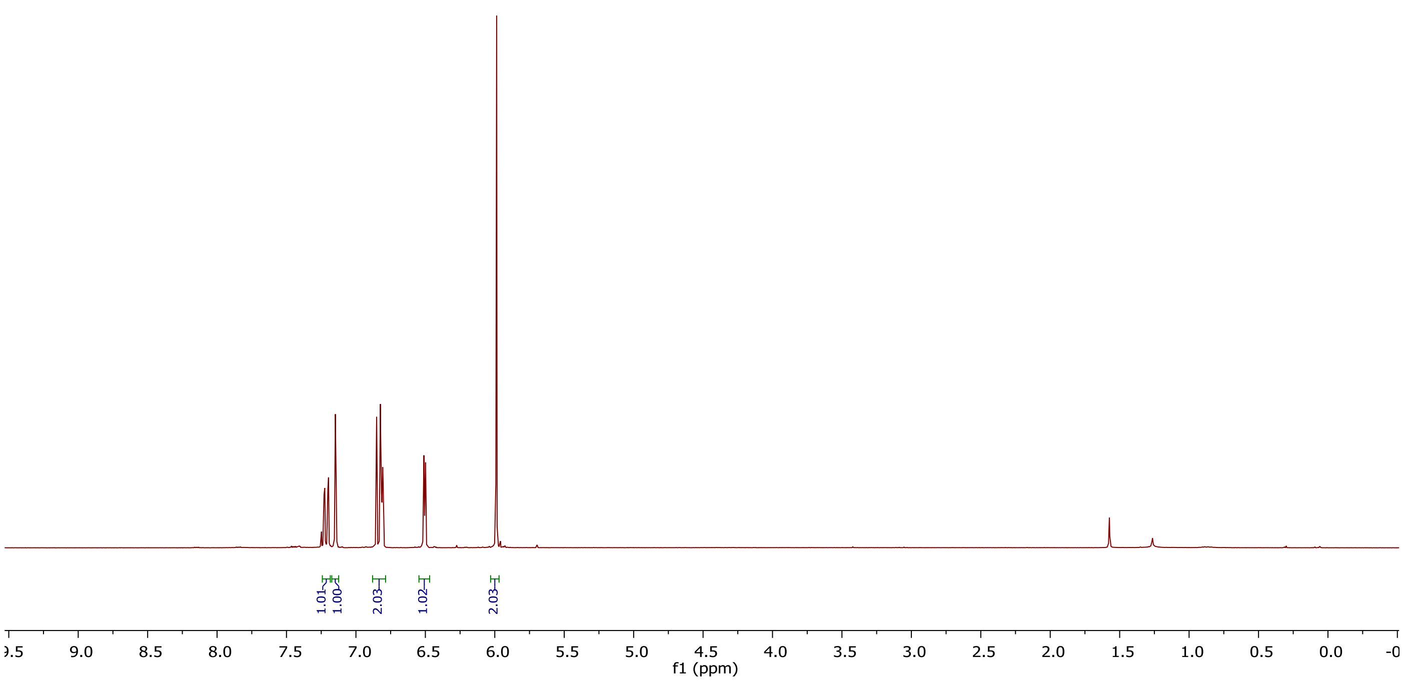

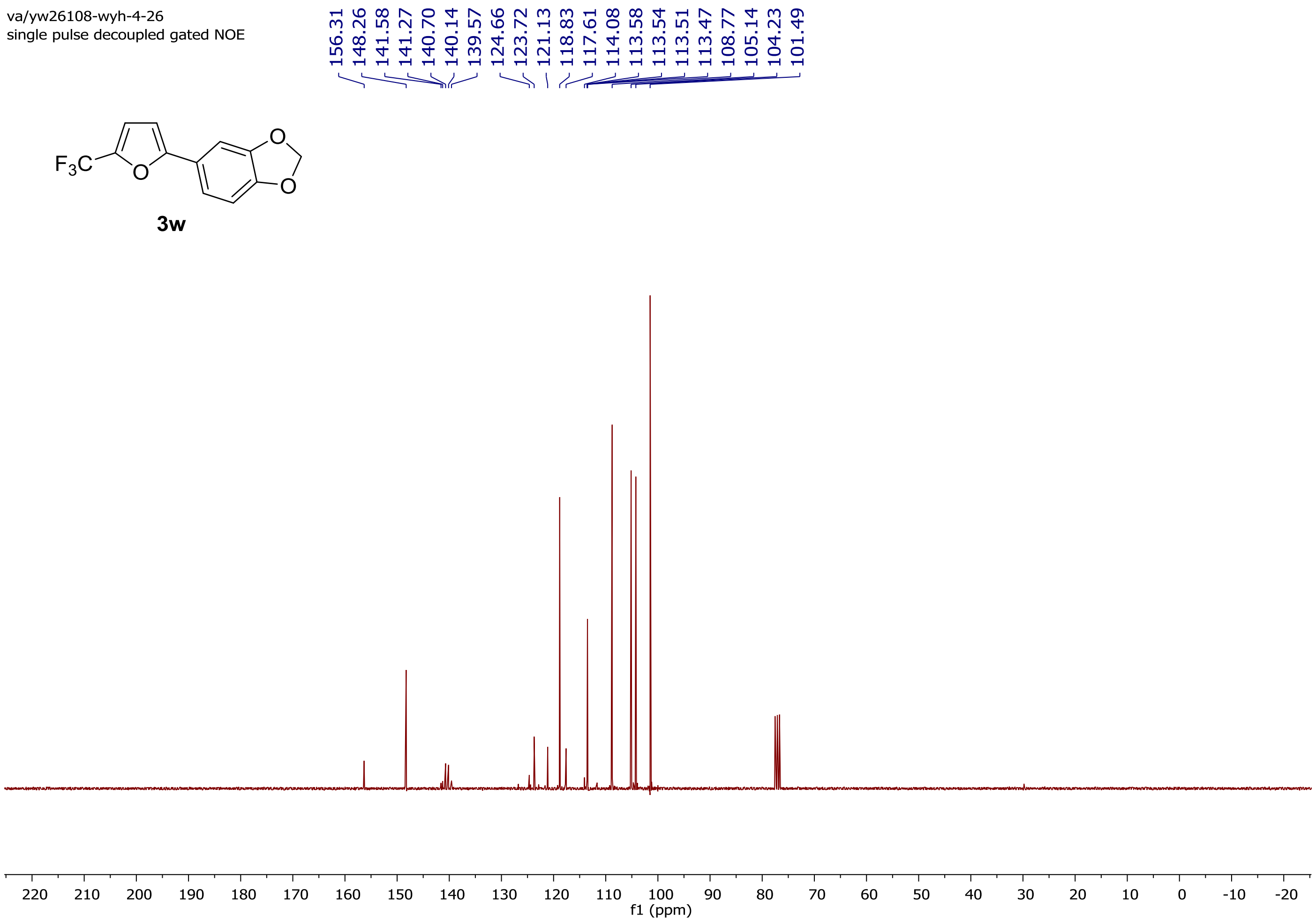
va/yw26108-wyh-4-26
single pulse decoupled gated NOE
156.31
148.26
141.58
141.27
140.70
140.14
139.57
124.66
123.72
121.13
118.83
117.61
114.08
113.58
113.54
113.51
113.47
108.77
105.14
104.23
101.49
F_3C
O
O
O
3w
220 210 200 190 180 170 160 150 140 130 120 110 100 90 80 70 60 50 40 30 20 10 0 -10 -20
f1 (ppm)

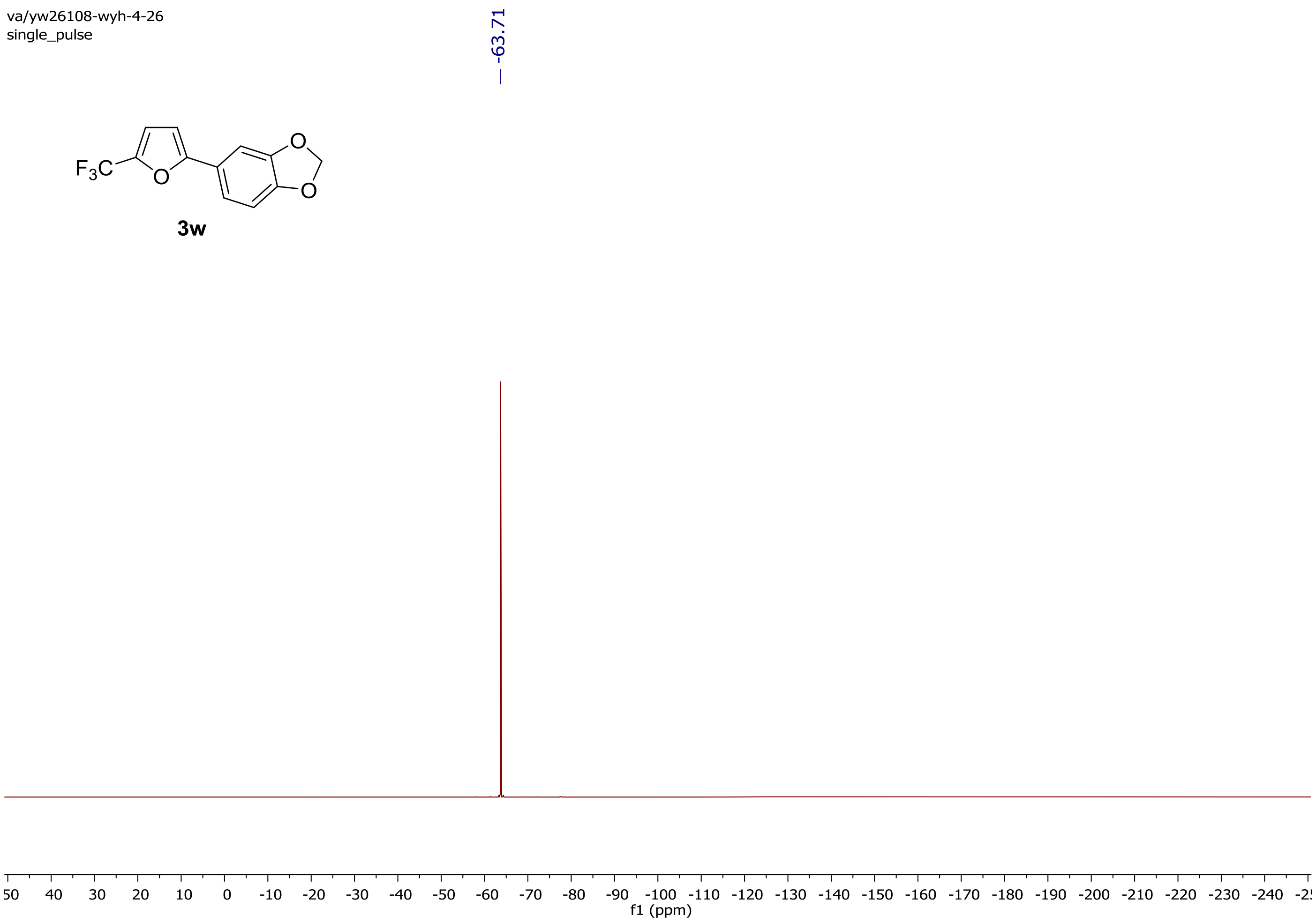
va/yw26108-wyh-4-26
single_pulse
-63.71
F_3C
O
O
O
3w
50 40 30 20 10 0 -10 -20 -30 -40 -50 -60 -70 -80 -90 -100 -110 -120 -130 -140 -150 -160 -170 -180 -190 -200 -210 -220 -230 -240
f1 (ppm)

va/yw27081-wyh-4-64
single_pulse

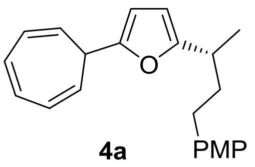

4a

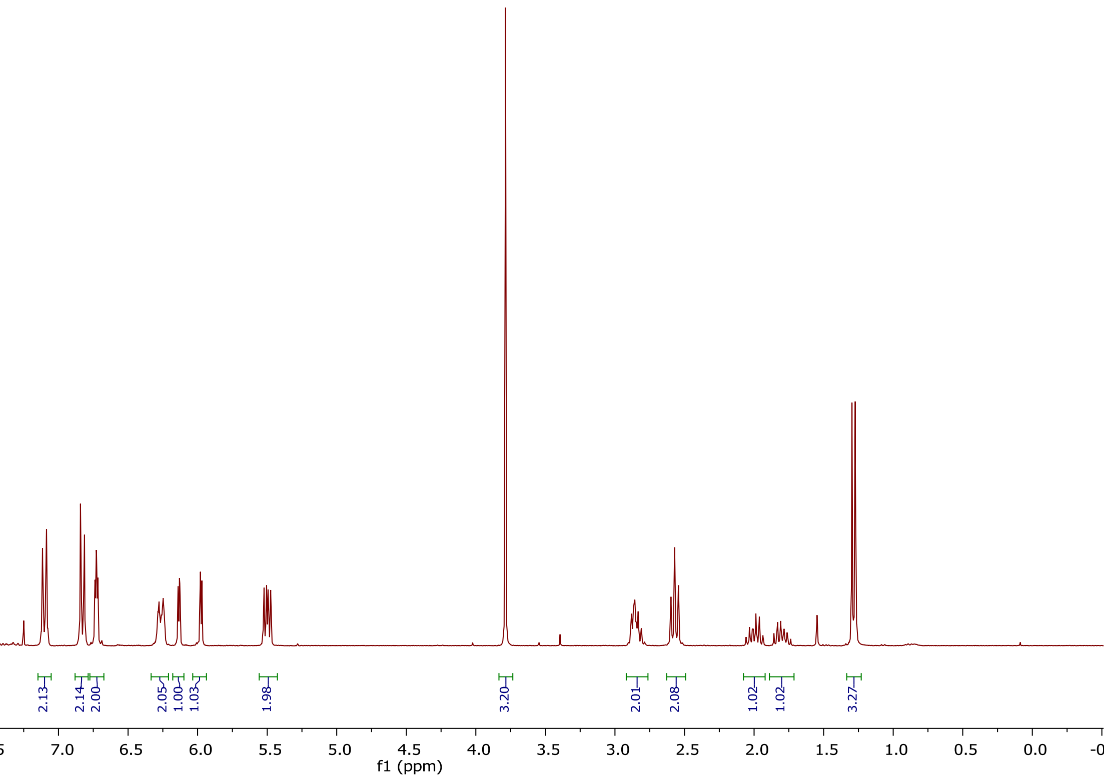

va/yw27081-wyh-4-64
single pulse decoupled gated NOE

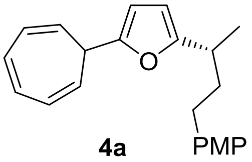

4a

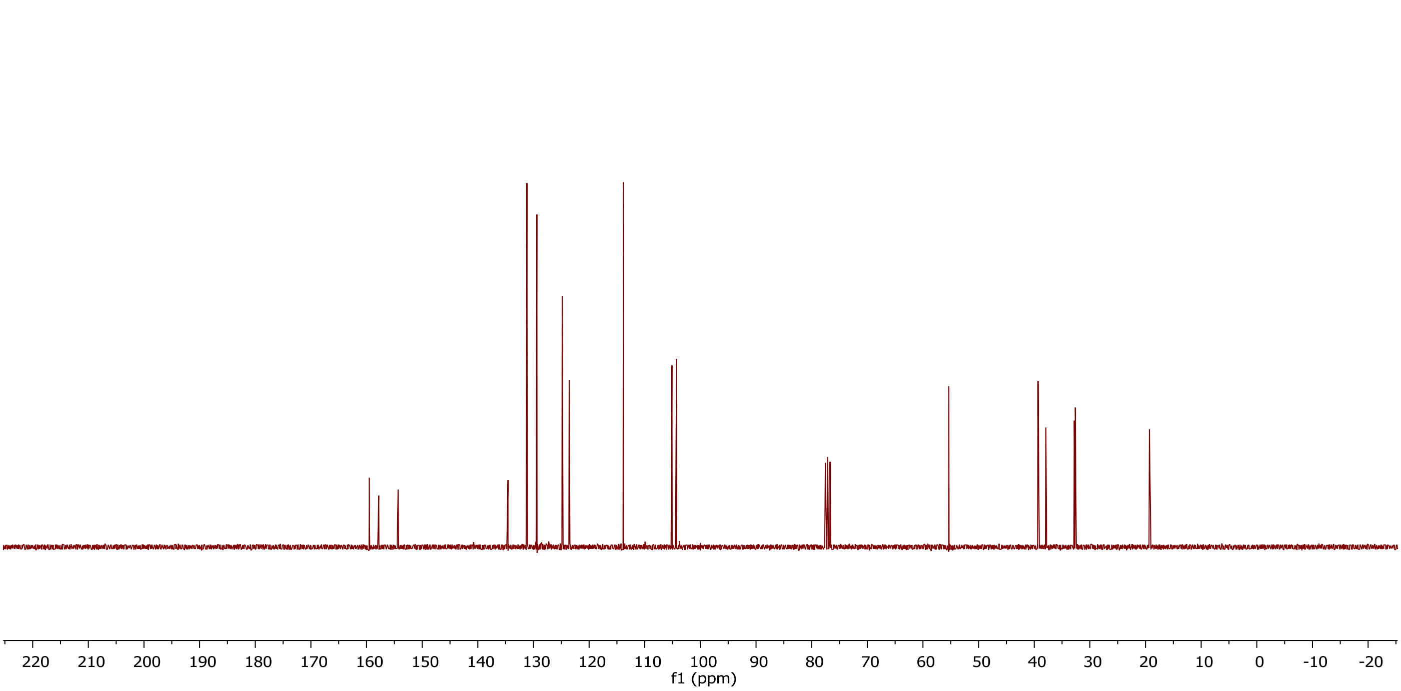

va/yw27115-wyh-4-67
single_pulse

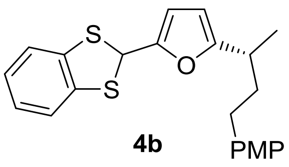

4b

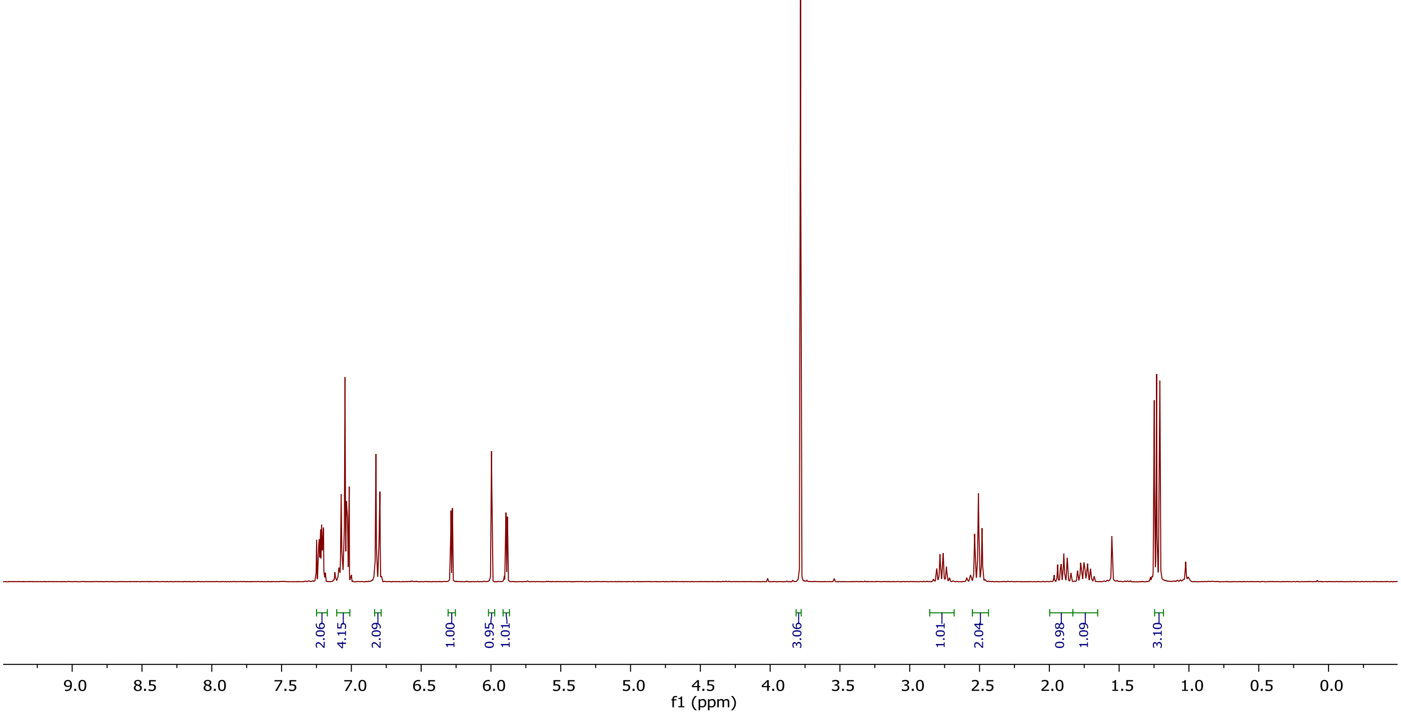

va/yw27115-wyh-4-67
single pulse decoupled gated NOE

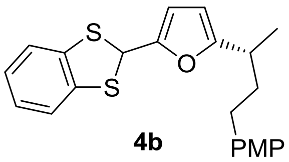

4b

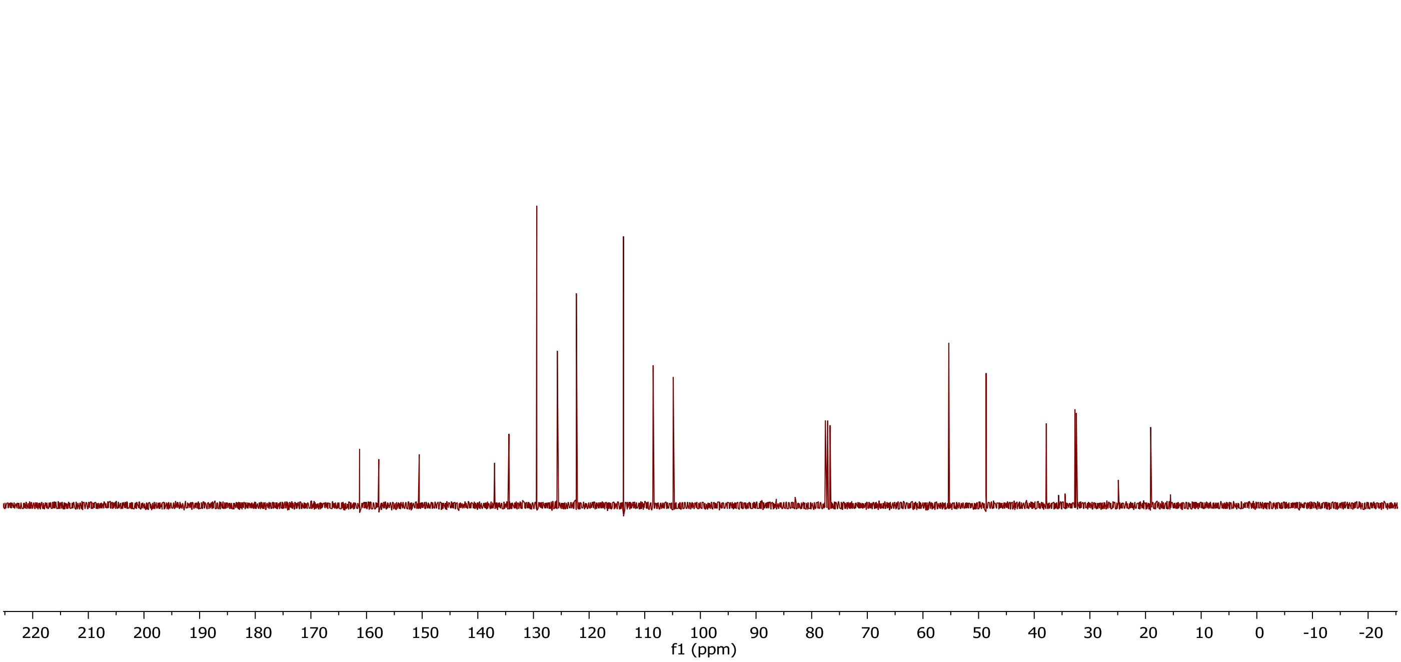

an12712_AN-4-756_PROTON01

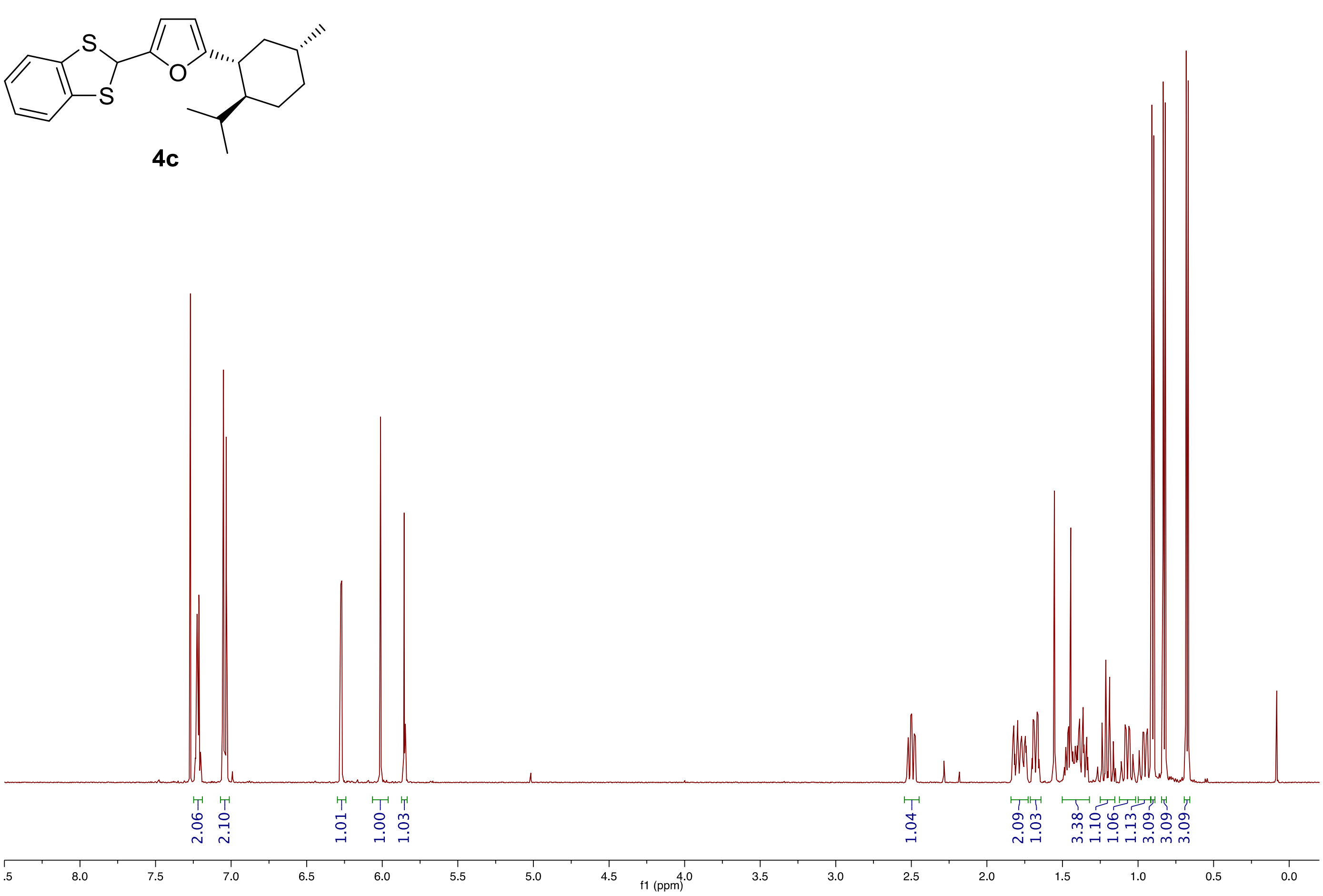

an12712_AN-4-756_CARBON01

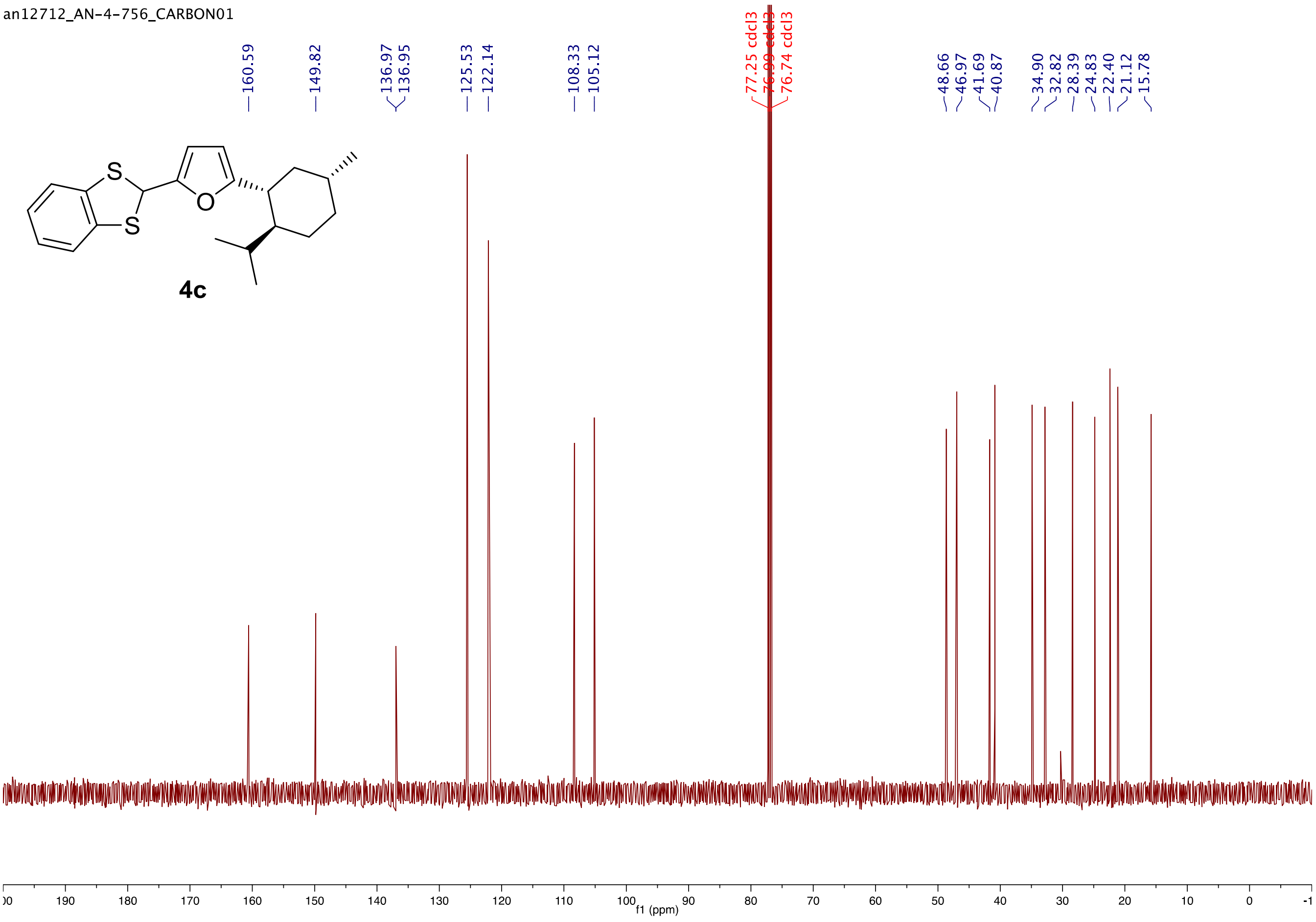

Supplement: Supplementary file 1 — Supplementary [file ANIE-56-1810-s001.pdf]
